# Iron accumulation drives fibrosis, senescence and the senescence-associated secretory phenotype

**DOI:** 10.1038/s42255-023-00928-2

**Published:** 2023-12-14

**Authors:** Mate Maus, Vanessa López-Polo, Lidia Mateo, Miguel Lafarga, Mònica Aguilera, Eugenia De Lama, Kathleen Meyer, Anna Sola, Cecilia Lopez-Martinez, Ines López-Alonso, Marc Guasch-Piqueras, Fernanda Hernandez-Gonzalez, Selim Chaib, Miguel Rovira, Mayka Sanchez, Rosa Faner, Alvar Agusti, Rodrigo Diéguez-Hurtado, Sagrario Ortega, Anna Manonelles, Stefan Engelhardt, Freddy Monteiro, Camille Stephan-Otto Attolini, Neus Prats, Guillermo Albaiceta, Josep M. Cruzado, Manuel Serrano

**Affiliations:** 1 Institute for Research in Biomedicine (IRB Barcelona), Barcelona Institute of Science and Technology (BIST), Barcelona, Spain; 2 Vall d’Hebron Institute of Oncology, Barcelona, Spain; 3 Departamento de Anatomía y Biología Celular, Universidad de Cantabria-IDIVAL, Santander, Spain; 4 Radiology Department, Hospital Universitari de Bellvitge, IDIBELL, L’Hospitalet de Llobregat, Barcelona, Spain; 5 Altos Labs, Cambridge Institute of Science, Cambridge, UK; 6 Nephrology and Renal Transplantation Research Group. Bellvitge Biomedical Research Institute (IDIBELL), Hospitalet de Llobregat, Barcelona, Spain; 7 Departamento de Biología Funcional, Instituto Universitario de Oncología del principado de Asturias, Universidad de Oviedo, Oviedo, Spain; 8 Unidad de Cuidados Intensivos Cardiológicos. Hospital Universitario Central de Asturias, Instituto de Investigación Sanitaria del Principado de Asturias, Oviedo, Spain; 9 CIBER-Enfermedades Respiratorias, Instituto de Salud Carlos III, Madrid, Spain; 10 Departamento de Morfología y Biología Celular, Universidad de Oviedo, Instituto de Investigación Sanitaria del Principado de Asturias, Oviedo, Spain; 11 Department of Pulmonary Medicine, Respiratory Institute, Hospital Clinic, IDIBAPS, University of Barcelona, Barcelona, Spain; 12 Iron Metabolism: Regulation and Diseases Group, Department of Basic Sciences, Universitat Internacional de Catalunya (UIC), Sant Cugat del Vallès, Spain; 13 Biomedicine Department, University of Barcelona, IDIBAPS, CIBERES, Barcelona, Spain; 14 Universitat de Barcelona, Institut Respiratori, Hospital Clinic, IDIBAPS, CIBERES, Barcelona, Spain; 15 Deparment of Tissue Morphogenesis, Max Planck Institute for Molecular Biomedicine, Münster, Germany; 16 Transgenics Unit, Spanish National Cancer Research Centre (CNIO), Madrid, Spain; 17 Nephrology Department, Bellvitge University Hospital, Hospitalet de Llobregat, Barcelona, Spain; 18 Department of Clinical Sciences, University of Barcelona, Hospitalet de Llobregat, Barcelona, Spain; 19 Institute of Pharmacology and Toxicology, Technical University of Munich (TUM), Munich, Germany; 20 DZHK (German Centre for Cardiovascular Research), Partner Site Munich Heart Alliance, Munich, Germany; 21 Institució Catalana de Recerca i Estudis Avançats (ICREA), Barcelona, Spain

**Keywords:** Ageing, Senescence, Mechanisms of disease, Metabolism, Iron

## Abstract

Fibrogenesis is part of a normal protective response to tissue injury that can become irreversible and progressive, leading to fatal diseases. Senescent cells are a main driver of fibrotic diseases through their secretome, known as senescence-associated secretory phenotype (SASP). Here, we report that cellular senescence, and multiple types of fibrotic diseases in mice and humans are characterized by the accumulation of iron. We show that vascular and hemolytic injuries are efficient in triggering iron accumulation, which in turn can cause senescence and promote fibrosis. Notably, we find that senescent cells persistently accumulate iron, even when the surge of extracellular iron has subdued. Indeed, under normal conditions of extracellular iron, cells exposed to different types of senescence-inducing insults accumulate abundant ferritin-bound iron, mostly within lysosomes, and present high levels of labile iron, which fuels the generation of reactive oxygen species and the SASP. Finally, we demonstrate that detection of iron by magnetic resonance imaging might allow non-invasive assessment of fibrotic burden in the kidneys of mice and in patients with renal fibrosis. Our findings suggest that iron accumulation plays a central role in senescence and fibrosis, even when the initiating events may be independent of iron, and identify iron metabolism as a potential therapeutic target for senescence-associated diseases.

## Abstract (web summary)

Iron is shown to have a central role in senescence, both by triggering senescence and through its accumulation in senescent cells, which is driving the senescence-associated secretory phenotype and, in turn, promotes fibrogenesis.

## Main

Fibrosis is the progressive replacement of healthy tissues by collagen-rich scar tissue^1^. It can affect any organ and manifest in the form of multiple diseases, such as cardiovascular diseases^2^, interstitial lung diseases (ILDs)^3^ or kidney disease^4^. Estimates are that fibrotic diseases account for 45% of all mortality in developed countries^5^. Fibrogenesis is initiated by damage to epithelial and/or endothelial cells, which start secreting chemokines and matrix-remodeling enzymes that facilitate the recruitment of macrophages and neutrophils^1^. Neutrophils and macrophages mount an inflammatory response that promotes further matrix remodeling through the secretion of matrix metalloproteinases (MMPs) and fibrogenic cytokines^6,7^. A key fibrogenic cytokine is transforming growth factor (TGF)-β, which is essential for the differentiation and survival of collagen-producing myofibroblasts^8,9^. While these events are initially reversible, progressive loss of capillarization due to suppression of vascular endothelial growth factor (VEGF) signaling may result in irreparable scar tissue and loss of tissue function^10–12^.

Fibrogenesis can be provoked by multiple external insults, such as toxins and infections, but in many fibrotic diseases the etiology remains unknown. Of note, fibrotic conditions initiated by an external trigger can keep progressing after cessation of the primary insult, as documented in acute kidney injury progressing to chronic kidney disease^13^. This suggests that the primary insult generates pathological entities that persist and are fibrogenic on their own. Multiple reports indicate that senescent cells are abundant in fibrotic tissues and directly contribute to the associated pathologies^14–17^. Senescent cells are damaged cells characterized by the upregulation of cell cycle inhibitors, more prominently CDKN2A (p16) and CDKN1A (p21), by the presence of high levels of reactive oxygen species (ROS), by a large expansion of the lysosomal compartment and by their abundant secretion of inflammatory cytokines, chemokines and matrix-remodeling enzymes, globally known as senescence-associated secretory phenotype (SASP)^18^. Cellular senescence can limit fibrogenesis when curtailing the proliferation of myofibroblasts^19–21^; however, when senescent cells accumulate in tissues, they can be fibrogenic on their own^14–17^ through profibrogenic factors present in the SASP, most notably, TGF-β, interleukin (IL)-11 and SERPINE1 (refs. ^15,22,23^).

It is well established that hereditary and acquired hemochromatosis, conditions characterized by the accumulation of iron in tissues, have a high predisposition to develop fibrotic diseases^24–29^. The association between iron accumulation and fibrosis has also been observed in fibrotic diseases unrelated to hemochromatosis^30–32^. Notably, iron accumulation has also been reported in senescent cells in vitro^33,34^. Here, we propose a unifying connection between cellular damage, iron accumulation, senescence and fibrosis.

## Results

### Fibrogenic injuries provoke progressive iron accumulation

**Fig. 1:**
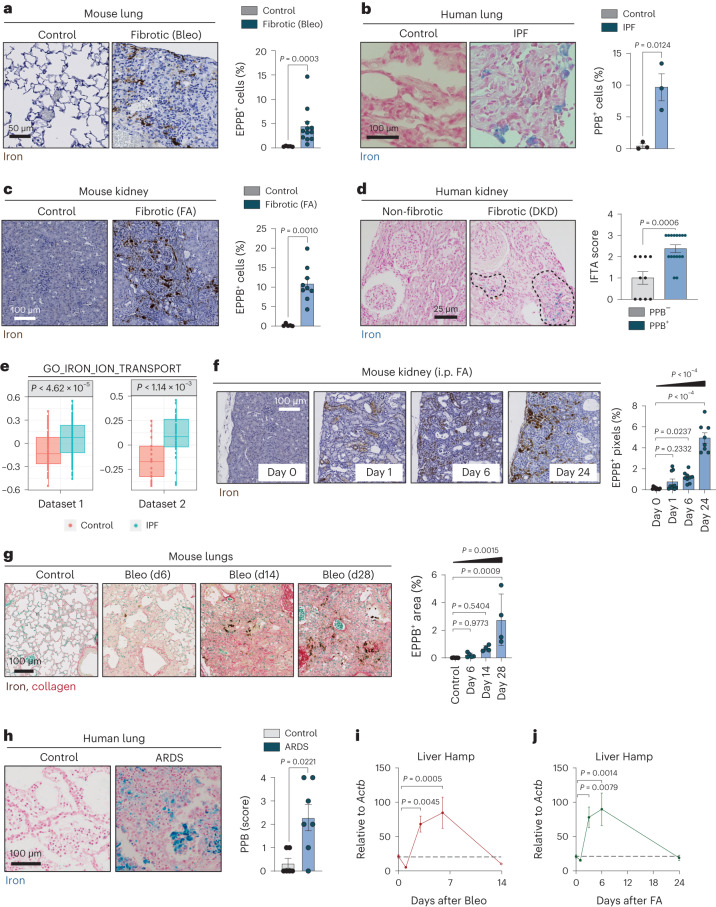
Fibrogenic injuries provoke progressive iron accumulation. **a**, Histochemistry for iron by enhanced Perl’s Prussian blue (EPPB) staining. Representative images of mouse lung sections 14 days post-intratracheal PBS (control; *n* = 5) or bleomycin (Bleo) (fibrotic; *n* = 12) and quantification. Scale bar, 50 μm. **b**, Histochemistry for iron by regular Perl’s Prussian blue (PPB) staining. Representative images of human lung sections from control non-cancerous lung tissue (control; *n* = 3) and patients with IPF (IPF; *n* = 3) and quantification. Scale bar, 100 μm. **c**, Histochemistry for iron by EPPB. Representative images of mouse kidney sections 28 days post-PBS (control; *n* = 5) or FA (fibrotic; *n* = 9) and quantification. Scale bar, 100 μm. **d**, Histochemistry for iron by regular PPB staining. Representative images of human kidney biopsies from patients with DKD without (non-fibrotic) or with interstitial fibrosis (fibrotic). Dashed lines delineate Perl’s-positive cells. Scale bar, 25 μm. Quantification shows IFTA scores (0, *n* = 4; 1, *n* = 4; 2, *n* = 10; and 3, *n* = 8) in patients with Perl’s-negative or Perl’s-positive kidney biopsies. **e**, Box plots of gene set variation analysis relative expression scores of GO_IRON_ION_TRANSPORT:0006826 pathway (*n* = 59 genes) computed on two different lung transcriptomes from controls (dataset 1, *n* = 78; and dataset 2, *n* = 17) and patients with IPF (dataset 1, *n* = 122; and dataset 2, *n* = 38). Boxes represent the median and 25th and 75th percentiles of data. The whiskers extend to the largest upper and lower values no further than 1.5 × interquartile range. Outlier points are plotted individually. We used the Kruskal–Wallis test. **f**, Histochemistry for iron by EPPB staining. Representative images of mouse kidneys from before (*n* = 10) and 1 day (*n* = 10), 6 days (*n* = 9) or 24 d (*n* = 8) post-FA and quantification. **g**, Histochemistry for iron and collagen by combining EPPB and Sirius Red/Fast Green (SRFG) staining. Representative images of mouse lung sections from before (*n* = 5) and 6 days (*n* = 5), 14 days (*n* = 4) or 28 days (n = 4) post-bleomycin and quantification. **h**, Histochemistry for iron by regular PPB staining. Representative images of human lung sections collected postmortem from control patients (no ARDS; *n* = 6) and patients with ARDS (*n* = 7) and quantification. **i**,**j**, mRNA levels of hepcidin (*Hamp*) relative to *Actb* in the livers of mice from before (*n* = 6) or 1 day (*n* = 5), 3 days (*n* = 5), 6 days (*n* = 4) or 14 days (*n* = 6) post-bleomycin (**i**) or from before (*n* = 6) and 1 day (*n* = 5), 3 days (*n* = 5), 6 days (*n* = 5) or 24 days (*n* = 6) post-FA (**j**). When bar graphs are shown, they represent mean ± s.e.m. Dots represent individual mice or human donors; all mouse experiments were performed at least three times with similar results; unless stated otherwise, we used a two-tailed Mann–Whitney *U*-test or, for multiple comparisons, a one-way analysis of variance (ANOVA); we analyzed for linear trends between the column means from left to right (**f**,**g**). Source data

**Extended Data Fig. 1:**
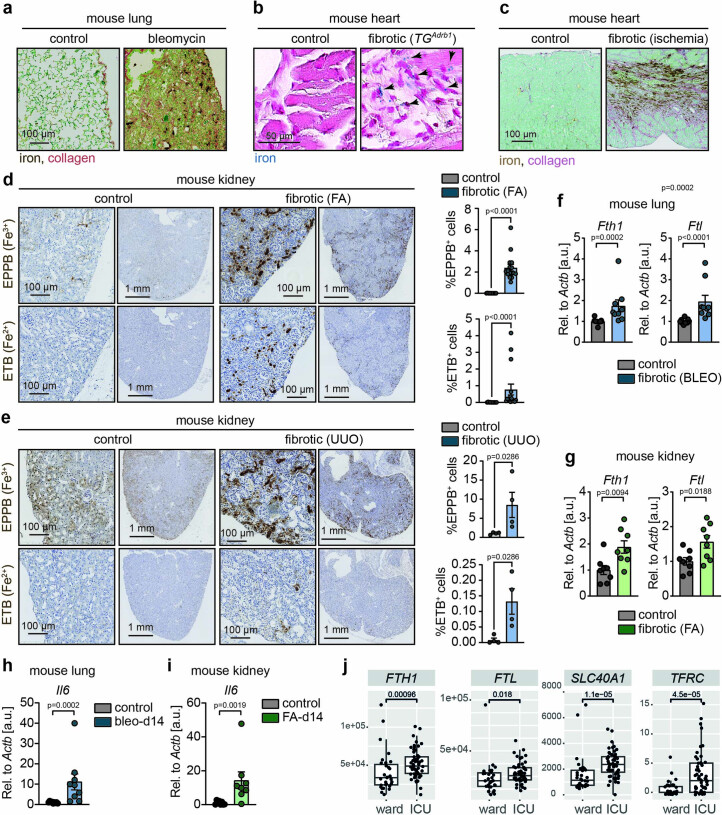
Fibrogenic injuries provoke progressive iron accumulation. **a**, Histochemistry for iron and collagen by combining enhanced Perl’s Prussian blue (PPB) and Sirius Red/Fast Green (SRFG) staining. Representative images of mouse lung sections 14 days post-PBS or bleomycin (1.5 U/kg of body weight). Scale bar, 100 μm. **b**, Histochemistry for iron by regular PPB staining. Representative images of mouse hearts from wild-type mice (control) and mice with transgenic overexpression of adrenergic receptor beta (fibrotic, *TG*^*ADRB1*^). Scale bar, 50 μm**. c**, Histochemistry for iron and collagen by combining enhanced PPB and SRFG staining. Representative images of mouse heart sections undergoing sham surgery (control), and from mice in which fibrosis was provoked by ischemic coronary artery ligation. Scale bar, 100 μm. **d-e**, Histochemistry for ferric (Fe^3+^) and ferrous iron (Fe^2+^) by enhanced Perl’s Prussian blue (EPPB) and enhanced Turnbull (ETB) staining respectively, in control and fibrotic mouse kidneys, where fibrosis was induced by **d**, folic acid (n_control_ = 10; n_FA_ = 16), or by **e**, unilateral ureteral obstruction (UUO) (n_control_ = 4; n_FA_ = 4). Representative images acquired by high magnification (scale bar, 50 μm) and low magnification (scale bar, 1 mm) and quantification. **f-g**, mRNA levels of *Fth1* and *Ftl* relative to *Actb*. **f**, in mouse lungs 14 days post- PBS (control; n = 8) or bleomycin (fibrotic, BLEO; n = 8), and **g**, in mouse kidneys 28 days post-PBS (control; n = 8) or folic acid (fibrotic, FA; n = 8). We used a two-sided, unpaired t-test. **h-i**, mRNA levels of *Il6* relative to *Actb*. **h**, in mouse lungs 14 days post-PBS (control; n = 9) or bleomycin (fibrotic, BLEO; n = 9), and **i**, in mouse kidneys 28 days post-PBS (control; n = 8) or folic acid (fibrotic, FA; n = 8). **j**, Comparison of RNA transcript levels of key iron homeostasis genes in the peripheral blood transcriptome of hospitalized COVID-19 patients separated into two groups: patients with clear symptoms of COVID-induced ARDS (hospitalized at the ICU; n = 56); and patients with no clear symptoms of ARDS (hospitalized at the ward; n = 31). Boxes represent the median and 25th and 75th percentiles of the data. The whiskers extend to the largest upper and lower values no further than 1.5 times the interquartile range. Outlier points are plotted individually. Means were compared by Wilcoxon signed-rank test. Where it is not stated otherwise, bar graphs represent mean ± SEM; individual values for each mouse or each individual are represented as points; mouse experiments were at least performed three times with similar results; unless stated otherwise, we used the Mann–Whitney test. Source data

We began by assessing whether iron accumulation is a general feature of fibrotic tissues using Perl’s Prussian blue staining, either regular or enhanced. We observed iron accumulation in murine lung fibrosis induced by intratracheal bleomycin (Fig. 1a and Extended Data Fig. 1a) and in the lungs of patients with idiopathic pulmonary fibrosis (IPF) (Fig. 1b). Iron accumulation was also evident in fibrotic hearts of mice with either transgenic overexpression of the β-adrenergic receptor (*Adrb1*) or ischemic heart disease caused by coronary artery ligation (Extended Data Fig. 1b,c). Similar observations were made in murine kidney fibrosis induced by intraperitoneal (i.p.) folic acid (FA) injection (Fig. 1c and Extended Data Fig. 1d) or unilateral ureteral obstruction (Extended Data Fig. 1e). Analyzing control and fibrotic kidneys from mice by enhanced Perl’s Prussian blue staining, which mainly recognizes ferric iron (Fe^3+^) and by enhanced Turnbull staining, which detects ferrous iron (Fe^2+^), showed that ferric iron was the dominant, although not exclusive, form accumulated in fibrosis (Extended Data Fig. 1d,e). In kidney biopsies from patients with diabetic kidney disease (DKD) the levels of Perl’s-positive cells were directly associated with the interstitial fibrosis and tubular atrophy (IFTA) score (Fig. 1d and Extended Data Table 1). Analysis of available clinical information suggested that the association between IFTA and iron accumulation was not due to comorbidities, treatments for iron deficiency or altered erythropoiesis as possible confounding factors (Supplementary Data Table 1). Fibrosis-associated iron accumulation was also reflected at the transcriptional level. Bleomycin-induced lung fibrosis and FA-induced kidney fibrosis resulted in substantially elevated messenger RNA levels of the iron-storage protein ferritin (*Fth1* and *Ftl*) (Extended Data Fig. 1f,g). We also analyzed whether a gene signature consisting of the major iron transporter and storage genes (Gene Ontology 0006826) was enriched in the lung transcriptome of controls and patients with IPF (two datasets from The Lung Tissue Research Consortium, GSE47460 (ref. ^35^)). Patients with IPF in both datasets showed a marked and significant increase in the iron transport signature (Fig. 1e), suggesting that altered gene expression of iron uptake and storage genes is a defining feature of IPF.

We assessed the kinetics of iron accumulation throughout fibrogenesis. Iron deposition visualized by enhanced Perl’s Prussian blue staining was detectable in the kidneys of FA-treated mice as early as 1 d after the fibrogenic insult and kept on progressing until the last day of analysis (day 28) (Fig. 1f). We made similar observations in the lungs of bleomycin-treated mice and found a progressive increase in the abundance of cells with high iron levels, which was generally associated with areas of abnormal collagen accumulation (Fig. 1g). To understand how early iron accumulation starts in the context of human lung fibrosis, we investigated tissues from patients with acute respiratory distress syndrome (ARDS), a condition that can be caused by multiple independent insults and, in around 20% of cases, develops into established lung fibrosis^36^. Lungs were analyzed within a few days after ARDS, before the potential development of fibrosis. Notably, iron accumulation was observed in most of the lungs irrespective of the type of lung injury that caused ARDS (septic shock in association with pneumonia or kidney transplantation, hemorrhagic shock in association with aortic aneurysm, acute leukemia with systemic mycosis, pneumonia in association with influenza A or of unknown origin)^37^ (Fig. 1h and Extended Data Table 2).

We wondered whether the progressive accumulation of iron in damaged tissues had a correlate that was detectable systemically. Mice treated with intratracheal bleomycin or i.p. FA presented a rapid but transient peak of hepatic hepcidin (*Hamp*, measured as mRNA) (Fig. 1i,j), a hormone that is induced in the liver when iron is in excess^38^. Hepcidin is co-regulated by iron and inflammatory cytokines, such as IL-6, BMP2 and BMP6^38^. Indeed, in addition to iron accumulation, fibrosis induced by bleomycin in the lung or by FA in the kidney was also associated with robust induction of IL-6 (Extended Data Fig. 1h,i). The systemic iron response was also detectable in human patients undergoing an acute damage response. In particular, in the case of ARDS, we detected deregulated iron homeostasis in the transcriptome of peripheral blood^39^ (Extended Data Fig. 1j). We conclude that iron accumulation can be triggered by injury and can progress throughout fibrosis in a variety of animal models and in human patients. Of note, a systemic response to iron excess is detectable transiently during the initiation of fibrogenesis.

### Iron accumulation promotes fibrogenesis and senescence

**Fig. 2:**
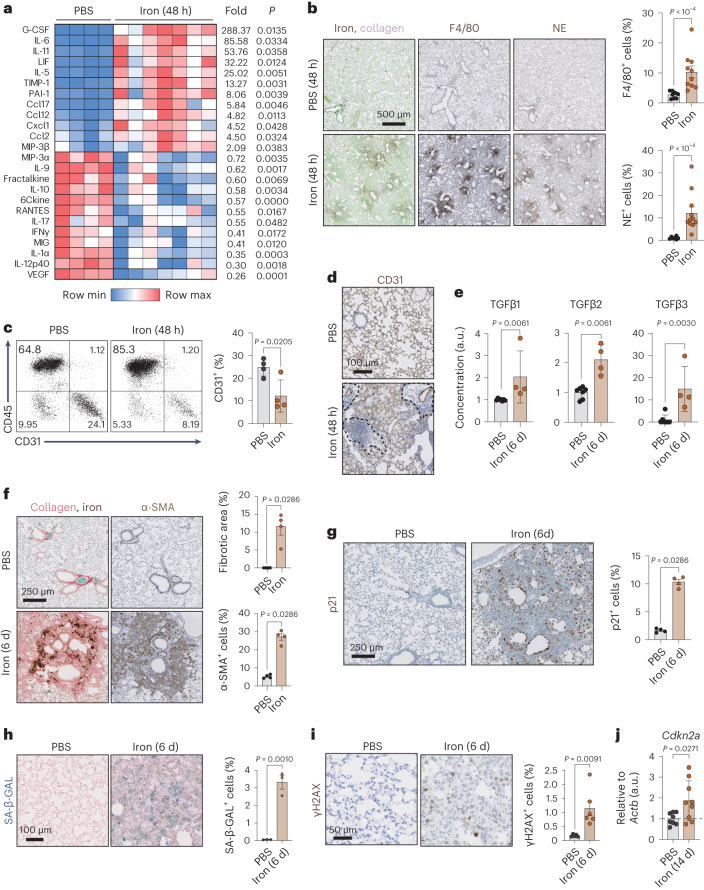
Iron accumulation promotes fibrogenesis and senescence. **a**, Heat map showing the protein levels of cytokines and chemokines significantly altered 2 d post-iron (500 nM; *n* = 7), when compared to controls (PBS; *n* = 4) in the lungs lysates of mice tested by a multiplex protein assay. **b**, Histochemistry for iron and collagen, by combining EPPB and SRFG staining, immunohistochemistry for the macrophage marker F4/80 and for the neutrophil marker, neutrophil elastase (NE). Representative images of mouse lung sections 2 d post-PBS (*n* = 8 for F4/80; *n* = 7 for NE) or iron (500 nM; *n* = 10 for F4/80; *n* = 11 for NE) and quantification. Scale bar, 500 μm. **c**, Flow-cytometric analysis of CD31 and CD45 in lung cells from mice 2 d post-PBS (*n* = 4) or iron (500 nM; *n* = 4) and quantification. **d**, Representative images showing immunohistochemical detection of CD31^+^ endothelial cells in lung sections of mice 2 d post-PBS (*n* = 4) or iron (500 nM; *n* = 5). Dashed lines delineate regions where CD31 staining gets lost. Scale bar, 100 μm. **e**, Protein levels of TGF-β family members in the lungs of mice 6 days post-PBS (*n* = 7) or iron (500 nM; *n* = 4) detected by a multiplex protein assay. **f**, Histochemistry for iron and collagen, by combining EPPB and SRFG staining and immunohistochemistry for the myofibroblast marker α-SMA. Representative images of mouse lung sections 6 days post-PBS (*n* = 4) or iron (500 nM; *n* = 4) and quantification. Scale bar, 250 μm. **g**, Immunohistochemistry for the senescence marker p21. Representative images of mouse lung sections 6 days post-PBS (*n* = 4) or iron (500 nM; n = 4) and quantification. Scale bar, 250 μm. **h**, SA-β-GAL staining of mouse lung sections 6 days post-PBS (*n* = 3) or iron (500 nM; *n* = 3). Representative images and quantification. Scale bar, 100 μm. **i**, Immunohistochemistry for the DNA-damage marker γH2AX. Representative images of mouse lung sections 6 days post-PBS (*n* = 5) or iron (500 nM; *n* = 6) and quantification. Scale bar, 50 μm. **j**, mRNA levels of *Cdkn2a* (p16) relative to *Actb* in mouse lungs 14 d post-PBS (control; *n* = 8) or iron (500 nM; *n* = 9). When bar graphs are shown, they represent mean ± s.e.m.; dots represent individual mice. All experiments were performed at least three times with similar results. Data were analyzed by a two-tailed unpaired *t*-test (**a**,**c**,**h**–**j**) and a two-tailed Mann–Whitney *U*-test (all remaining panels) or for multiple comparisons, a one-way ANOVA. Source data

**Extended Data Fig. 2:**
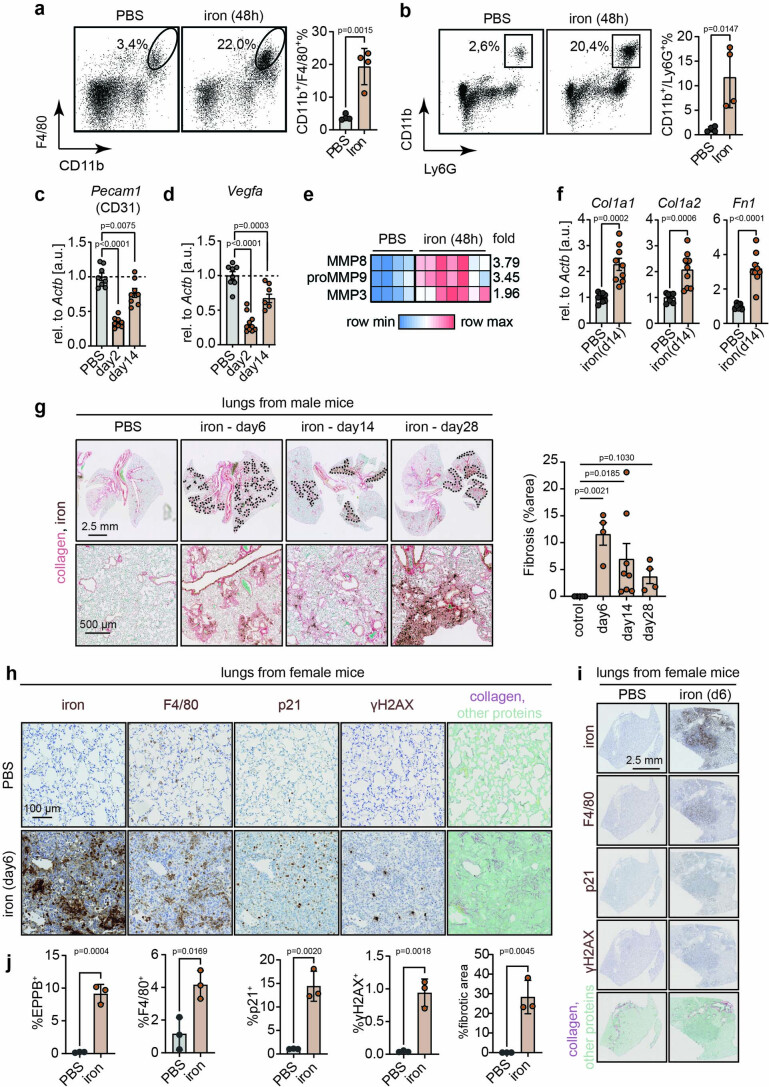
Iron accumulation promotes fibrogenesis and senescence. **a-b**, Flow-cytometric analysis of dissociated lung tissues for the **a**, macrophage markers CD11b and F4/80, and **b**, the neutrophil markers CD11b and Ly6G. Representative dot plots of lung cells from mice 2 days post-in PBS (n = 4) or iron (500 nM; n = 4), and quantification. **c-d**, mRNA levels of **c**, *Pecam-1* (CD31) and **d**, *Vegfa* relative to *Actb* in mouse lungs 2 days post-PBS (n = 8) or 2 days (n = 8-10) and 14 days (n = 6-8) post-iron (500 nM). **e**, Heat map showing protein expression of significantly altered matrix metalloproteinases (MMP) in the lungs of mice 2 days post-PBS (n = 4) or iron (500 nM; n = 7), measured by a multiplex protein assay; fold change to the right. **f**, mRNA levels of *Col1a1*, *Col1a2*, *and Fn1* relative to *Actb* in mouse lungs 14 days post-PBS (n = 8) or iron (500 nM; n = 9). **g**, Histochemistry for iron and collagen, by combining enhanced Perl’s Prussian blue and Sirius Red/Fast Green staining. Representative images of mouse lung sections from male mice 2 days post-intratracheal administration of PBS (control; n = 5) or 6 days (n = 4), 14 days (n = 8), and 28 days (n = 4) after intratracheal administration of iron (500 nM; n = 4), and quantification of fibrotic area. Scale bar 2.5 mm for low magnification, and 500 μm for high magnification images. Dashed lines delineate iron and collagen-rich areas. We used the Kruskal–Wallis test. **h-j**, Histochemistry for iron by enhanced Perl’s Prussian blue staining, and immunohistochemistry for F4/80, p21, γH2AX, and histochemical detection of collagen by Sirius red/Fast green staining. **h**, High magnification (scale bar, 100 μm) and **i**, low magnification (scale bar, 2.5 mm) representative images of mouse lung sections from female mice 6 days post-PBS (control; n = 3) or iron (500 nM; n = 3), and **j**, quantification. Where it is not stated otherwise, bar graphs represent mean ± SEM; individual values for each mouse are represented as points; mouse experiments were at least performed three times with similar results; unless stated otherwise, compared distributions using two-tailed unpaired t-test, for multiple comparisons, we used one-way ANOVA. Source data

We wondered whether iron accumulation is a bystander phenomenon or plays a causative role in fibrogenesis. To address this question, we delivered free iron intratracheally to mice in a concentration that approximates the estimated local iron concentration of red blood cells (RBCs) immediately after their hemolysis and release of iron from hemoglobin. Intratracheal delivery of iron into male mice provoked a robust pro-fibrotic cytokine response within 2 days after injury, as measured by a multiplex protein detection assay (Fig. 2a). This response was dominated by granulocyte colony-stimulating factor (G-CSF), a recruitment factor for fibroblasts and granulocytes^40^; members of the pro-fibrotic IL-6 family^41,42^, including IL-6, IL-11 and LIF; TIMP-1, a collagenase inhibitor that prevents degradation of newly formed collagen^43^; PAI-1 (SERPINE1), a master regulator of collagen deposition in fibrotic diseases^44^; the eosinophil chemoattractant IL-5 (ref. ^45^); macrophage chemoattractants CCL2 and CCL12; and the neutrophil chemoattractant CXCL1 (refs. ^1,46,47^) (Fig. 2a). Accordingly, we observed a massive influx of macrophages (Fig. 2b and Extended Data Fig. 2a) and neutrophils (Fig. 2b and Extended Data Fig. 2b) in iron-rich foci. Notably, iron also induced depletion of CD31^+^ endothelial cells as detected by flow cytometry (Fig. 2c) and mRNA levels (Extended Data Fig. 2c). Histological analyses showed that loss of CD31^+^ cells mainly affected foci with immune infiltration (Fig. 2d). Depletion of endothelial cells was concomitant with a robust and persistent suppression of VEGF protein and mRNA levels (Fig. 2a and Extended Data Fig. 2d), which is reminiscent of fibrosis-associated vascular rarefaction^10,11,48^. Iron also provoked the remodeling of the extracellular matrix, first visible within 2 d through the increased abundance in TIMP-1 (Fig. 2a) and MMPs (MMP-8, MMP-9 and MMP-3) (Extended Data Fig. 2e). This was followed by the upregulation of protein levels of the profibrogenic TGF-β family members within 6 days of iron delivery, as measured by a multiplex protein detection assay (Fig. 2e). At this stage, iron-rich foci in the lungs presented with a marked expansion of α-SMA-positive myofibroblasts, associated with a robust accumulation of collagen (Fig. 2f). Two weeks after iron delivery, *Col1a1* and *Col1a2* (collagens) and *Fn1* (fibronectin) mRNA levels were significantly elevated (Extended Data Fig. 2f). Iron-rich lesions associated with fibrosis persisted until the last day of analysis (day 28) (Extended Data Fig. 2g). Considering the strong association between fibrosis and cellular senescence^14^, we tested whether intratracheal iron could induce senescence. Iron triggered a remarkable elevation of senescence-associated β-galactosidase (SA-β-GAL) signal, p21^+^ cells and the DNA-damage marker γH2AX in the fibrotic lesions 6 days post-delivery (Fig. 2g–i). Two weeks post-insult, mice treated with iron presented elevated levels of *Cdkn2a* (p16) mRNA in the lungs (Fig. 2j). The fibrogenic and senogenic effects of iron were not sex specific, as delivering iron intratracheally into female mice, produced similar results to those obtained in male mice (Extended Data Fig. 2h–j). Together, we conclude that intratracheal iron administration is sufficient to initiate the hallmarks of fibrogenesis, including collagen deposition, senescence, vascular rarefaction, inflammation and innate immune infiltration.

### Vascular insults provoke iron accumulation

**Fig. 3:**
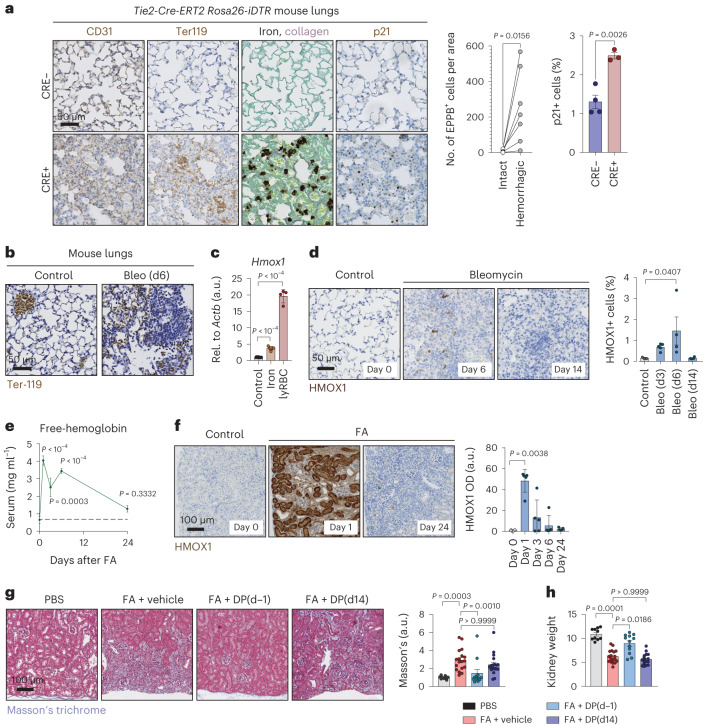
Vascular insults provoke iron accumulation. **a**, Histochemistry for iron and collagen, by combining EPPB and SRFG staining, and immunohistochemistry for CD31, Ter-119 and p21. Representative images of mouse lung sections from *Tie2-Cre-ERT2*^−^ (*n* = 4) and *Tie2-Cre-ERT2*^*+*^ (*n* = 3) *Rosa26-iDTR* mice after 2 weeks of treatment with tamoxifen and diphtheria toxin (left). Scale bar, 50 μm. Number of EPPB-positive cells per unit area in foci with or without microhemorrhages (middle). We used the Wilcoxon matched-pairs signed-rank test. Percentage of p21^+^ cells in *Tie2-Cre-ERT2*^−^ (*n* = 4) and *Tie2-Cre-ERT2*^*+*^ (*n* = 3) *Rosa26-DTR* mice. **b**, Immunohistochemistry for Ter-119. Representative images of mouse lung sections 6 d post-PBS (control) or bleomycin (1.5 U kg^−1^ body weight). Scale bar, 50 μm. **c**, mRNA levels of *Hmox1* relative to *Actb* in MEFs after 3 d of culture in control conditions (*n* = 10), or in the presence of iron (660 μM; *n* = 6) or lysed RBCs (25-fold dilution; *n* = 4). Values represented are normalized to the average of the controls (a.u.). **d**, Immunohistochemistry for HMOX1. Representative images of mouse lung sections 0 d (control; *n* = 3), 6 d (*n* = 4), 14 d (*n* = 4) post-bleomycin (1.5 U kg^−1^ body weight) and quantification. Scale bar, 50 μm. **e**, Free-hemoglobin levels, measured by ELISA, in the sera of mice 0 d (*n* = 7), 1 d (*n* = 5), 3 d (*n* = 5), 6 d (*n* = 5) and 24 d (*n* = 5) post-FA. **f**, Immunohistochemistry for HMOX1. Representative images of mouse kidney sections 0 d (*n* = 4), 1 d (*n* = 5), 3 d, 6 d and 24 d (*n* = 5) post-FA and quantification of optical density (OD). Scale bar, 100 μm for all images. **g**,**h**, Analysis of kidneys from control mice (PBS injected; *n* = 10) and FA-treated mice, which either received vehicle (*n* = 18), daily deferiprone starting 1 d before (DP(d − 1); *n* = 16) or 14 d after (DP(d14); *n* = 18) disease induction by FA. Mice were analyzed 28 d post-FA. **g**, Representative images of Masson’s trichrome staining and quantification. Scale bar, 100 μm. **h**, Kidney weight relative to tibia length. When bar graphs are shown, they represent mean ± s.e.m.; dots represent individual mice; all experiments were performed at least three times with similar results. Unless stated otherwise, we compared distributions using a two-tailed unpaired *t*-test or, for multiple comparisons, we used a one-way ANOVA (**c**–**e**) or a Kruskal–Wallis test (**f**–**h**). Source data

**Extended Data Fig. 3:**
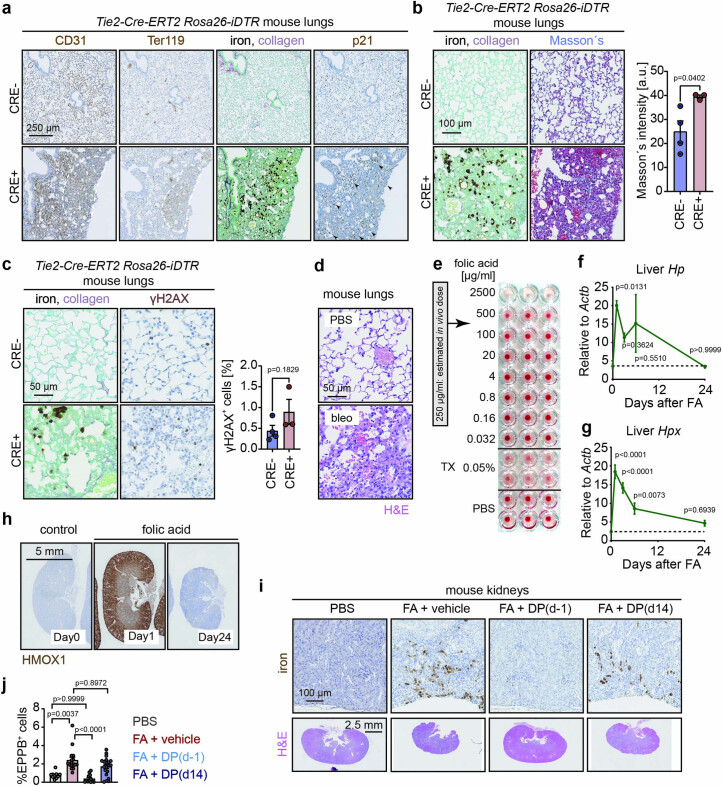
Vascular insults provoke iron accumulation. **a-c**, Histological analysis of mouse lung sections from *Tie2-Cre-ERT2*^*-*^ and *Tie2-Cre-ERT2*^*+*^ *Rosa26-iDTR* mice after two weeks of treatment with tamoxifen and diphtheria toxin. **a**, Histochemistry for iron and collagen by Perl’s Prussian blue (PPB) and Sirius Red/Fast Green (SRFG) staining, and immunohistochemistry for CD31, Ter-119, and p21. Dashed line indicates area with reduced CD31 expression associated with accumulation of microhemorrhagic RBCs. Scale bar, 250 μm. **b**, Histochemistry for iron and collagen by enhanced PPB and SRFG staining, and by Masson’s Trichrome staining. Representative images to the left. Scale bar, 100 μm. Quantification of Masson’s Trichrome intensity in *Tie2-Cre-ERT2*^*-*^ (n = 4) and *Tie2-Cre-ERT2*^*+*^ (n = 3) *Rosa26-iDTR* mice to the right. **c**, Histochemistry for iron and collagen, by enhanced PPB and SRFG staining, and immunohistochemistry for γH2AX. Representative images and quantification of percentage of γH2AX cells in lung sections of in *Tie2-Cre-ERT2*^*-*^ (n = 4) and *Tie2-Cre-ERT2*^*+*^ (n = 3) *Rosa26-iDTR* mice. Scale bar, 50 μm. **d**, Representative images of H&E staining of mouse lungs 6 days post-PBS or bleomycin (1.5 U/kg of body weight). Scale bar, 50 μm. **e**, Ex vivo RBC hemolysis assay with different doses of folic acid, with triton X-100 (TX) and PBS. **f-g**, mRNA levels of **f**, haptoglobin (*Hp*) and **g**, hemopexin (*Hpx*) relative to *Actb* in the livers of mice from before (n = 6), and 1 day (n = 5), 3 days (n = 5), 6 days (n = 5), or 24 days (n = 5) post-folic acid. We used one-way ANOVA. **h**, Immunohistochemistry for HMOX1. Representative images of mouse kidney sections 0 days, 1 day, 3 days, and 24 days post-folic acid. Scale bar, 5 mm. **i**, Histochemical staining for iron by enhanced PPB, and H&E staining of kidneys from control mice (PBS injected; n = 10), and mice in which kidney fibrosis was induced by folic acid. Mice in the folic acid group either received vehicle (n = 18) or deferiprone injected daily starting 1 day before (DP(d-1); n = 16) or 14 days after (DP(d14); n = 18) disease induction by folic acid. Mice were analyzed on 28 post-FA. Scale bar, 100 μm for upper panels, and 2.5 mm for lower panels. **j**, Quantification of enhanced PPB positive cells of kidneys from panel j. Where it is not stated otherwise, bar graphs represent mean ± SEM; individual values for each mouse is represented as points; experiments were at least performed three times with similar results; unless stated otherwise, we compared distributions using two-tailed unpaired t-test; for multiple-comparisons, we used the Kruskal–Wallis test. Source data

Considering the capacity of iron to induce fibrosis, we wondered whether vascular or hemolytic injuries could cause iron release from damaged RBCs and thereby inflammation, senescence and fibrosis. To directly interrogate this, we generated a mouse model (*Tie2-Cre-ERT2; Rosa26-iDTR*) in which a fraction of endothelial cells can be ablated by the injection of tamoxifen and diphtheria toxin leading to microhemorrhages, mainly in the lungs, and compatible with mouse survival. Sites of microvascular injuries were visible as regions with abnormal CD31 staining and extravascular Ter-119^+^ RBCs (Fig. 3a and Extended Data Fig. 3a). Of note, these regions contained abundant iron-loaded cells and p21^+^ cells (Fig. 3a and Extended Data Fig. 3a) and were marked by increased intensity of Masson’s trichrome staining (Fig. 3a and Extended Data Fig. 3b) that highlights collagens in blue. In the case of γH2AX, the observed increment was non-significant (Extended Data Fig. 3c). Overall, these findings suggest a causative role of vascular injury in iron accumulation and senescence in vivo.

We wondered whether vascular or hemolytic injuries are involved in the pathobiology of FA-induced kidney injury and bleomycin-induced lung injury. In the case of bleomycin, we found evidence for microhemorrhagic injuries in the lungs 6 days post-bleomycin, as indicated by foci with accumulating extravascular Ter-119^+^ RBCs (Fig. 3b). Bleomycin-induced microhemorrhages were also visible in hematoxylin and eosin (H&E) staining (Extended Data Fig. 3d). We tested the effects of these microhemorrhagic injuries by analyzing the levels of heme oxygenase 1 (*Hmox1*), a gene that we found to be highly responsive to damaged RBCs in vitro (Fig. 3c). Numbers of HMOX1^+^ cells peaked on day 6 post-bleomycin and levels normalized by day 14 post-injury (Fig. 3d). In the case of FA, we found that this compound is hemolytic when applied to isolated RBCs in the concentration range used to provoke kidney injury (Extended Data Fig. 3e). In vivo, FA-induced hemolysis was reflected by a rapid spike in serum levels of free hemoglobin (Fig. 3e) and in hepatic mRNA levels of haptoglobin (*Hp*, hemoglobin scavenger) and hemopexin (*Hpx*, heme scavenger) (Extended Data Fig. 3f,g), reaching their maximum as early as 1 d post-injury, which was followed by a progressive decline. In line with these findings, FA induced a transient and robust increase in the levels of heme oxygenase 1 (HMOX1) in the kidneys 1 d post-injury (Fig. 3f and Extended Data Fig. 3h), possibly in response to the hemolytic filtrate. Of note, all the parameters measuring RBC damage were consistent with a rapid but transient release of hemolytic iron, which is in line with the kinetics of hepatic hepcidin (see above; Fig. 1i,j).

Finally, to test the causal role of iron in FA-induced renal fibrosis, we treated mice with FA to provoke kidney injury in the presence or absence of the iron chelator deferiprone (DP). In one group of mice, labeled DP(d − 1), DP was administered starting 1 d before FA and continued until analysis at day 28 post-FA. In another group of mice, DP(d14), treatment was initiated at day 14 post-FA until day 28. Notably, mice in the DP(d − 1) group were largely protected from FA-induced kidney fibrosis and atrophy (Fig. 3g,h), and this was associated with iron levels comparable to those seen in the kidneys of control mice (Extended Data Fig. 3i,j); however, the regimen of DP that we have been using was not effective in reversing already established fibrotic scars (Fig. 3g,h). We wondered whether this could be because the already accumulated iron in damaged tubular cells was not accessible to DP. Indeed, we have found that DP applied 14 d after fibrosis induction was incapable of removing the iron already accumulated in fibrotic kidneys (Extended Data Fig. 3i,j). We speculate that the long-term form of iron accumulation in fibrosis is inaccessible to DP. Together, these observations further reinforce the link between the presence of iron and the development of fibrosis. We conclude that vascular injuries and hemolytic RBCs have the capacity to drive iron accumulation and may contribute to the initiation of senescence and fibrosis in some pathologies.

### Iron and lysed erythrocytes cause cellular senescence in vitro

**Fig. 4:**
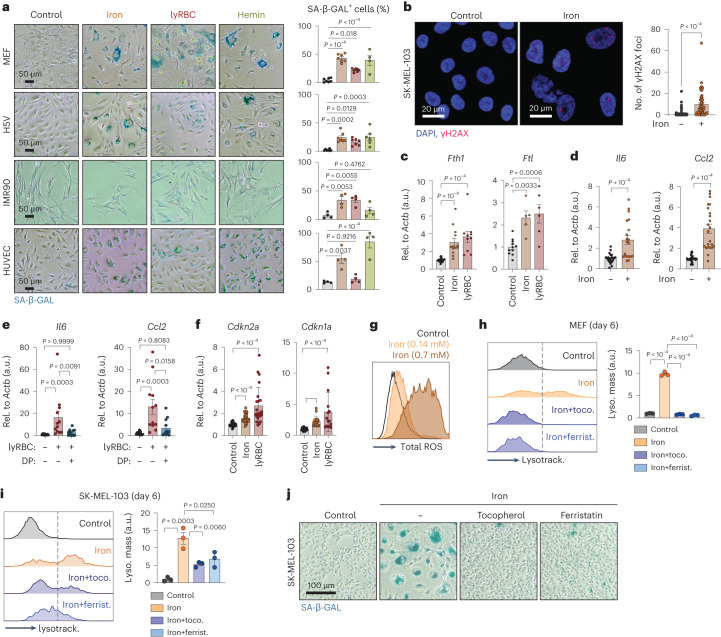
Iron and lysed erythrocytes cause cellular senescence in vitro. **a**, SA-β-GAL staining of H5V cells, IMR90 fibroblasts, HUVEC cells and MEFs 14 d after culture in control conditions (control; *n*_MEF_ = 7; *n*_H5V_ = 7; *n*_IMR90_ = 4; *n*_HUVEC_ = 4) or in the presence of iron (660 μM; *n*_MEF_ = 7; *n*_H5V_ = 7; *n*_IMR90_ = 4; *n*_HUVEC_ = 4), lysed RBCs (25-fold dilution; *n*_MEF_ = 7; *n*_H5V_ = 7; *n*_IMR90_ = 4; *n*_HUVEC_ = 4) or hemin (10 μM; *n*_MEF_ = 4; *n*_H5V_ = 6; *n*_IMR90_ = 4; *n*_HUVEC_ = 4). Representative images and quantification. Scale bar, 50 μm. **b**, Immunofluorescent staining for *γ*H2AX in SK-MEL-103 cells cultured with vehicle control (*n* = 44) or iron (660 μM; *n* = 51) for 2 d. Scale bar, 20 μm. Representative images and quantification of *γ*H2AX foci per nuclei. Nuclei were stained with DAPI. **c**, mRNA levels of *Fth1* (*n*_control_ = 23; *n*_iron_ = 13; *n*_lyRBC_ = 10) and *Ftl* (*n*_control_ = 11; *n*_iron_ = 5; *n*_lyRBC_ = 6) relative to *Actb* in MEFs, which were cultured for 3 d in the presence of vehicle (control), iron (660 μM) or lysed RBCs (25-fold dilution). **d**, mRNA levels of *Il6* (*n*_control_=22; *n*_iron_ = 20) and *Ccl2* (*n*_control_ = 2; *n*_iron_ = 23) relative to *Actb* in MEFs, which were cultured for 3 d with vehicle (control) or iron (660 μM). **e**, mRNA levels of *Il6* (*n*_control_ = 12; *n*_lyRBC_ = 12; *n*_lyRBC+DP_ = 12) and *Ccl2* (*n*_control_ = 12; *n*_lyRBC_ = 12; *n*_lyRBC+DP_ = 12) relative to *Actb* in MEFs, which were cultured for 3 d with vehicle (control) or lysed RBCs (25-fold dilution) with or without DP (1 mM). **f**, mRNA levels of *Cdkn2a* (*n*_control_ = 30; *n*_iron_ = 31; *n*_lyRBC_ = 23)*, Cdkn1a* (*n*_control_ = 26; *n*_iron_ = 26; *n*_lyRBC_ = 21) relative to *Actb* in MEFs, which were cultured for 7 d with vehicle (control), iron (660 μM) or lysed RBCs (25-fold dilution). **g**, Flow-cytometric analysis of total ROS levels in MEFs after 7 d with vehicle or iron (140 μM or 660 μM). **h**,**i**, Flow-cytometric analysis of lysosomal mass by LysoTracker staining of MEFs (**h**) and SK-MEL-103 cells (**i**) after culturing cells for 7 d with vehicle (*n*_MEF_ = 3; *n*_SKMEL_ = 3) or in the presence of iron (660 μM; *n*_MEF_ = 3; *n*_SKMEL_ = 3) with or without the ROS scavengers tocopherol (50 μM; *n*_MEF_ = 3; *n*_SKMEL_ = 3) and ferristatin (1 μM; *n*_MEF_ = 3; *n*_SKMEL_ = 3). Representative histograms and quantification of lysosomal mass. **j**, SA-β-GAL staining of SK-MEL-103 after 14 d in culture in control conditions or in the presence of iron (140 μM) with or without tocopherol (50 μM) or ferristatin (1 μM). Scale bar, 100 μM. When bar graphs are shown, they represent mean ± s.e.m.; dots represent experimental replicates; experiments were performed at least three times with similar results. Data were analyzed by a Mann–Whitney *U*-test (**b**); two-tailed unpaired *t*-test (**d**); one-way ANOVA for multiple comparisons (**a**,**c**,**h**,**i**) and a Kruskal–Wallis test (**e**,**f**). Source data

**Extended Data Fig. 4:**
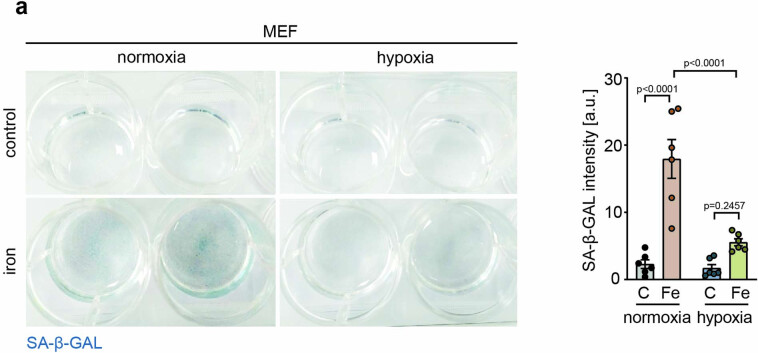
Iron and lysed erythrocytes cause cellular senescence *in vitro*. **a**, Representative images of cell culture plate wells confluent with MEFs that were cultured in control conditions or in the presence of iron (660 μM) in normoxia or hypoxia, and subsequently stained for SA-β-GAL (n = 6 for each condition). Quantification of SA-β-GAL intensity in wells to the right. Bar graphs represent mean ± SEM; we used one-way ANOVA. Source data

To gain mechanistic insight into the connection between iron and senescence, we explored the effects of iron on in vitro cultured cells. We found in pilot experiments that for iron uptake to occur, cells had to be primed by culturing them in low transferrin-bound iron conditions (achieved by reducing the serum to 0.5%). Under these conditions, addition of iron, lysed RBCs or hemin (a derivative of the iron-bound cofactor of hemoglobin), efficiently triggered senescence (measured by SA-β-GAL) in all the cell lines tested, namely, primary mouse embryo fibroblasts (MEFs), immortalized mouse endothelial cells (H5V), non-immortalized human lung fibroblasts (IMR90) and non-immortalized human umbilical vein endothelial cells (HUVECs) (Fig. 4a). Senescence induced by iron was also characterized by nuclear γH2AX foci (Fig. 4b), tested in human melanoma SK-MEL-103 cells. Further analyses of senescence induced by iron and lysed RBCs were performed in MEFs. Three days post-treatment initiation, we observed increased transcript levels of the ferritin subunits (*Fth1* and *Ftl*) (Fig. 4c), together with SASP factors (*Il6* and *Ccl2*) (Fig. 4d,e). In the case of lysed RBCs, these transcriptional effects were ablated by the presence of DP (Fig. 4e), indicating that iron is the key mediator. Induction of senescence markers of cell-cycle arrest *Cdkn2a* (p16) and *Cdkn1a* (p21) (Fig. 4f) were detected 7 d post-treatment. Iron also caused a dramatic elevation in cellular ROS levels (Fig. 4g) and cells presented with an expansion in their lysosomal mass, as measured by LysoTracker staining (Fig. 4h), both tested 6–7 d post-treatment initiation. Notably, the ROS scavengers tocopherol and ferristatin potently mitigated lysosomal expansion in response to iron (Fig. 4h). Similar observations were made in human melanoma cells SK-MEL-103 (Fig. 4i). ROS scavengers also prevented the emergence of large SA-β-GAL-positive cells (Fig. 4j) and so was hypoxia (Extended Data Fig. 4a). We conclude that iron, in its free form or when released from damaged RBCs, is a potent trigger of ROS-mediated cellular senescence in vitro.

### Iron accumulation in senescent cells drives the SASP

**Fig. 5:**
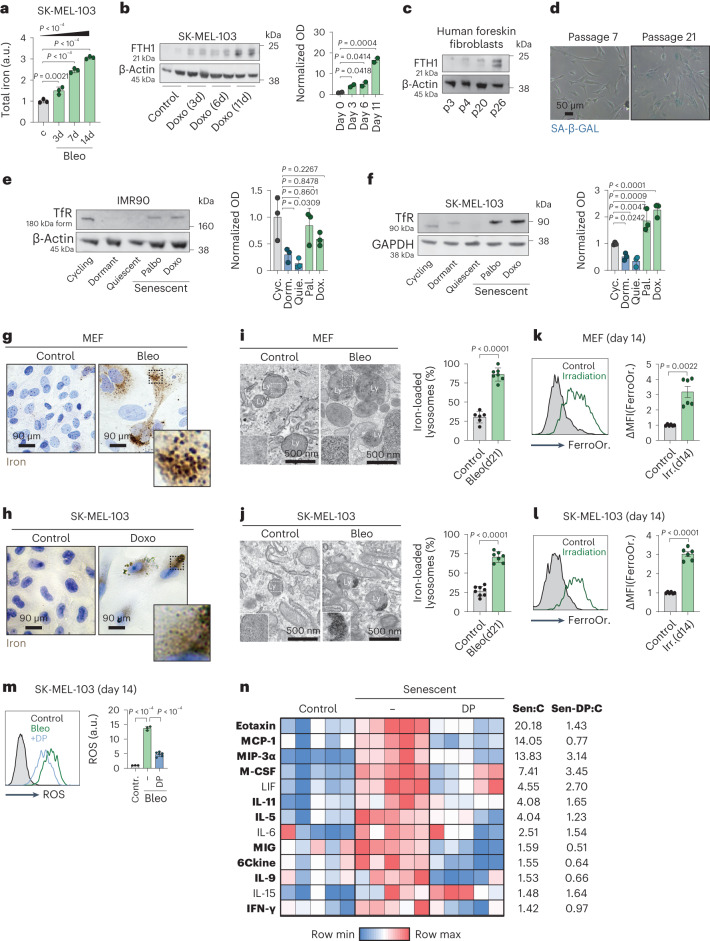
Iron accumulation in senescent cells drives the SASP. **a**, Total iron levels in SK-MEL-103 cells before (*n* = 3) and 3 d (*n* = 3), 7 d (*n* = 3) and 14 d (*n* = 3) post-damage by bleomycin. **b**, Ferritin heavy chain (FTH1) levels in SK-MEL-103 cells before (*n* = 2) and 3 d (*n* = 2), 6 d (*n* = 2) and 11 d (*n* = 2) post-doxorubicin (Doxo) (100 nM). Densitometric quantification (right). **c**, FTH1 levels in human foreskin fibroblasts undergoing replicative senescence with passaging. Passage numbers (p). **d**, Representative images of SA-β-GAL staining of human primary foreskin fibroblasts at passage 7 and at passage 21. Scale bar, 50 μm. **e**,**f**, TfR levels in cycling, dormant (INK128; 200 nM for 7 d), quiescent (serum starvation for 7 d) and senescent (induced by doxorubicin (100 nM) or palbociclib (1 µM)) IMR90 human fibroblasts (**e**) and SK-MEL-103 human melanoma cells (**f**) (*n* = 3 for each condition). Densitometric quantification (right). **g**,**h**, EPPB staining of MEFs (**g**) and SK-MEL-103 cells (**h**), controls and 21 d post-bleomycin (10 mU ml^−1^) or post-doxorubicin (100 nM). Insets show punctate accumulation of iron. **i**,**j**, Transmission electron microscopical images of MEFs (**i**) and SK-MEL-103 cells (**j**), control (*n*_MEF_ = 6; *n*_SKMEL-103_ = 8) and 21 d post-bleomycin (10 mU ml^−1^) (*n*_MEF _= 7; *n*_SKMEL-103_ = 7). Insets show accumulation of electron-dense material in the lysosomes compatible in size and organization with ferritin-bound iron. Ly, lysosomes; M, mitochondria. Quantification of iron-loaded lysosomes (right). **k**,**l**, Levels of labile iron (Fe^2+^) in control and senescent (irradiation-induced) MEFs (**k**) and SK-MEL-103 cells (**l**), measured by flow cytometry using the dye FerroOrange (*n* = 6 for each condition). Representative histograms and quantification. **m**, Levels of total ROS in control (*n* = 3) and senescent (bleomycin-induced; 10 mU ml^−1^) SK-MEL-103 cells, which received vehicle (*n* = 3) or DP (200 μM; *n* = 6) 30 min before measurement. Representative histograms and quantification. **n**, Heat map showing the protein levels of SASP factors in control MEF (*n* = 5) and irradiated senescent MEFs (*n* = 5) cultured for 3 d with vehicle or DP (100 μM; *n* = 5). SASP factors in bold were suppressed by DP. Fold change relative to the controls for senescent MEFs (Sen:C), and for senescent MEFs treated with deferiprone (Sen-DP:C). When bar graphs are shown, they represent mean ± s.e.m. Dots represent experimental replicates. Experiments were performed at least three times with similar results; we used a two-tailed unpaired *t*-test, (**j**,**k**,**m**) and a Mann–Whitney *U*-test (**l**); for multiple comparisons we used one-way ANOVA; we analyzed for linear trends between the column means from left to right (**a**). Source data

**Extended Data Fig. 5:**
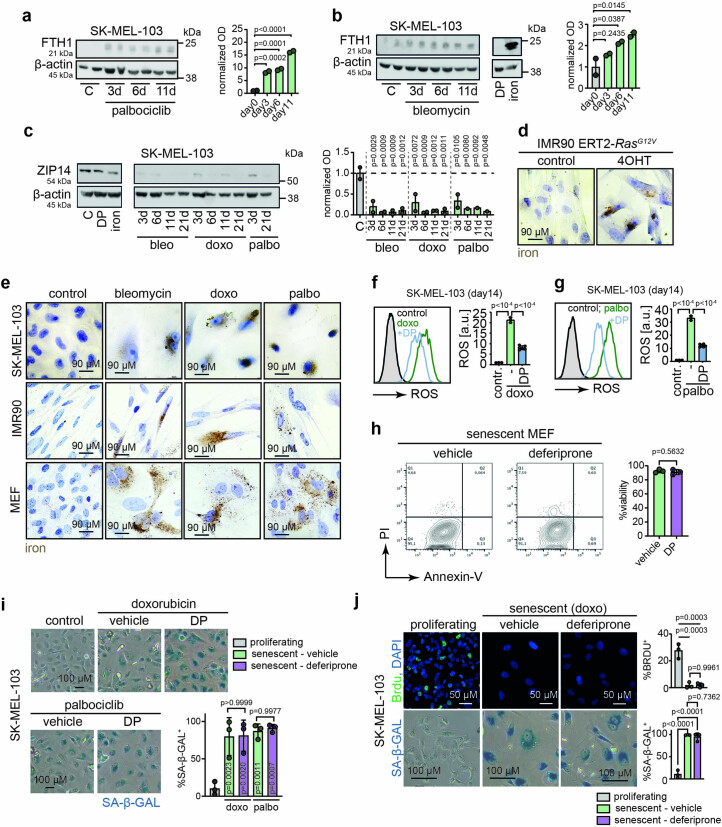
Iron accumulation in senescent cells drives the SASP. **a-b**, FTH1 levels in SK-MEL-103 cells before and 3, 6, and 11 days post-damage (n = 2 for each condition) by **a**, palbociclib (1 µM) and **b**, bleomycin (10mU/ml), and after 3 days of culture in the presence of deferiprone (DP; 100 μM) or iron (140 μM). Densitometric analysis to the right. **c**, ZIP14 levels in SK-MEL-103 cells before (n = 2) and 3 days after culture in the presence of iron (140 μM) or deferiprone (DP; 100 μM), or 3, 6, 11 and 21 days post-damage by bleomycin (10mU/ml; n = 2 for each condition), doxorubicin (100 nM; n = 2 for each condition) or palbociclib (1 µM; n_day3_ = 2; n = 1 for all other conditions). Representative images and densitometric analysis. **d-e**, Enhanced Perl’s Prussian blue staining of SK-MEL-103, IMR90, and MEF control or senescent cells. Senescence was induced by **d**, overexpression of mutant Ras^G12V^ in IMR90 cells, or by **e**, bleomycin (10mU/ml), doxorubicin (100 nM) or palbociclib (1 µM) followed by culture for 21 days. **f-g**, Total ROS levels measured by flow cytometry in control (n_doxo_=3; n_palbo_ = 3) and senescent SK-MEL-103 cells, which received 30 minutes prior to the measurement vehicle (n_doxo_=3; n_palbo_ = 3) or deferiprone (DP; 200 μM; n_doxo_ = 6; n_palbo_ = 6). Senescence was induced by **f**, doxorubicin (100 nM), or **g**, palbociclib (1 µM). Representative histograms and quantification. **h**, Representative plots and quantification of cell viability analyzed by flow cytometry by annexin-V and propidium iodide (PI) staining in senescent MEFs cultured with deferiprone (200 µM; n = 3) or vehicle (water; n = 3) for 4 days. **i**, Representative images and quantification of SA- β-GAL staining in control (untreated) and senescent (doxorubicin, c = 100 nM; or Palbociclib, c = 1 µM) SK-MEL-103 cells. Senescence was induced in the presence of the iron chelator deferiprone (DP; c = 200 μM) for 4 days followed by 3 additional days of culture (n = 3 for each condition). Scale bar, 100 μm. **j**, Representative images and quantification of BrdU staining, and SA-β-GAL staining of control and senescent (doxorubicin; c = 100 nM) SK-MEL-103 cells. 7 days post-senescence induction, cells were treated with the iron chelator deferiprone (DP; 100 μM) or vehicle (water) for 4 days. Scale bar, 50 μm (upper panel), and 100 μm (lower panel), (n = 3 for each condition). Where it is not stated otherwise, bar graphs represent mean ± SEM; experimental replicates are represented as points; experiments were at least performed three times with similar results; we compared distributions using two-tailed unpaired t-test; for multiple-comparisons, we used one-way ANOVA. Source data

We wanted to understand how cellular damage and senescence, triggered in the absence of excess iron, may lead to iron accumulation in vitro^33,34^. For this, we first investigated the kinetics of total iron levels after cellular damage (bleomycin, doxorubicin, irradiation and palbociclib) under normal culture conditions, without priming cells in low-serum conditions. We observed a progressive increase in total iron levels and upregulation of ferritins as early as 3 d post-insult (Fig. 5a,b and Extended Data Fig. 5a,b), suggesting that iron accumulation starts before the full development of senescence. Upregulation of ferritin also accompanied replicative senescence in human foreskin fibroblasts (Fig. 5c,d). We wondered what drives iron accumulation in senescent cells and investigated the expression of canonical and non-canonical iron transporters. Notably, we observed that senescent cells express high levels of the transferrin receptor (TfR), similar to those in proliferative cells, whereas quiescent cells (by serum deprivation or by mTOR inhibition ‘dormant’) profoundly reduced the levels of TfR (Fig. 5e,f). Contrary to TfR, the expression of the non-canonical iron transporter ZIP14 (SLC39a14) diminished within 3 d post-damage (Extended Data Fig. 5c). We propose that TfR levels in senescent cells maintain an iron influx similar to the one in cycling cells; however, a critical difference between these two cell states is that proliferating cells split their intracellular iron content between daughter cells with every proliferation cycle, whereas senescent cells accumulate iron with no possibility of splitting it by cell division.

Using enhanced Perl’s Prussian blue staining, we found that iron deposits concentrated in cytoplasmic puncta in senescent cells (Fig. 5g,h and Extended Data Fig. 5d,e). Transmission electron microscopy (TEM) suggested that these puncta correspond to lysosomes containing abundant ferritin-bound iron (Fig. 5i,j). In addition to increased ferritin-bound iron (inert Fe^3+^), we also found that the labile iron (oxidative Fe^2+^) pool was significantly elevated in senescent cells (Fig. 5k,l). We wondered whether the high ROS levels characteristic of senescent cells^49,50^ are fueled, at least in part, by labile iron. Of note, when fully senescent cells were treated for 30 min with DP, ROS levels were significantly reduced (Fig. 5m and Extended Data Fig. 5f,g). ROS is known to be a main trigger of the secretory phenotype of senescent cells (SASP)^51,52^ and therefore, we asked whether iron chelation would also interfere with the SASP. Control and fully senescent cells were tested for the intracellular protein levels of a total of 44 cytokines and chemokines, out of which, 13 were significantly elevated upon senescence (Fig. 5n). Notably, treating senescent cells with a non-toxic dose (Extended Data Fig. 5h) of DP for 3 d ablated the large majority of cytokines and chemokines in the SASP (10 out of 13 were significantly suppressed) (Fig. 5n). Not all hallmarks of cellular senescence are due to iron accumulation, as iron chelation by DP did not prevent cell-cycle arrest or SA-β-GAL staining when coapplied with senescence inducers (Extended Data Fig. 5i), neither did it revert already established senescent cells into proliferating cells (Extended Data Fig. 5j).

We conclude that cellular damage, even when triggered under normal iron conditions, results in a progressive iron accumulation that causes the characteristic high levels of ROS and the SASP of senescent cells.

### Single-cell dynamics of iron accumulation in lung fibrosis

**Fig. 6:**
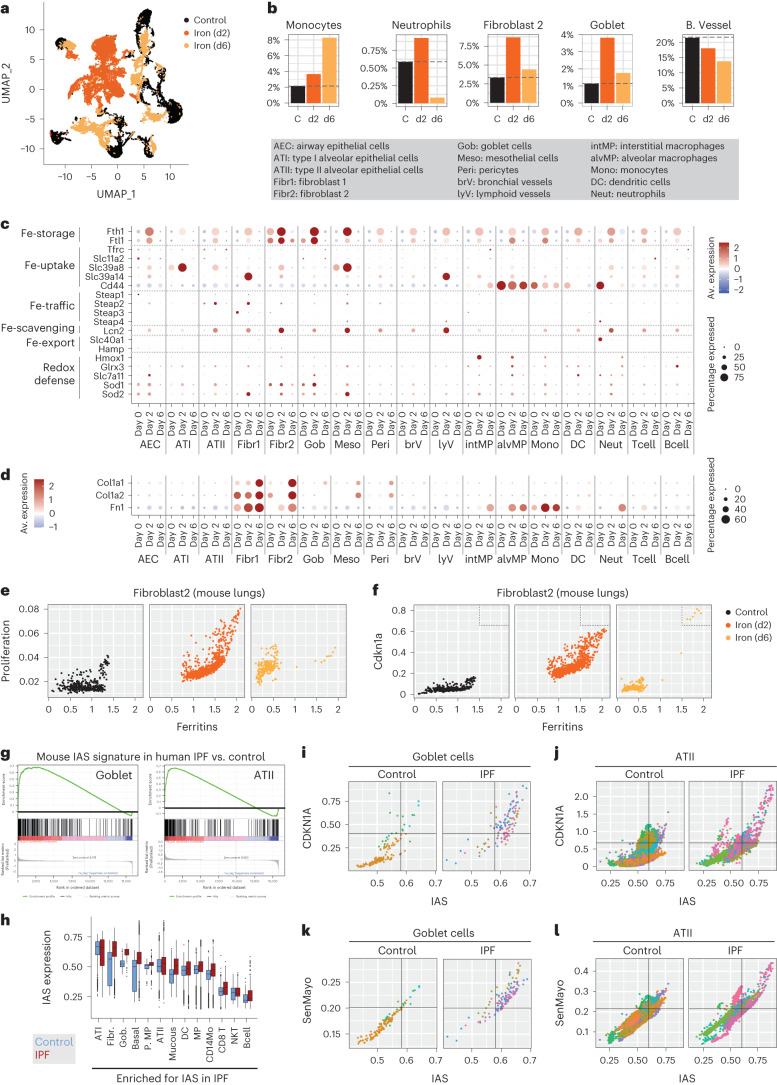
Single-cell dynamics of iron accumulation in lung fibrosis. **a**–**f**, snRNA-seq analysis of cells from lungs of control mice receiving intratracheal administration of PBS (6 d; *n* = 1) and mice receiving iron intratracheally (500 nM) 2 d (*n* = 1) or 6 d (*n* = 1) before analysis. UMAP visualization of cells (**a**). Percentages of cell types with robust change in abundance in response to iron (**b**). Average expression and percentage of cells expressing genes regulating iron homeostasis (**c**), type I collagens (**d**) and fibronectin in individual cell types. Dot plot showing the scaled average expression of an individual gene across all cell types and conditions. Percentage expressed shows the percentage of cells that showed detectable expression of the analyzed gene within a specific cell type at a specific condition. Scatter-plots showing the expression of a cell proliferation signature (**e**) and *Cdkn1a* (**f**) with respect to ferritin expression in fibroblast 2 cells from control lung and lungs 2 d or 6 d post-iron. **g**–**l**, Analysis of human lung scRNA-seq meta-analysis from controls and patients with IPF. Data were generated by analyzing a meta-analysis of human single-cell lung transcriptome that compared 26 control individuals to 19 patients with IPF. Enrichment plot of the gene set enrichment analysis of the IAS in goblet cells and type II alveolar epithelial cells (ATII) in a human lung scRNA-seq meta-analysis comparing control (*n* = 26) individuals to patients with IPF (*n* = 19) (**g**). Expression of the mouse IAS in cell types which show a significant enrichment of IAS genes in the comparison between control and IPF lung cells in a human scRNA-seq meta-analysis (**h**). Boxes represent the median and 25th and 75th percentiles of the data. The whiskers extend to the largest upper and lower values no further than 1.5 × interquartile range. Outlier points are plotted individually. Scatter-plots showing the expression of *CDKN1A* (**i**,**j**) and the SenMayo senescence signature (**k**,**l**) with respect to the expression of the IAS in goblet cells (**i**,**k**) and in type II alveolar epithelial cells (ATII) (**j**,**l**) in a human lung scRNA-seq meta-analysis from control individuals (*n* = 5 for goblet cells; *n* = 25 for ATII) and patients with IPF (*n* = 6 for goblet cells: *n* = 19 for ATII). Each color represents cells from an individual donor.

**Extended Data Fig. 6:**
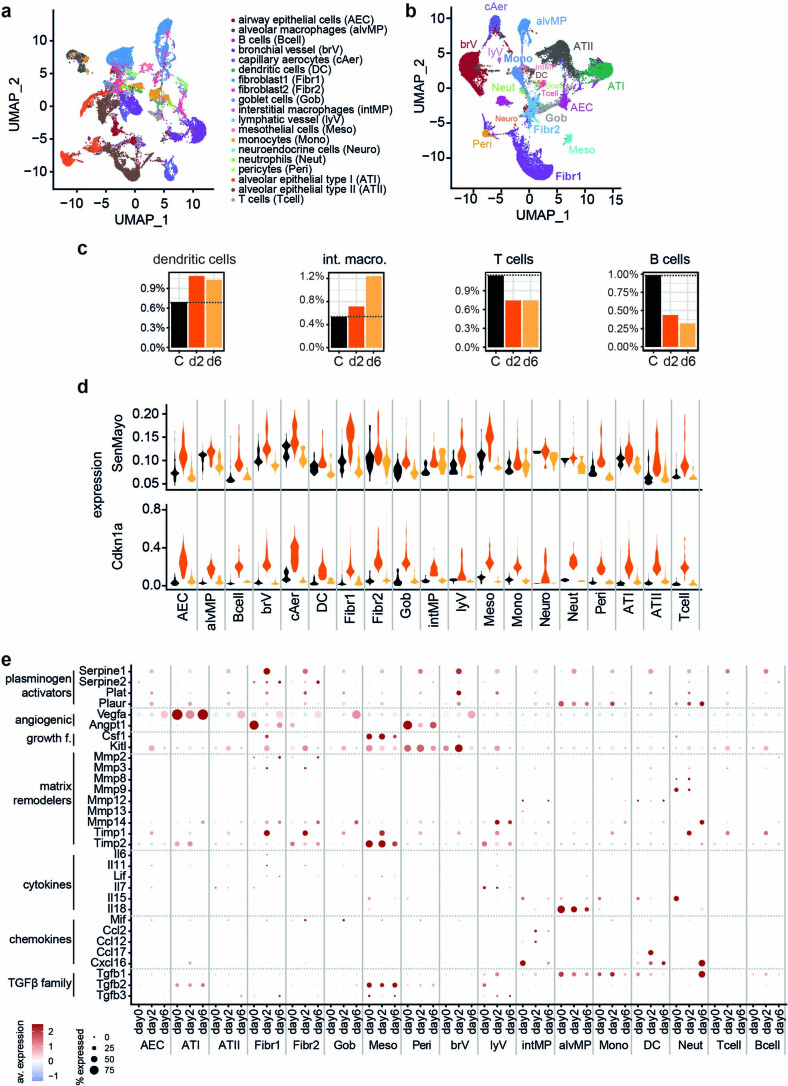
Single-cell dynamics of iron accumulation in lung fibrosis. **a-e**, snRNA-seq analysis of cells from lungs of control mouse receiving intratracheal administration of PBS (6 days; n = 1), and mice receiving intratracheal iron (500 nM) 2 days (n = 1) or 6 days (n = 1) prior to analysis. **a**, UMAP visualization of the merged dataset. Cell types are distinguished by color. **b**, UMAP visualization of cells after using an anchors-based integration. Cell types are distinguished by color. **c**, Percentages of cell types that changed in abundance in response to intratracheal iron. **d**, Violin plots showing changes in the expression of Cdkn1a (p21), and the SenMayo senescence signature in individual cell types in response to intratracheal iron delivery. **e**, Expression of prominent secreted factors involved in fibrogenesis, and associated with inflammation, chemoattraction, and tissue remodeling across cell types and conditions. Dot plot showing the average scaled expression of an individual gene across all cells and conditions. % expressed shows the percentage of cells that showed detectable expression of the analyzed gene within a specific cell type at a specific condition. Source data

**Extended Data Fig. 7:**
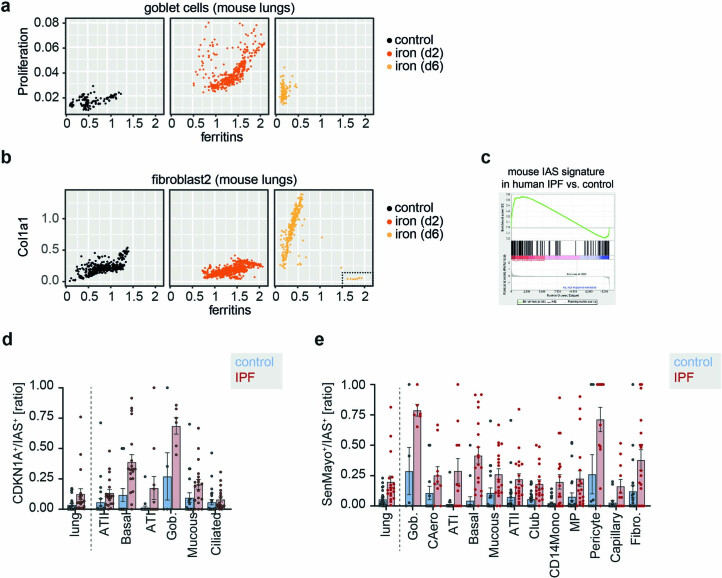
Single-cell dynamics of iron accumulation in lung fibrosis. **a-b**, Scatter plots showing the expression of **a**, a cell proliferation signature in goblet cells and **b**, Col1a1 in fibroblast 2 cells from control lung, and mouse lungs 2 days or 6 days post-iron with respect to ferritin expression. **c-e**, Analysis of human lung scRNA-seq meta-analysis from control patients and patients with IPF. **c**, GSEA of the IAS in the comparison between control and IPF lung tissues. **d**-**e**, ratio of cells positive both for the IAS and **d**, for CDKN1A or **e**, the SenMayo senescence signature in total lung cells and in individual cell types. Highlighted cell types presented with double positive cells significantly more frequently in IPF donors (n = 19) than control donors (n = 26). Bar plots represent mean ± SEM of the percentage of double positive cells. Each dot corresponds to a donor.

We were intrigued by the different kinetics of injury-induced iron release (transient and short term) and injury-induced iron accumulation (slowly progressive and long term). Specifically, tissue injuries provoked a transient burst in hemolysis markers (detectable by HMOX1 and by hepatic *Hamp*, *Hp* and *Hpx*), whereas iron accumulation in the fibrotic lesions was progressive and continuous even after indicators of hemolysis subdued to basal levels. To get deeper insight into the cell types underlying these two phases of the response to iron, we performed single-cell RNA sequencing (scRNA-seq) of mouse lungs at 2 days and 6 days post-iron exposure. Our strategy was to first determine a signature of iron accumulation in vivo and then use this signature to interrogate publicly available datasets of human patients with IPF. Specifically, we performed single-nuclei RNA sequencing (snRNA-seq) of lungs from mice that received a single intratracheal dose of iron at 2 days or 6 days post-treatment (together with phosphate-buffered saline (PBS)-treated control). Iron administration provoked dramatic changes in the lung, clearly implied by the dimensionality reduction map created by using a Uniform Manifold Approximation and Projection (UMAP) (Fig. 6a). Nevertheless, cell types clustered separately within each condition (Extended Data Fig. 6a) and using an anchors-based integration, cells of the same type from different conditions clustered together (Extended Data Fig. 6b). For subsequent analysis, we used the merged dataset without anchors. Next, we investigated changes in the relative cellular composition of mouse lungs in response to iron. snRNA-seq analysis showed that iron provoked the transient recruitment of neutrophils, a sustained expansion of monocytes, interstitial macrophages and dendritic cells, and progressively suppressed the relative abundance of bronchial vessel cells (Fig. 6b and Extended Data Fig. 6c). All these observations are in accordance with our earlier findings acquired by flow cytometry and histology (Fig. 2b–d and Extended Data Fig. 2a–c). In the non-immune compartment, we observed a pronounced, transient increase in the relative abundance of goblet cells and a population of fibroblasts (named ‘fibroblast 2’), which clustered separately from other fibroblasts (‘fibroblast 1’) (Fig. 6b). Next, we investigated which cell types took up iron by assessing changes in the expression of prominent iron homeostasis genes. Levels of ferritins, the iron sequestering protein lipocalin 2 and superoxide dismutases (SOD1/2) were increased in all cell types 2 d post-iron, suggesting that a fraction of cells in each cell type was directly exposed to iron; however, three cell types showed particularly high induction in these genes, namely, fibroblast 2 cells, goblet cells and mesothelial cells (Fig. 6c). Other genes involved in cellular iron homeostasis showed cell type-dependent changes (Fig. 6c). Iron also induced the expression of senescence-related genes (determined using the SenMayo signature^53^), promoted the expression of *Cdkn1a* in all cell types (Extended Data Fig. 6d) and induced the transcription of various secreted factors involved in fibrogenesis (Extended Data Fig. 6e). The most dominant changes in secreted factors were the induction of plasminogen activators, in particular *Serpine1*, the induction of a regulator of extracellular matrix turnover, *Timp1*, both of which are well known drivers of fibrogenesis^44,54^ and suppression of angiogenic factors, angiopoietin 1 (*Angpt1*) and *Vegfa*, believed to be a main contributor to fibrosis-associated vascular rarefaction^10–12^ (Extended Data Fig. 6e). Most of these changes normalized by day 6 post-iron, with the notable exceptions of neutrophil-derived *Tgfb1* and fibroblast- and type II alveolar epithelial cell-derived *Tgfb3* (Extended Data Fig. 6e), both factors being involved in the conversion of fibroblasts into collagen-producing cells^55^. In line with this, 6 d post-iron we observed a strong induction in the levels of *Col1a1*, *Col1a2* and *Fn1* in fibroblasts, most prominently in the fibroblast 2 cluster (Fig. 6d). We found that iron accumulation was a driver of the expansion of fibroblast 2 cells, as suggested by the strong correlation between ferritin levels and the expression of a cell proliferation signature in this cell type (Fig. 6e). We observed a similar phenomenon in goblet cells (Extended Data Fig. 7a). Of note, we found that while most cells presented with baseline levels of ferritin expression at day 6 post-iron, a small fraction of fibroblast 2 cells presented with extremely high ferritin levels in association with abnormally high expression of *Cdkn1a* (Fig. 6f). Cdkn1a^high^/ferritin^high^ fibroblast 2 cells differed from the rest of the day 6 post-iron fibroblasts in that they did not express collagens (Extended Data Fig. 7b). Low expression of collagens and high expression of p21 are features of senescent fibroblasts^56^. Together, these observations suggest that sustained iron accumulation is a feature of in vivo senescence, which is in agreement with previous data in vitro (our own data; Fig. 6 and Extended Data Fig. 6 and published reports^33,34^).

Our snRNA-seq data allowed us to identify a set of genes that are strongly induced across most cell types in response to iron in vivo. We named this gene set ‘iron accumulation signature’ (IAS) (Supplementary Data Table 2). To identify cells which may contribute to iron accumulation in the context of human fibrotic diseases, we analyzed a scRNA-seq meta-analysis from patients with IPF for the enrichment of the IAS. IPF lung tissues compared to control lungs, showed significant enrichment of the IAS genes in the bulk lung tissue and in multiple individual cell types (Fig. 6g and Extended Data Fig. 7c) with the highest enrichment scores and expression observed in type II alveolar epithelial cells, fibroblasts and goblet cells (Fig. 6g,h). Notably, IAS expression strongly correlated with the expression of *CDKN1A* and with senescence (SenMayo signature), with cells double positive for the IAS and senescence markers (CDKN1A^high^/IAS^high^ and SenMayo^high^/IAS^high^) being significantly more abundant in patients with IPF compared to control donors, both when studied in the whole lung or in several individual cell types (Fig. 6i–l and Extended Data Fig. 7d,e). Overall, these findings strongly suggest that senescent cells may be important contributors to iron accumulation in fibrotic tissues.

### Iron detected by MRI as a biomarker of kidney fibrosis

**Fig. 7:**
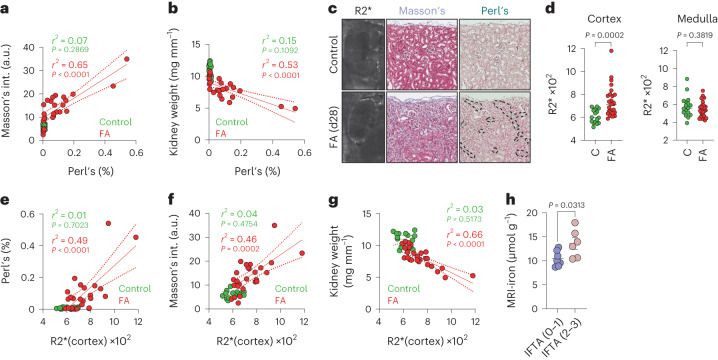
Iron detected by MRI as a biomarker of kidney fibrosis. **a**,**b**, Correlation analysis between Perl’s and Masson’s trichrome staining intensity (**a**) and Perl’s staining intensity (**b**) and kidney weight in mice. Each dot represents a mouse kidney from mice 28 d post-i.p. PBS (green; *n* = 16) or i.p. FA (red; *n* = 26). **c**, Representative images of R2* map acquired by MRI, Masson’s trichrome staining and Perl’s staining of kidneys from mice 28 d after injection with PBS or FA. **d**, Mean R2* value in the kidney cortex and in the kidney medulla of mice 28 d after treatment with i.p. injection of PBS or FA. Data were analyzed by Mann–Whitney *U*-test. **e**–**g**, Correlation analysis between the mean R2* value measured in the kidney cortex and intensity of Perl’s staining in the kidney cortex (**e**), intensity of Masson’s staining in the kidney cortex (**f**) and kidney weight (**g**). Each dot represents a mouse kidney from mice receiving i.p. PBS (green; *n* = 16) or i.p. FA (red; *n* = 26) analyzed 28 d after injection. **h**, R2* values in the kidney cortex were measured in kidney allograft recipient patients (*n* = 13). We compared R2* values obtained in patients with no or moderate fibrosis (IFTA (0–1); *n* = 7) to patients with pronounced fibrosis (IFTA (2–3); *n* = 6). Bar graphs represent mean ± s.e.m.; data were analyzed by a two-tailed unpaired *t*-test. In all panels, when bar graphs are shown, they represent mean ± s.e.m.; when linear regression is shown, dashed lines represent the 95% confidence interval; dots represent individual animals or humans. Unless stated otherwise, we compared distributions using two-tailed unpaired *t*-test. Source data

**Extended Data Fig. 8:**
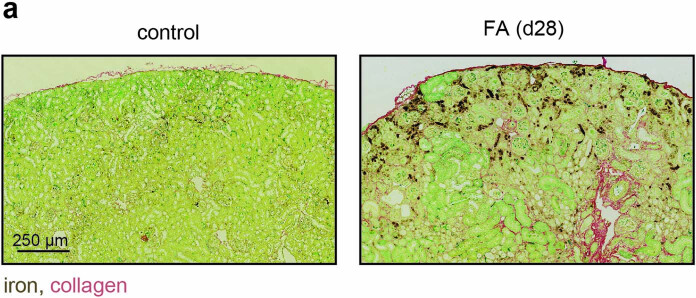
Iron detected by MRI as a biomarker of kidney fibrosis. **a**, Representative images of histochemistry for iron and collagen by combining enhanced Perl’s Prussian blue and Sirius Red/Fast Green staining in kidney sections from mice 28 days after intraperitoneal injection of vehicle (control) or folic acid (FA).

We wondered whether iron accumulation in fibrotic tissues could be used as a proxy to detect fibrosis by non-invasive medical imaging. Before this, we documented that the percentage of Perl’s-positive cells was directly correlated with Masson’s trichrome staining (Fig. 7a) and inversely correlated with kidney weight (Fig. 7b) in mice treated with FA. Of note, the majority of Perl’s-positive cells were located cortically in the FA-treated kidneys, whereas medullae were largely negative (Extended Data Fig. 8a). Iron can be detected by magnetic resonance imaging (MRI) using R2* relaxation mapping^57^. Similar to the histological observations by Perl’s staining, R2* mapping detected a significantly elevated signal in the cortex, but not in the medulla, of the kidneys of FA-treated mice (Fig. 7c,d). The R2* signals in the cortices of individual kidneys correlated positively with their corresponding Perl’s (Fig. 7e) and Masson’s staining (Fig. 7f) and correlated negatively with kidney weight (Fig. 7g).

To evaluate whether this method can be utilized in a clinical setting, we performed a pilot study on renal allograft recipients. A substantial proportion (40%) of these patients develop kidney fibrosis in their transplanted kidneys within 3–6 months after transplantation. The monitoring of kidney fibrosis is complex and requires invasive biopsies^58^. We analyzed 13 patients by MRI for their R2* signal in the kidney cortex and for their level of fibrosis measured on biopsies (IFTA score). We found that patients with high IFTA scores (2 and 3) presented with significantly higher cortical R2* signals than patients with low IFTA scores (0 and 1) (Fig. 7h). Analysis of available clinical information suggested that the increased R2* signal in patients with high IFTA scores was not due confounding factors that can alter iron homeostasis, such as comorbidities, treatments for iron deficiency or altered erythropoiesis (Supplementary Data Table 3). Collectively, we conclude that iron measurement by MRI may be a clinically translatable tool for non-invasive detection of fibrotic tissue remodeling.

## Discussion

Iron accumulation in the context of fibrosis has been reported previously^30–32,59^. Here, we show that this is not dependent on a specific organ or insult, but is an early event upon tissue injury that, once initiated, progresses and becomes a distinctive feature of multiple tested fibrotic diseases. We demonstrate that exposure to iron overload, such as that caused by direct administration of iron or vascular or hemolytic insults, can in some circumstances initiate fibrogenesis and senescence. We also show that the accumulation of iron in fibrotic lesions continues even when the initial burst of extracellular iron caused by tissue injury has subdued. This has led us to discover that iron accumulation is an intrinsic feature of damaged cells regardless of the type of damaging insult and normal conditions of extracellular iron. Therefore, for the purposes of this discussion, it is helpful to distinguish between (1) the role of ‘excessive extracellular iron’ as a potential pathophysiological trigger of senescence and fibrosis and (2) the role of ‘excessive intracellular iron’ as a driver of the pathological effects of senescent cells through the SASP.

Regarding the role of ‘excessive extracellular iron’, there is abundant evidence linking hereditary and acquired hemochromatosis to fibrotic diseases^24,26–29^. Our data extend this concept to fibrotic diseases unrelated to hemochromatosis. Many fibrotic processes are of unknown etiology. We present data indicating that small vascular injuries with associated iron release could in some circumstances be contributors to fibrotic processes. Indeed, some human fibrotic pathologies have been associated to vascular problems. This is the case for systemic sclerosis, a disease characterized by multi-organ fibrosis, in which vascular insufficiency manifests early in the disease^60,61^. Also, patients with complement-mediated hemolytic anemia are at risk of developing fibrosis in their kidneys, a condition known as hemolytic uremic syndrome^62^. We have found that iron potently suppresses VEGF levels and causes capillary rarefaction. VEGF is known for its effects to promote blood flow in the local circulation^63^ and it is attractive to speculate that perhaps vascular rarefaction, as observed in fibrotic conditions^10,11,48^, is an attempt by the body to limit bleeding. Our findings suggest that in some fibrotic conditions, excessive iron accumulation may directly promote fibrosis; however, this may not be necessarily required for fibrosis more generally.

Regarding ‘excessive intracellular iron’, we report that this can be initiated by cellular damage and progressively increases together with the development of the senescent phenotype. This process does not require the presence of abnormal amounts of extracellular iron, instead it is an intrinsic feature of cell cycle exit via senescence. Non-dividing cells need very little iron. Dividing cells, in contrast, take up large amounts of iron, because they need to duplicate their iron content to generate two daughter cells. We have found that when cells exit the cell cycle via senescence, as opposed to quiescence or dormancy, they do not downregulate their TfR; however, while proliferating cells split their intracellular iron content between daughter cells with every proliferation cycle, senescent cells accumulate iron with no possibility of splitting it by cell division. This is associated with a progressive increase in ferritin-bound ferric iron, which we have found to mainly reside in lysosomes, and to abnormally high levels of labile, ferrous iron, which drives the senescence-associated ROS. Indeed, treatment of senescent cells with an iron chelator strongly reduces their ROS levels. Previous reports have connected ROS with the secretory phenotype of senescent cells^51,52^. Based on this, we also show that iron chelation efficiently suppresses the SASP, thereby mechanistically connecting damage-initiated iron uptake with ROS and the SASP. The intrinsic and cell-autonomous accumulation of intracellular iron by damaged and senescent cells explains why, during fibrogenesis, iron continues accumulating even in the presence of normal extracellular iron levels.

Ultimately, fibrosis is mediated by the expansion and TGF-β-dependent differentiation of fibroblasts into myofibroblasts^8^. Using snRNA-seq, we have found that iron is a potent inducer of both processes. We observed that iron uptake promoted proliferation and ultimately differentiation of lung fibroblasts into collagen-producing cells. Notably, we observed that a small fraction of fibroblasts maintained high levels of iron uptake. These iron-accumulating fibroblasts presented with expression of senescence-associated markers. The association between iron uptake and senescence was not restricted to mice administered with intratracheal iron. We have found that the lung transcriptome from patients with IPF was significantly enriched in the iron accumulation signature (IAS) and this was due to the robustly elevated IAS expression in p21^+^ fibroblasts, goblet cells and epithelial cells. IAS-positive cells in IPF were not only p21^+^ but also expressed high levels of senescence-related genes (SenMayo signature), which are dominated by SASP factor genes, suggesting that iron accumulation in IPF is inherently linked to SASP-producing pathological senescent cells. In agreement with our findings in vitro and in vivo, we have found that DP strongly reduces the SASP and is able to protect mice from FA-induced kidney fibrosis. There is considerable interest in finding drugs targeting senescent cells, which includes drugs that kill senescent cells (senolytic drugs) and drugs that reduce the SASP (senomorphic drugs)^64^. Each of these approaches has its own benefits and risks. Our data points out the possibility of using DP, a clinically approved drug, as a senomorphic agent.

Recent findings suggest that ferroptosis, an iron dependent form of cell death may play an important role in the pathobiology of fibrosis^65^. These findings together with ours, further reinforce the importance of deregulated iron metabolism in fibrotic diseases.

Finally, we demonstrate in a proof-of-principle study that iron detection by MRI could be a useful diagnostic tool to identify fibrotic remodeling early. MRI-based iron detection has been first used in a clinical setting in a seminal study on patients with thalassemia major to detect iron accumulation in the heart^57^. Later studies demonstrated that MRI-based iron detection allows for assessment of iron deposition in the liver, spleen and other organs and thereby for the clinical assessment of patients with hereditary hemochromatosis or hemoglobinopathies^66,67^. Here, we show that the same technology can be applied to assess fibrotic burden non-invasively in mice and patients with kidney fibrosis. These individuals have no genetic or acquired iron accumulation syndrome but accumulate iron in their kidneys in association with fibrosis. We find that iron levels correlate with the grade of fibrosis and with kidney atrophy and urge other clinicians to test, in the context of fibrotic diseases, the same method for its utility to improve early detection of fibrotic disease.

In summary, our study establishes iron accumulation as a clinically exploitable driver of pathological senescence and fibrosis.

## Methods

### Cells

Human melanoma cells (SK-MEL-103, kindly provided by M.S. Soengas, Spanish National Cancer Research Centre), mouse endothelial cells (H5V), HUVECs (H5V and HUVECs were both kindly provided by R. Mendez, Institute for Research in Biomedicine; IRB), human lung fibroblasts (IMR90, ATCC, CCL-186) and MEFs (isolated as described^68^) were cultured in standard DMEM with 10% FBS and penicillin–streptomycin (all from Gibco) in 20% O_2_ and 5% CO_2_ at 37 °C. The medium of HUVEC cells contained 2% FBC and was supplemented by endothelial cell growth medium (Sigma, C-22110). Cells were routinely tested for *Mycoplasma* contamination using the *Mycoplasma* tissue culture NI (MTC-NI) Rapid Detection System (Gen-Probe).

### Mice

All mice were housed under specific-pathogen-free conditions in individual ventilated cages in a controlled environment room (temperature 20–24 °C, relative humidity 30–70% and positive pressure) under a 12-h light–dark cycle and allowed unrestricted access to food and water (wild-type (WT) and *Tie2-Cre-ERT2 Rosa26-iDTR* in the Parc Cientific de Barcelona Animal Facility, with registration number B-9900044). Environmental parameters were controlled using the software Controlli Delta Spain (v.3.0). Protocols were approved by the Animal Care and Use Ethical Committee of animal experimentation of the Barcelona Science Park and the Catalan Government. *TG*^Adrd1^ mice, overexpressing the human β1-adrenoceptor in cardiac myocytes driven by the α-myosin heavy chain promoter, develop spontaneous cardiomyopathy, mimicking human heart failure, were described earlier^69^. The *TG*^Adrd1^ mice on a Friend Virus B NIH (FVB/N) background were housed in the Institute of Pharmacology and Toxicology at the Technical University of Munich. Male transgenic mice and WT littermates were analyzed at the age of 2.5, 5 and 10–12 months. *Tie2-Cre-ERT2 Rosa26-iDTR* mice were on a mixed background (129S4/SvJae and C57BL/6J) generated by crossing *Tie2-Cre-ERT2* mice (kindly donated by S. Ortega, to be described in a future paper) to *Rosa26-iDTR* mice^70^. For all other studies, we used 8–10-week-old C57BL/6J mice. Where female mice were used, this is specifically indicated. Euthanasia of animals was performed by CO_2_ or by cervical dislocation.

### Senescence induction in vitro

To induce senescence, cells were plated subconfluent. For damage with classical senescence inducers, 1 μM palbociclib (PD033299, Pfizer), 100 nM doxorubicin (D1515, Sigma) or 10 mU ml^−1^ bleomycin (B8416, Sigma), cells were cultured in the presence of the toxin in 10% FBS containing DMEM for 2 d, after which cells were washed and replenished with complete culture medium. To induce senescence by iron, heme or lysed RBCs, cells were first deprived of iron for a day by culturing them in 0.5% FBS containing RPMI medium (RPMI does not contain iron), followed by culturing cells for 3 d, the same medium supplemented with a sterile filtered (0.22-µm pore size syringe filters) aqueous solution of Fe(II)sulfate (330 μM; F8633, Sigma) and Fe(III)nitrate (330 μM; F8508, Sigma) or with lysed RBCs (25-fold dilution; for the preparation, see next section) or with hemin (10 μM; H9039, Sigma Aldrich), after which cells were washed and replenished in DMEM medium containing 10% FBS. To induce senescence by irradiation, cells were exposed to a dose of 20 Gy irradiation. To induce oncogene-induced senescence, we used IMR90 cells stably transduced with a tamoxifen inducible Ras-G12V (a gift from D. Muñoz Espin, University of Cambridge) construct. Cells were cultured in the presence of 4-hydroxy tamoxifen (1 μM) for 21 d. When testing for the possible prevention of senescence by iron chelation, we added DP (200 µM) together with the senescence inducers for 4 d, after which the drugs were removed and cells were maintained for another 3 d in culture. When testing whether iron chelation could reverse senescence, senescence was induced for 7 d, after which cells were cultured in the presence of the iron chelator DP (200 µM) or vehicle (water) in normal culture medium for a further 4 d.

### Induction of quiescence and dormancy

To induce quiescence, cells were cultured in DMEM containing 1% FBS for 1 d, followed by culture in DMEM containing 0.5% FBS for 6 d. To induce dormancy^71^, cells were cultured in the presence of mTORC1/mTORC2 inhibitor INK128 (200 nM) for 7 d.

### BrdU staining

Cells were cultured with 5-bromodeoxyuridine (BrdU) (Sigma Aldrich, B5002, 10 μM) for 90 min. After culture with BrdU, cells were fixed (4% PFA) and permeabilized (0.1% Triton X-100 for 5 min). Following washing in PBS, cells were resuspended in 4 M HCl and incubated for 15 min. Blocking was performed in 1% BSA + 0.5% Tween (blocking buffer) for 20 min. The BrdU antibody (Santa Cruz Biotech, sc-32323) was diluted (1:500) in blocking buffer and incubated on the cells overnight at 4 °C. The secondary antibody was a goat anti-mouse IgG (H + L) Alexa Fluor 488 (Thermo Fisher, A-11001), which was used at a 1:500 dilution. To counterstain DNA, mounting medium containing 4′,6-diamidino-2-phenylindole (DAPI) was used (H-1200-10, VECTASHIELD). Cells were imaged and the percentage of BrdU-positive cells was quantified.

### Flow cytometry analysis

For analyzing viability, cells were stained with an annexin-V and propidium iodide (Thermo Fisher, 88-8007-74) apoptosis detection kit according to the manufacturer’s instructions. For analyzing lung infiltration, lungs were intubated ex vivo through the trachea with 2 ml dispase solution (5 U ml^−1^) and then agitated at 37 °C. Lungs were chopped and dissociated in 1% BSA + 60 U ml^−1^ DNase1 + 70 U ml^−1^ collagenase type 1 dissolved in PBS with the help of a GentleMACS system (Miltenyi) according to the manufacturer’s instructions. RBCs were removed in ammonium-chloride-potassium lysis buffer and debris was removed using cell strainers. Cells were stained on 4 °C in PBS with 1% FBS using a 1:300 dilution of anti-CD45-PE, anti-CD45-APC, anti-CD11b-PE/Cy7 (all from BD) and anti-Ly6G-PE, anti-CD11b-Pacific blue and F4/80-APC (all from BioLegend) in the presence of a blocking antibody, rat anti-mouse CD16/CD32 (BD; 1:600 dilution). Labile iron was measured using the FerroOrange Dye (F374, Dojindo), total ROS was measured using a total ROS detection kit (ENZ-51011, Enzo) and lysosomal mass was assessed by using a LysoTracker dye (L12492, Invitrogen), all according to the manufacturer’s instructions. Samples were acquired on a Gallios flow-cytometer (Beckman Coulter) and acquired data were analyzed with FlowJo software (Tree Star).

### Preparation of lysed RBCs and considerations related to dosage of iron

Fresh blood was collected from mice in EDTA-coated tubes. Blood was diluted in 1 mM EDTA in PBS and the corpuscular fraction was isolated by three rounds of centrifugation at 600*g* and washed twice. Supernatant was removed and replaced by a low volume of hypotonic 1× RBC lysis buffer (00-4333-57, eBioScience) (100 μl RBC lysis buffer per 500 μl corpuscular fraction). RBCs were vigorously vortexed and passed through a gauge syringe multiple times, until a homogeneous, viscous red solution formed. This solution of lysed RBCs was resuspended in equal volumes of PBS and supplemented to tissue culture in a 25-fold dilution to mimic microhemorrhagic or hemolytic insult. We found estimates in the literature that after removal of plasma, each ml of packed RBCs contains 1 mg of iron^72^, corresponding to 17.9 mM. Therefore, we delivered a concentration of free iron (16.5 mM) that approximates the local iron concentration of an RBC immediately after hemolysis. When comparing the effects of iron to those of lysed RBCs, we used the same dilutions of RBC and iron solutions. When delivering iron into the lungs, we delivered 30 μl 16.5 mM iron solution to mimic a hemorrhagic insult caused by 30 μl blood.

### Models of kidney fibrosis

We used male C57BL/6J mice at 8–10 weeks of age. For FA-induced kidney injury, mice were injected i.p. with a single dose of FA (250 mg kg^−1^; F7876, Sigma) dissolved in 300 mM sodium bicarbonate (NaHCO_3_; Sigma). Dry kidney weight was normalized to the tibia length of the animals. For iron chelation, animals received daily i.p. injections of 200 μl 1 mg ml^−1^ DP (379409, Sigma) dissolved in water; control animals were injected with only water. For the unilateral ureteral obstruction model, animals were anesthetized with isoflurane plus oxygen and the left ureter was exposed through a flank incision. The ureter was ligated with 8-0 nylon, whereas in sham-operated mice the ureter was left undisturbed.

### Models of lung fibrosis

To induce pulmonary fibrosis, 8–10-week-old C57BL/6J WT mice were anesthetized by i.p. injection of ketamine (75 mg kg^−1^) and medetomidine (1 mg kg^−1^). Animals were placed on a Tilting WorkStand for rodents (EMC Hallowell) and intubated intratracheally with a 24GA catheter (BD Biosciences) and delivered 30 μl bleomycin (1.5 U kg^−1^ of body weight) (Sigma, 15361) or 30 μl aqueous iron solution containing a concentration of 8.3 mM Fe(II)sulfate and 8.3 mM Fe(III)nitrate (in total 16.5 mM iron); control animals received 30 μl PBS. Iron solution was sterile filtered with a 0.22-µm pore size syringe filter. Mice receiving intratracheal iron developed sepsis-like symptoms within a day, with severe weight-loss, from which they recovered on the fourth day post-iron. We found that treatment with a daily dose of subcutaneous buprenorphine (0.1 mg kg^−1^) considerably mitigated these symptoms and the overall mortality in this model remained very low (<10%).

### Ischemic heart fibrosis

Male 3–4-month-old C57BL/6J mice were used. Intraoperative analgesia was induced by treating mice with buprenorphine (0.1 mg kg^−1^) 60 min before anesthesia. Anesthesia was induced with 5% isoflurane and maintained at 2% with mechanical ventilation following endotracheal intubation (2% isoflurane/97% oxygen, 130–140 stroke rate, stroke volume initially 5 ml kg^−1^ increased to 7.5 ml kg^−1^ post‐thoracotomy). Animals were placed on a heating table during the surgery and eyes were covered with ophthalmic gel (LACRYVISC 3 mg g^−1^, Alcon) to protect the corneas. Neck hair and hair in the upper thorax was removed. An incision was made, followed by a left-sided thoracotomy at the fourth-intercostal space. The pericardium was removed and the heart left anterior descending artery was located between the pulmonary artery and the left auricle and was ligated using an 8-0 Prolene suture (PremiCron- B Braun Surgical). The thoracic incision was closed in layers, using 6-0 Prolene sutures (PremiCron- B Braun Surgical) to adapt the ribs and 4-0 Prolene sutures (PremiCron- B Braun Surgical) to close the skin. A heating lamp was used to warm the animals until they fully recovered from the anesthesia. Analgesic treatment continued during the three days after surgery. Sham-operated mice were used as a control.

### Histochemical staining

For H&E staining, paraffin-embedded tissue sections were dewaxed and stained with hematoxylin eosin standard staining using a CoverStainer (Dako - Agilent). An Iron Stain kit was used to identify iron pigments (AR15811-2, Artisan, Dako, Agilent) and a Masson’s Trichrome Stain kit was used to identify collagen fibers and fibrin (AR17311-2, Artisan, Dako, Agilent). Staining was performed in a Dako ArtisanLink (Dako, Agilent) staining system according to the manufacturer’s instructions. For the SRFG staining, tissue samples were incubated with the mordant thiosemicarbazide 99% (Sigma, T33405) for 10 min, washed in distilled water and therefore incubated with 0.1% Fast green (Sigma, FCF F7552) for 20 min and finally rinsed with 1% acetic acid (Sigma, 320099) for 1 min. To enhance iron detection, we adapted the EPPB staining protocol^73^ by adding a blocking step with 5% normal goat serum (16210064, Life Technology) with 2.5% BSA (10735078001, Sigma) for 60 min followed with 30 min of peroxidase-blocking solution (S2023, Dako-Agilent) and a 30 min incubation with Liquid DAB+ Substrate Chromogen System (K3468, Dako-Agilent) on sections that had been previously stained with the Iron Stain kit. To detect ferrous iron, sections were incubated with ammonium sulfide solution (Sigma, 515809) for 60 min and then immersed in a 1:1 mixture of 20% potassium ferricyanide (Sigma, 70258) and 1% HCl solution in distilled water for 10 min. A counterstain with nuclear fast red solution was performed for 2 min and ferrous iron detection was enhanced by adaptation of the enhanced Turnbull blue^73^ staining, using a blocking step with 5% normal goat serum (16210064, Life Technology) with 2.5% BSA (10735078001, Sigma) for 60 min followed by 30 min of peroxidase-blocking solution (S2023, Dako-Agilent) and a 30-min incubation with Liquid DAB+ Substrate Chromogen System (K3468, Dako-Agilent). In all cases, samples were dehydrated and mounted with Mounting Medium, Toluene-Free (CS705, Dako, Agilent) using a Dako CoverStainer.

### Immunohistochemistry staining

Immunohistochemistry was performed manually on a Ventana discovery XT platform and in a Leica Bond RX platform. Primary antibodies were incubated on sections in a dilution and for the time as follows: p21 clone HUGO 291H/B5 (CNIO) RTU 60 min; p21 WAF1/Cip1 SX118 (Dako-Agilent, M7202) at 1:50 dilution for 60 min; NE (Abcam, ab68672) at 1:1,000 dilution for 120 min; F4/80 D2S9R (Cell Signalling, 70076) at 1:1,000 dilution for 60 min; α-SMA (1A4) (Abcam, ab7817) at 1:750 dilution for 120 min; HMOX1 (Abcam, ab13243) at 1:1,250 dilution for 60 min; and TER-119 (Stem Cell, 60033) at 1:5,000 dilution for 60 min. Antigen retrieval for p21 was performed with Cell Conditioning 1 (CC1) buffer (Roche, 950-124). Secondary antibodies used were the OmniMap anti-rat HRP (Roche, 760-4457) or OmniMap anti-Rb HRP (Roche, 760-4311). Blocking was performed with casein (Roche, 760-219). Antigen–antibody complexes were revealed with a ChromoMap DAB kit (Roche, 760-159). p21 WAF1/Cip1 SX118 were incubated with the rabbit to IgG1 + IgG2a + IgG3 (M204-3) (Abcam, ab133469) at a 1:500 dilution for 32 min before adding the secondary antibody. Before immunohistochemistry, sections were dewaxed and epitope retrieval was performed using citrate buffer, pH 6, for α-SMA and Ter-119 or Tris-EDTA buffer, pH 9, for HMOX1 in all cases for 20 min at 97 °C using a PT Link (Dako, Agilent) or with ER2 buffer (AR9640, Leica) for 20 (F4/80) or 30 min (NE). Washing was performed using the Wash Solution AR (AR10211-2, Dako, Agilent) or with the BOND Wash Solution 10× (AR9590, Leica). Quenching of endogenous peroxidase was performed by 10 min incubation with peroxidase-blocking solution at room temperature (S2023, Dako, Agilent). Nonspecific unions were blocked using 5% normal goat serum (16210064, Life Technology) mixed with 2.5% BSA diluted in a wash buffer for 60 min at room temperature. Blocking of nonspecific endogenous mouse immunoglobulin staining was also performed using a Mouse on Mouse (MOM) Immunodetection kit (BMK-2202, Vector Laboratories). Secondary antibodies used were BrightVision poly-HRP-anti-rabbit IgG biotin-free, ready to use (DPVR-110 HRP, Immunologic), HRP-anti-rat IgG (MP-7444, Vector) for 45 min or goat anti-mouse immunoglobulins/HRP (Dako-Agilent, P0447) at 1:100 dilution for 30 min. Antigen–antibody complexes were revealed with 3-3′-diaminobenzidine (K346811, Dako) or DAB (polymer) (Leica, RE7230­CE) with the same time exposure. Sections were counterstained with hematoxylin (CS700, Dako, Agilent) and mounted with Mounting Medium, Toluene-Free (CS705, Dako, Agilent) using a Dako CoverStainer. Specificity of staining was confirmed with the following isotype controls: rabbit IgG, polyclonal (Abcam, ab27478), mouse IgG1, κ (NCG01) (Abcam, ab81032), mouse IgG2a κ (eBM2a) (eBioscience, 14-4724-82) and rat IgG (R&D Systems, 6-001-F).

### Image acquisition and quantification of histological sections

Brightfield images were acquired with a NanoZoomer-2.0 HT C9600 digital scanner (Hamamatsu) equipped with a ×20 objective. All images were visualized with a gamma correction set at 1.8 in the image control panel of the NDP.view 2 U12388-01 software (Hamamatsu, Photonics). Image quantification was performed using QuPath software^74^. When clinical samples were analyzed, trained pathologists analyzed the tissues and used a scoring system in accordance with their daily clinical practice. When quantifying the temporal changes in EPPB staining in FA-induced kidney fibrosis, we analyzed EPPB^+^ pixels. When quantifying the temporal changes in EPPB staining in bleomycin-induced lung fibrosis, we delineated and quantified the percentage of EPPB^+^ areas, defined as areas rich in iron-accumulating cells as a percentage of total lung area. Blinding was ensured where possible by automating the analysis in QuPath.

### SA-β-GAL staining

For senescence-associated β-galactosidase staining, cells were fixed in an SA-β-GAL fixation solution (PBS with 0.1 M EGTA, 1 M MgCl_2_ and 50% glutaraldehyde) for 15 min at room temperature and washed twice with PBS. Subsequently, a staining solution at pH 6 was added (PBS with 1 M MgCl_2_, 0.5 M K_4_(Fe(CN)_6_) and 0.5 M K_3_(Fe(CN)_6_) with 1 mg ml^−1^ X-GAL diluted in dimethylformamide (all from Sigma)). The incubation took place overnight at 37 °C in a CO_2_-free incubator.

### Measurement of free hemoglobin in the serum

We collected serum samples from mice and analyzed them for levels of free hemoglobin by using a mouse hemoglobin ELISA kit (ab157715, Abcam), according to the manufacturer’s instructions.

### Ex vivo RBC hemolysis assay

Fresh blood was collected from mice in EDTA-coated tubes. Blood was diluted in 1 mM EDTA in PBS. Corpuscular fraction was isolated by three rounds of centrifugation at 600*g*, washed and resuspended in four volumes of PBS. Then, 100 μl RBC suspension per well in a round-bottom 96-well plate was incubated in the presence of a serial dilution of FA, 0.05% Triton X-100 (T8787, Sigma) (both in PBS) or PBS alone for 6 h at room temperature.

### Gene set variation analysis

Gene set variation analysis^75^ was used to determine the relative expression scores of the GO_IRON_IRON_TRANSPORT Gene Ontology (0006826) in patients and controls from the two datasets of the lung tissue consortium (GSE47460).

### qPCR

Total RNA was isolated using Trizol (Invitrogen) or the RNeasy Micro kit (QIAGEN). Complementary DNA was synthesized using the iScript cDNA synthesis kit (Bio-Rad). PCR was performed using the GoTaq SYBR Green qPCR Master Mix (Promega) and gene-specific primers (Supplementary Data Table 4) and analyzed using the 2^-ΔΔCT^ method. Relative expression values were normalized to the average of the controls.

### Endothelial cell ablation in *Tie2-Cre-ERT2*^*+*^*Rosa26-iDTR* mice

Tamoxifen (Sigma) was freshly dissolved in corn oil. For ablation of endothelial cells, mice were injected with tamoxifen (50 µg g^−1^ body weight; Sigma) daily for 1 week followed by 2 d of resting, and then i.p. injection of diphtheria toxin (30 ng g^−1^ body weight; Sigma) for five consecutive days.

### Western blotting

Cells were collected after treatment and lysed in a buffer of 50 nM Tris-HCl, pH 8, 1 mM EDTA, 150 mM NaCl, 1% NP40, 0.5% Triton X-100 and 1% SDS and freshly supplemented with protease inhibitors (Roche, 11873580001). Then, 15 μg whole lysates per lane and a Chameleon Duo pre-stained protein ladder (LI-COR, 928-60000) were resolved on 4–12% Bis-Tris gels (NuPAGE Invitrogen, NP0321BOX) and transferred to Amersham Protran 0.2-μm nitrocellulose membranes (GE Healthcare, 10600001). Blots were blocked with a LI-COR blocking buffer and incubated with the primary antibodies, namely anti-FTH1 (3998S, Cell Signaling), anti-β-actin (A5441, Sigma), anti-CD71/TfR (Cell Signaling, D7S5Z, 1:1,000 dilution), anti-ZIP14 (PA5-21077, Thermo Fisher, 1:1,000 dilution) and anti-GAPDH (ab9485, Abcam, 1:2,000 dilution) at 4 °C overnight and subsequently incubated with the corresponding secondary anti-IgG antibodies, anti-rabbit IRDye 680 CW (1:15,000 dilution, LI-COR, 926-68071), anti-rabbit IRDye 800 CW (1:15,000 dilution, LI-COR, 926-32211) and anti-goat IRDye 680 CW (1:10,000 dilution, LI-COR, 926-68074) for 1 h at room temperature. Blots were analyzed with an Odyssey Fc imaging system (LI-COR). For densitometry, OD of the protein of interest relative to the housekeeping gene was normalized to the averages obtained in control samples.

### Transmission electron microscopy

Cells were fixed at 4 °C in a mixture of 2% PFA and 2.5% glutaraldehyde in 0.1 M phosphate buffer (PB), then scraped, pelleted, washed in PB and postfixed in 1% osmium tetroxide and 0.8% potassium ferrocyanide. Samples were dehydrated with acetone and embedded in Spurr resin. Ultrathin sections were obtained using an Ultracut E (Reichert), picked up on copper grids and observed with the JEM 1011 (JEOL) electron microscope, operating at 80 kV. Micrographs were taken with a camera (Orius 1200A; Gatan) using the DigitalMicrograph software package (Gatan). Electron micrographs were processed using Adobe Photoshop CS6 (v.13.0.1) (Adobe Systems).

### Measurement of total iron levels

Total iron levels were measured by a colorimetric assay (MAK025-1KT, Sigma) according to the manufacturer’s instructions.

### Multiplex protein assays

Lung tissues or cells were lysed in normal RIPA buffer. Samples were shipped to an external commercial laboratory (Eve Technologies) to perform three types of routine discovery on lung tissues (MD44, MDCVD1 and MDMMP-C,O) and one type on senescent and non-senescent MEFs (MD44). Mouse Cardiovascular Disease Panel 1 7-Plex Discovery Assay Array (MDCVD1): MMP-9, PAI-1/SERPINE1 (total), Pecam-1, sP-Selectin, sE-Selectin, sICAM-1 and thrombomodulin. Mouse Cytokine/Chemokine 44-Plex Discovery Assay Array (MD44): eotaxin, erythropoietin, 6Ckine, Fractalkine, G-CSF, GM-CSF, IFNB1, IFN-γ, IL-1α, IL-1β, IL-2, IL-3, IL-4, IL-5, IL-6, IL-7, IL-9, IL-10, IL-11, IL-12p40, IL-12p70, IL-13, IL-15, IL-16, IL-17, IL-20, IP-10, KC, LIF, LIX, MCP-1, MCP-5, M-CSF, MDC, MIG, MIP-1α, MIP-1β, MIP-2, MIP-3α, MIP-3B, RANTES, TARC, TIMP-1, TNF-α and VEGF-A. Mouse MMP 5-plex Discovery Assay Array for Cell Culture and non-blood samples (MDMMP-C,O): MMP-2, MMP-3, MMP-8, proMMP-9 and MMP-12. The proteins shown in the results changed significantly by the investigated insult.

### Single-nuclei RNA sequencing

Male mice received intratracheal PBS or iron as described, were killed in CO_2_ chambers, lungs were removed, separated into lobes, frozen on dry ice and stored at −80 °C. Nuclei were isolated from lung lobes using the Chromium 10x nuclei isolation kit following the manufacturer’s instructions. Three single-nuclei suspensions were used for transcriptome analysis at IRB Barcelona Functional Genomics Core Facility. Isolated nuclei were filtered using a 40-μm flowmi cell strainer (SP Bel-Art) and quality controlled on a LUNA-FL automated fluorescence cell counter (Logos Biosystems). The suspensions were used to target between 10,000 and 12,000 nuclei per sample. Partition into GEMs (Gel beads-in-emulsion, which are single-nuclei emulsion droplets) was performed using Chip G and the 3′ v.3.1 chemistry (10x Genomics). Barcoded cDNA was amplified for 13 cycles, quantified and quality controlled on the Bioanalyzer 2100 using a high sensitivity DNA assay (Agilent). The generated Illumina-compatible libraries were quantified using the Qubit dsDNA HS assay (Invitrogen), quality controlled with the Bioanalyzer 2100 DNA HS assay (Agilent) and used to generate an equimolar pool. The sequencing pool was loaded at 1.8 pM in two 150-cycle hi-output NextSeq550 flow cells (Illumina) using the following sequencing strategy: read 1, 28 cycles; i7, ten cycles; i5, ten cycles; and read 2, 91 cycles. Over 888 million reads were produced with a minimum of 231 million reads per sample.

### Single-nuclei RNA-seq analysis

Chromium scRNA-seq reads were aligned to the reference transcriptome (refdata-gex-mm10-2020-A) with CellRanger^76^ v.7.1.0. The count utility was used to quantify gene expression. Following the recommendations for analyzing single-nuclei transcriptomic data, intronic reads were included in the count matrix^77^. The remaining parameters were set to their default values. The subsequent processing steps and analysis were performed with Seurat package v.4.1.1 (refs. ^78–80^). Cells with <20% mitochondrial read content and >1,000 RNA molecules detected were considered for downstream analyses. Ribosomal reads were removed. The proportion of mitochondrial reads was regressed out during the normalization and variance stabilization of raw counts, which was performed with the sctransform method^81^. The first 15 principal components were used to obtain the UMAP for visualization purposes. Cells were assigned to clusters using FindClusters Seurat function (resolution of 1.2). SCT-transformed counts were further imputed and smoothed with Magic (v.2.0.3)^82^. The expression of gene signatures was summarized by taking the average Magic score of its constituent genes. Substantial differences were observed across the three conditions. To facilitate cell type annotation, the IntegrateData function was used with pre-computed anchors obtained from 3,000 integration features. Seurat clusters (resolution of 1.2) were assigned to known cell types by looking at the expression of marker gene signatures from Human Lung v.1 Azimuth reference^83^ and PangaloDB^84^. A focused analysis was performed on the epithelial, mesenchymal, endothelial and immune compartments, which were re-normalized again separately to refine cell type annotation. The labels were then transferred back to the complete anchored and un-anchored datasets. Cell types were annotated with three levels of granularity.

### Iron accumulation signature

To determine the IAS, we performed differential expression analysis per cell type (level 1 annotation) using the FindMarkers function on raw counts. Cells from mice treated with iron 2 d post-exposure were compared to cells from control mice to identify genes indicative of iron accumulation in mouse lung cells. Testing was limited to genes that were detected in a minimum of 10% of cells and showed, on average, at least 1.5-fold difference (log scale). Genes showing significant differences (adjusted *P* value < 0.05) were ranked by log_2_ fold change. Those genes among the top 500 candidates in at least five of the ten major cell types (level 1 annotation) were selected as constituents of the IAS.

### Single-cell RNA-seq analysis of human lung

The ‘Azimuth meta-analysis of human scRNA-seq datasets’, which is a meta-analysis of a series of datasets of healthy and diseased human lungs mapped to the Human Lung v.1 Azimuth reference, was downloaded from CELLxGENE portal. A subset of 100,000 cells were sampled from three studies (‘habermann_2020’, ‘morse_2019’ and ‘reyfman_2019’), containing both IPF and healthy lung samples. Normalized counts were further imputed and smoothed with Magic (v.2.0.3)^82^. The expression of gene signatures was summarized by taking the average Magic score of its constituent genes. Differential expression analysis was performed in whole lung and also per cell type (level 1 annotation) using FindMarkers. Cells from IPF were compared to those from healthy lungs to identify fibrosis response genes in humans. Genes were ranked by the log_2_ average fold change in this comparison and the enrichment of the mouse IAS was assessed with the gene set enrichment analysis preranked method^85^. A *P* value <0.05 was considered significant. In each cell type, IAS-high cells were defined as having an average expression of the iron accumulation signature genes above normal levels in control conditions (>90th percentile). The same strategy was used to define senescence- and CDKN1A-high cells. A β-binomial model was used to assess the association between the percentage of double-positive cells per patient and the fibrotic status, while adjusting for the differences across studies.

### Histological analysis of diabetic nephropathy patients

We performed Perl’s staining of 26 human biopsies with the diagnosis of diabetic nephropathy. Samples were collected between 2010 and 2016. The study was approved by the Bellvitge Hospital Institutional Review Board. All patients signed informed consent. Iron staining was blindly evaluated by an investigator with experience in kidney pathology (J.M.C.). Scoring was performed as follows. Interstitial fibrosis score: 0, absence; 1, 0–1/3 interstitial area; 2, 1/3–2/3 interstitial area; and 3, 2/3–3/3 interstitial area. Glomerular sclerosis % of glomeruli with global sclerosis. Iron staining: 0, absence; 1, 0–10% tubuli; and 2, >10% tubuli. Enrolled patients received no monetary compensation.

### Histological analysis of patients with acute respiratory distress syndrome

An observational study was planned to evaluate the presence of iron in lung samples obtained from autopsies from patients who died with or without lung injury. The protocol was reviewed and approved by Comité de Ética de la Investigación Clínica del Principado de Asturias (ref. 2020/151). Thirteen samples of paraffin-embedded lung tissue were obtained from autopsy material stored at the tissue bank at Hospital Universitario Central de Asturias. Clinical data were retrospectively collected. ARDS was diagnosed in seven cases, using the Kigali modification of Berlin criteria^86^ to allow the inclusion of patients without mechanical ventilation. Lung samples were taken from the lower lobes. After deparaffinization, lung sections were stained with PPB and counterstained with eosin. Two researchers, blinded to clinical data, evaluated the extent of Perl’s stain from zero (no visible staining) to four (presence of massive aggregates).

### MRI of mice

MRI analysis of mice was conducted on a 7.0-T BioSpec 70/30 horizontal animal scanner (Bruker BioSpin) equipped with an actively shielded gradient system (400 mT m^−1^, 12-cm inner diameter). Animals were placed in supine position in a Plexiglas holder with a nose cone for administration of anesthetic gases (1.5% isoflurane in a mixture of 30% O_2_ and 70% CO) and were fixed using a tooth bar and adhesive tape. The 3D localizer scans were used to ensure accurate position of the area of interest at the isocenter of the magnet. To estimate the R2 map, a multi-slice multi-echo sequence was acquired with nine echo times equally spaced from 11 to 99 ms, TR = 3,000 ms, acquisition matrix of 256 × 256, and voxel size of 0.12 × 0.12, slice thickness 0.8 mm. Ten slices were acquired resulting in a field of view of 30 × 30 × 8 mm³. Multiple gradient echo acquisition was performed to obtain R2* maps, with ten echo times equally spaced from 3.5 ms to 48.5 ms, TR = 800 ms and two averages, acquisition matrix = 256 × 256, voxel size of 0.12 × 0.12 mm² and slice thickness 0.8 mm, ten slices, field of view 30 × 30 × 8 mm². R2 and R2* maps were estimated using in-home scripts in MATLAB (MathWorks). itkSNAP software^87^ was used for segmentation and quantification. For each acquisition, the kidney cortex and medulla were manually delineated over the R2 map and overlaid on the R2* map to extract the average R2* values in these areas.

### MRI of renal allograft patients

This study was approved by the Bellvitge Hospital Ethics Committee (date of approval, 5 December 2019, reference no. PR362/19). Renal allograft recipients admitted for kidney biopsy as a standard of care clinical practice (per protocol or by clinical indication) were enrolled in a transversal study design to observe iron accumulation in their kidneys using T2* relaxation MRI. A written informed consent was obtained from all participants. The population characteristics were male and female adult patients (18–80 years old), who have received kidney transplants and were admitted for performing a kidney biopsy. The exclusion criteria were contraindications for MRI (patients with pacemakers, defibrillators, other implanted electronic devices, metallic foreign body in the eye or insulin pumps) or pregnancy. All MRI studies were performed in a 1.5 Teslas scanner (Phillips Achieva d-Stream.Best) with a 32-channel phased-array surface coil. The MRI protocol included a sagittal T2 TSE-weighted image covering the kidney allograft and perpendicular transverse slices acquired with Dixon Quant modified (mDixon Quant), an iron quantification sequence from the MRI vendor with four sets: water, fat, fat suppression and T2* images. Sequences were acquired with respiratory breath-hold and SENSE reconstruction. The mDixon Quant parameters were: six echo times with an initial TR of 5.3 and TE of 0.92, and the shortest repetition time. The matrix was 132 × 117 pixels and the field of view was 400 × 350 mms. The flip angle was 5°. The bandwidth was 2,505.6 kHz. The scan mode was 3D, with 25 slices and a slice thickness of 3.0 mm, with gap 0, number of signal averages of 1 and scan time duration of 5.4 s. Three regions of interest (ROIs) were manually delineated in the renal cortex taking care to avoid areas involved by susceptibility artifacts. They were placed in the anterior, posterolateral and posteromedial parenchyma and copied in the four sets. T2* values were automatically calculated in the three ROIs and averaged to obtain a representative value for the kidney. The ROI main values were introduced in a web page calculator following the Wood method to convert Hz s^−1^ in mol g^−1^ to quantify the iron burden. Kidney allograft biopsy was performed as per clinical practice. The histological sample was evaluated according to the Banff 2017 score^88^. *n* = 20 patients were included and *n* = 13 out of 20 patients were evaluated. Four patients were excluded because another MRI protocol/machine was used, one due to a medical complication (dialysis initiation) and two because of withdrawal of consent. Enrolled patients received no monetary compensation.

### Statistics and reproducibility

The data distribution, sample size (*n*) and statistical significance are all shown in the figures. For animal studies, we used at least three replicates. Data were reproduced in multiple experiments and key findings were replicated in both male and female mice. To estimate sample size, dosage and treatment length, we used pilot experiments. No specific statistical method was used for sample size estimation. Each key in vitro finding was replicated with at least two cell lines, multiple inducers of senescence or iron accumulation, and for each specific condition we included at least two replicates. Statistical analysis was performed using GraphPad Prism. When experimental sample sizes allowed, we tested for Gaussian distribution by performing the Shapiro–Wilk normality test on the datasets. If we detected non-Gaussian distribution, we used the Mann–Whitney *U*-test for comparing two datasets. When the two datasets were derived from paired observations, we used the Wilcoxon matched-pairs signed-rank test. When we performed multiple comparisons we used the Kruskal–Wallis test. When the Shapiro–Wilk test did not detect non-Gaussian distribution, we used an unpaired two-tailed *t*-test for comparing two datasets, or for multiple comparisons, we used a one-way ANOVA. When analyzing histologies of human biopsies, pathologists were blinded to the clinical conditions. When analyzing mouse histologies, automated analysis by QuPath ensured blinding. Data were excluded only when technical error could be identified (for example, when mice died due to the procedure before the end point). Randomization of animals in intervention studies was performed in Excel using the rand function.

### Reporting summary

Further information on research design is available in the Nature Portfolio Reporting Summary linked to this article.

### Supplementary information

Reporting Summary

Supplementary Table 1 **Supplementary Data Table 1. Additional clinical information on patients with DKD**. Clinical information on patients include: sex, age, positivity for Perl’s staining in kidney biopsies (Perl’s+), the score of Perl’s staining (0–3), IFTA score (0–3) glomerular filtration rate (GFR), proteinuria, erythrocyte parameters, including hemoglobin (Hb), mean corpuscular volume (MCV) and mean corpuscular hemoglobin (MCH), serum parameters, including serum transferrin, serum ferritin, serum iron, transferrin saturation, C-reactive protein (CRP), albumin (Alb) and phosphate. We show whether patients received treatment for iron deficiency or transfusions. We highlight comorbidities that may have altered iron homeostasis, including diabetes mellitus, cirrhosis and its grade (1–3) and cardiomyopathy. NA, data are not available. Average values for each parameter for Perl’s-negative and Perl’s-positive groups, and comparison of the averages by two-tailed non-paired Student’s *t*-test is highlighted at the bottom of the columns (parameters showing significant difference are in bold).

Supplementary Table 2 **Supplementary Data Table 2. IAS genes**.

Supplementary Table 3 **Supplementary Data Table 3. Additional clinical information on renal allograft patients**. Clinical information on patients include: sex, age, iron measured in the kidney cortex by MRI, IFTA score (0–3), IFTA group, estimated GFR (eGFR), proteinuria, albuminuria, score of vascular lesions (0–3), score of glomerulitis (0–3), score of tubulitis (0–3), score of interstitial infiltrate (0–3), score of glomerulosclerosis (0–3), erythrocyte parameters, including Hb, MCV and MCH, serum parameters. including serum transferrin, serum ferritin, serum iron, transferrin saturation, CRP, Alb and phosphate. We show whether patients received treatment for iron deficiency or transfusions. We highlight comorbidities that may have altered iron homeostasis, including diabetes mellitus, cirrhosis and its grade (1–3) and cardiomyopathy. NA, data are not available. Average values for each parameter in IFTA absent/mild and IFTA moderate/severe groups and comparison of the averages by two-tailed non-paired Student’s *t*-test is highlighted at the bottom of the columns (parameters showing significant difference are in bold).

Supplementary Table 4 **Supplementary Data Table 4. List of primers**. Primers used to detect targets by qPCR throughout the manuscript. Columns show target species, target and sequence of forward and reverse primers.

### Source data

Source Data Figures All numerical data that appear in the manuscript as graphs. Each tab contains the numerical data of the highlighted figure panel.

Source Data for Extended Data All numerical data that appear in the Extended Data section of the manuscript as graphs. Each tab contains the numerical data of the highlighted figure panel.

Uncropped western blots Uncropped labeled western blots that are shown in the manuscript and their replicates that are used for quantification.

## Figures and Tables

**Extended Data Table 1:**
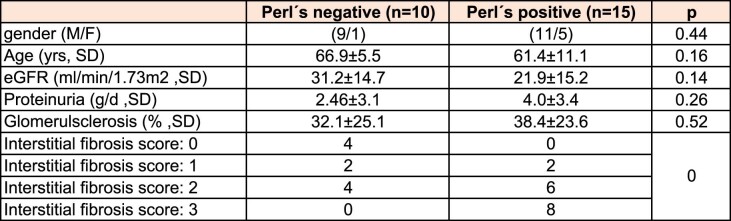
Quantification of Perl’s staining in biopsies from diabetic kidney disease patients

Columns show patients with Perl’s negative and Perl’s-positive kidney biopsies. Rows show characteristics of these patients (mean ± SD or number of patients with specified characteristics). We compared distributions using the Mann–Whitney test.

**Extended Data Table 2:**
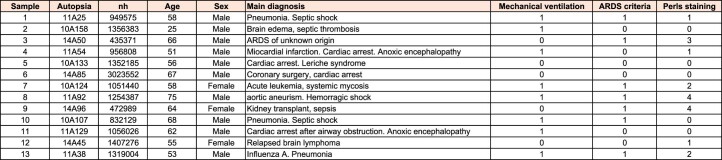
Quantification of Perl’s staining in lung of ARDS patients

Columns show: the age, gender, and anonymized identifier of the individual; the main diagnosis of the patient; whether mechanical ventilation was used (1) or not (0); whether ARDS was diagnosed (1) or not (0); the score of the Perl’s staining in the lung tissue of the patient (0-4).

## Data Availability

Raw reads and count matrices of snRNA-seq data are publicly available at ArrayExpress database under accession no. E-MTAB-13032 and at www.ebi.ac.uk/biostudies/arrayexpress/studies/E-MTAB-13032. Source data are provided with this paper.
