# Supplementary material for: Iron accumulation drives fibrosis, senescence and the senescence-associated secretory phenotype
Source: Nat Metab. 2023 Dec 14;5(12):2111–30. doi: 10.1038/s42255-023-00928-2 (PMC10730403; doi:10.1038/s42255-023-00928-2)
Supplement: Supplementary file 1 — Reporting Summary [file 42255_2023_928_MOESM1_ESM.pdf]

nature portfolio

Corresponding author(s): Mate Maus and Manuel Serrano

Last updated by author(s): Sep 4, 2023

Reporting Summary

Nature Portfolio wishes to improve the reproducibility of the work that we publish. This form provides structure for consistency and transparency in reporting. For further information on Nature Portfolio policies, see our Editorial Policies and the Editorial Policy Checklist.

Statistics

For all statistical analyses, confirm that the following items are present in the figure legend, table legend, main text, or Methods section.

n/a	Confirmed	
☐	☒	The exact sample size (*n*) for each experimental group/condition, given as a discrete number and unit of measurement
☐	☒	A statement on whether measurements were taken from distinct samples or whether the same sample was measured repeatedly
☐	☒	The statistical test(s) used AND whether they are one- or two-sided<br>*Only common tests should be described solely by name; describe more complex techniques in the Methods section.*
☐	☒	A description of all covariates tested
☐	☒	A description of any assumptions or corrections, such as tests of normality and adjustment for multiple comparisons
☐	☒	A full description of the statistical parameters including central tendency (e.g. means) or other basic estimates (e.g. regression coefficient) AND variation (e.g. standard deviation) or associated estimates of uncertainty (e.g. confidence intervals)
☐	☒	For null hypothesis testing, the test statistic (e.g. *F*, *t*, *r*) with confidence intervals, effect sizes, degrees of freedom and *P* value noted<br>*Give P values as exact values whenever suitable.*
☒	☐	For Bayesian analysis, information on the choice of priors and Markov chain Monte Carlo settings
☒	☐	For hierarchical and complex designs, identification of the appropriate level for tests and full reporting of outcomes
☒	☐	Estimates of effect sizes (e.g. Cohen's *d*, Pearson's *r*), indicating how they were calculated

Our web collection on statistics for biologists contains articles on many of the points above.

Software and code

Policy information about availability of computer code

Data collection	Flow cytometry: Gallios flow cytometer (Beckman Coulter)<br>IHC: NanoZoomer-2.0 HT C9600 digital scanner
Data analysis	QuPath, GraphPad Prism 8, FlowJo (10.0.7)

For manuscripts utilizing custom algorithms or software that are central to the research but not yet described in published literature, software must be made available to editors and reviewers. We strongly encourage code deposition in a community repository (e.g. GitHub). See the Nature Portfolio guidelines for submitting code & software for further information.

Data

Policy information about availability of data

All manuscripts must include a data availability statement. This statement should provide the following information, where applicable:

- Accession codes, unique identifiers, or web links for publicly available datasets
- A description of any restrictions on data availability
- For clinical datasets or third party data, please ensure that the statement adheres to our policy

"Raw reads and count matrices of snRNA-seq data are publicly available at ArrayExpress database under accession number E-MTAB-13032 and at the following URL: https://www.ebi.ac.uk/biostudies/arrayexpress/studies/E-MTAB-13032." - this data availability statement is part of the manuscript.

Human research participants

Policy information about studies involving human research participants and Sex and Gender in Research.

Reporting on sex and gender	Both genders were included in the study.
Population characteristics	Study population: Renal allograft recipients admitted to perform kidney biopsy as standard of care clinical practice (per protocol or by clinical indication). Clinical information and patient covariant relevant population statistics is listed in Extended Data Table 4.
Recruitment	All kidney transplant recipients admitted to perform a kidney graft biopsy were prospectively recruited from February 2020 to October 2021 if they fulfilled the inclusion criteria and did not fall into the exclusion criteria.<br><br>Inclusion criteria:<br>● Written informed consent<br>● Age 18-80 year<br>● Kidney transplant recipient admitted to perform a kidney biopsy<br>Exclusion criteria:<br>● Contraindications for MRI. Patients with pacemakers, defibrillators, other implanted electronic devices, metallic foreign body in the eye, insulin pumps.<br>● Pregnancy<br>● Absence of informed consent
Ethics oversight	For the MRI analysis of renal iron accumulation, the study was approved by the Bellvitge Hospital IRB. The protocol for the autopsy study of ARDS patients was reviewed and approved by the Comité de Ética de la Investigación Clínica del Principado de Asturias (ref 2020/151).

Note that full information on the approval of the study protocol must also be provided in the manuscript.

Field-specific reporting

Please select the one below that is the best fit for your research. If you are not sure, read the appropriate sections before making your selection.

☒ Life sciences ☐ Behavioural & social sciences ☐ Ecological, evolutionary & environmental sciences

For a reference copy of the document with all sections, see nature.com/documents/nr-reporting-summary-flat.pdf

Life sciences study design

All studies must disclose on these points even when the disclosure is negative.

Sample size	No statistical methods were used to predetermine sample size estimates. For in vitro experiments sample size was determined based on preliminary experiments. For in vivo experiments, sample size was determined based on previous experiments and small pilot studies.
Data exclusions	Data was excluded only when technical error could be identified (eg., when mice died due to the procedure before endpoint).
Replication	All in vitro experiments were successfully replicated at least in three different experiments. In vivo studies have been performed as follows. Intratracheal iron delivery, bleomycin induced lung fibrosis and folic acid induced kidney fibrosis have been performed over five times each. Replicates included different readouts. Ureteral obstruction model was performed once. Coronary artery ligation for induction of ischemic heart disease was performed two times. MRI study of mice was performed two times. We successfully replicated the study results.
Randomization	Randomization of animals in intervention studies was performed in Excel using the rand function.
Blinding	Quantification of IHC were performed either by an algorithm, or in a blinded fashion when done by human evaluators. In vitro experiments were performed in a non-blinded fashion, instead two different authors separately replicated different aspects of the same biology.

Reporting for specific materials, systems and methods

We require information from authors about some types of materials, experimental systems and methods used in many studies. Here, indicate whether each material, system or method listed is relevant to your study. If you are not sure if a list item applies to your research, read the appropriate section before selecting a response.

Materials & experimental systems

n/a	Involved in the study	
☐	☒	Antibodies
☐	☒	Eukaryotic cell lines
☒	☐	Palaeontology and archaeology
☐	☒	Animals and other organisms
☐	☒	Clinical data
☒	☐	Dual use research of concern

Methods

n/a	Involved in the study	
☒	☐	ChIP-seq
☐	☒	Flow cytometry
☒	☐	MRI-based neuroimaging

Antibodies

Antibodies used

BRDU staining
BRDU antibody (Santa Cruz Biotech, sc-32323)
goat anti-mouse IgG (H+L) Alexa Fluor™ 488 (Thermo Fisher, A-11001)

Flow cytometry analysis of cell viability
Annexin-V and Propidium Iodide [PI] (Thermo Fisher, 88-8007-74)
anti-CD45-PE (BD, 561087)
anti-CD45-APC (BD, 559864)
anti-Ly6G-PE (BD, 551461)
anti-CD11b-PE/Cy7 (Biolegend, 101215)
anti-CD11b-Pacific Blue (Biolegend, 101223)
anti-F4/80-APC (Biolegend, 123115)

Immunohistochemistry
p21 clone HUGO 291H/B5 (CNIO)
p21 WAF1/Cip1 SX118 (Dako-Agilent, M7202)
NE (Abcam, ab68672)
F4/80 D2S9R (CellSignalling, 70076)
alpha SMA [1A4] (Abcam , ab7817)
HMOX1 (Abcam, ab13243)
TER-119 (Stem Cell, 60033)
OmniMap anti-Rat HRP (Roche, 760-4457)
OmniMap™ anti-Rb HRP (Roche, 760-4311).
IgG1+IgG2a+IgG3 [M204-3] (Abcam, ab133469) ad
BrightVision Poly-HRP-Anti Rabbit IgG Biotin-free, ready to use (DPVR-110 HRP, Immunologic)
HRP-Anti-Rat IgG (MP-7444, Vector)
Goat Anti-Mouse Immunoglobulins/HRP (Dako-Agilent, P0447)
rabbit IgG, polyclonal (Abcam, ab27478)
mouse IgG1, Kappa (NCG01) (Abcam, ab81032)
mouse IgG2a kappa (eBM2a) (eBioscience™, 14-4724-82)
rat IgG (R&D Systems, 6- 001-F)

Western blotting
nti-FTH1 (3998S, Cell Signaling)
anti-β actin (A5441, Sigma)
anti-CD71/TfR (Cell Signaling, D7S5Z, 1:1000)
anti-ZIP14 (PA5-21077, Thermo Fisher, 1:1000)
anti-GAPDH (ab9485, Abcam, 1:2000)
anti-IgG antibodies anti-rabbit IRDye 680 CW (1:15,000, LI-COR, #926-68071)
anti-rabbit IRDye 800 CW (1:15,000, LI-COR, #926-32211)
anti-goat IRDye 680 CW (1:10,000, LI-COR, #926-68074)

Validation

Antibodies have been validated by the manufacturer/ source. Data are available on the manufacturer website.

Eukaryotic cell lines

Policy information about cell lines and Sex and Gender in Research

Cell line source(s)

Cell lines SK-MEL-103 (human melanoma), H5V (mouse endothelial cells), HUVEC (human umbilical vein endothelial cells), IMR90 (human fetal lung fibroblast) were obtained from ATCC. Mouse embryonic fibroblasts (MEF) were isolated as described(1).

Authentication

None of the cell lines have been authenticated.

Mycoplasma contamination

All cell lines used in the experiments shown in this study were tested for Mycoplasma contamination by PCR and were found negative.

Commonly misidentified lines
(See ICLAC register)

No misidentified cell lines were used in this study.

Animals and other research organisms

Policy information about studies involving animals; ARRIVE guidelines recommended for reporting animal research, and Sex and Gender in Research

Laboratory animals	All mice were housed under specific pathogen free (SPF) in individual ventilated cages (IVC) in a controlled environment room (temperature 20-24ºC, relative humidity 30-70%, and positive pressure) under a 12 h-12 h light-dark cycle and allowed unrestricted access to food and water. Wild-type and Tie2-Cre-ERT2 Rosa26-iDTR in the Parc Cientific de Barcelona (PCB) Animal Facility, with registration number B-9900044. The environmental parameters were controlled using the software Controlli Delta Spain (version 3.0). Protocols were approved by the Animal Care and Use Ethical Committee of animal experimentation of the Barcelona Science Park (CEEA-PCB) and the Catalan Government. TGAbrd1 mice, overexpressing the human ß1-adrenoceptor in cardiac myocytes driven by the α-myosin heavy chain promoter, develop spontaneous cardiomyopathy, mimicking human heart failure, were described earlier71. The TGADRB1 mice on Friend Virus B NIH (FVB/N) background were housed in the Institute of Pharmacology and Toxicology at the Technical University of Munich, Germany. Male transgenic mice and WT littermates were analyzed at the age of 2.5, 5, and 10 to 12 months. Tie2-Cre-ERT2 Rosa26-iDTR mice were generated by crossing Tie2-Cre-ERT2 mice (kindly donated by Dr. Sagrario Ortega, to be described in a future paper) to Rosa26-iDTR mice72. For all other studies, we used 8-10 weeks old C57BL/6 mice. Where female mice were used are specifically indiciated. Euthanasia of animals was performed by CO2 or by cervical dislocation.
Wild animals	This study did not involve wild animals.
Reporting on sex	The gender of the mice used in the different experiment is highlighted in each cases in the figure legends.
Field-collected samples	No field-collected samples were used in this study.
Ethics oversight	Mice were housed under specific pathogen free conditions in the mouse facility of the Institute for Research in Biomedicine (IRB) in accordance with the protocols approved by the Animal Care and Use Ethical Committee of animal experimentation of the Barcelona Science Park (CEEA-PCB) and the Catalan Government.

Note that full information on the approval of the study protocol must also be provided in the manuscript.

Clinical data

Policy information about clinical studies
All manuscripts should comply with the ICMJE guidelines for publication of clinical research and a completed CONSORT checklist must be included with all submissions.

Clinical trial registration	*Provide the trial registration number from ClinicalTrials.gov or an equivalent agency.*
Study protocol	*Note where the full trial protocol can be accessed OR if not available, explain why.*
Data collection	*Describe the settings and locales of data collection, noting the time periods of recruitment and data collection.*
Outcomes	*Describe how you pre-defined primary and secondary outcome measures and how you assessed these measures.*

Flow Cytometry

Plots

Confirm that:

☒ The axis labels state the marker and fluorochrome used (e.g. CD4-FITC).

☒ The axis scales are clearly visible. Include numbers along axes only for bottom left plot of group (a 'group' is an analysis of identical markers).

☒ All plots are contour plots with outliers or pseudocolor plots.

☒ A numerical value for number of cells or percentage (with statistics) is provided.

Methodology

Sample preparation

Flow cytometry analysis of cell viability
We induced senescence in SK-MEL-103 cells with doxorubicin (200 nM) or palbociclib (1 μM) and 7 days later, cultured them in the presence of deferiprone (200 μM) or vehicle (water) for 4 days. Cells were stained with an Annexin-V and Propidium Iodide [PI] (Thermo Fisher, 88-8007-74) apoptosis detection kit according to manufacture instructions, and analyzed by flow-cytometry.

Flow-cytometric analysis of lungs
For analyzing lung infiltration, ex-vivo, lungs were intubated through the trachea with 2 ml dispase solution (5U/ml), and then agitated at 37oC. We chopped the lungs with fine scissors and transferred them into a solution of 1%BSA + 60 units/ml

DNase1 + 70 units/ml Collagenase type 1 dissolved in PBS. To generate single-cell suspension, we used the GentleMACS system (Miltenyi) according to the manufacturer’s instructions. Red blood cells were removed in ammonium-chloride-potassium lysis buffer, and debris by using cell strainers. Cells were suspended in an ice-cold PBS with 1% FBS, before blocking with purified rat anti-mouse CD16/CD32 (BD). Surface antigens were stained with fluorescently conjugated antibodies: anti-CD45-PE, anti-CD45-APC, anti-CD11b-V450, anti-CD11b-PE/Cy7 and anti-Ly6G-PE all from BD, and F4/80-APC from Biolegend. Samples were acquired on a Gallios flow-cytometer (Beckman Coulter), acquired data was analyzed with a FlowJo software (Tree Star).

Labile iron measurement
We measured labile iron by flow-cytometry using the FerroOrange Dye (F374, Dojindo) according to the manufacturer's instructions.

Total ROS measurement
Total ROS levels were measured by flow-cytometry using a total ROS detection kit (ENZ-51011, Enzo) according to the manufacturer's instructions.

Lysosomal mass measurement
Lysosomal mass was assessed by flow-cytometry using a LysoTracker dye (L12492, Invitrogen) according to the manufacturer's instructions.

Instrument: Gallios flow cytometer (Beckman Coulter)

Software: FlowJo (10.0.7.)

Cell population abundance: No cell sorting has been performed.

Gating strategy: For tissue cultures: Cells > single cells > living cells (DAPI-)

☒ Tick this box to confirm that a figure exemplifying the gating strategy is provided in the Supplementary Information.
