# Supplementary material for: Iron accumulation drives fibrosis, senescence and the senescence-associated secretory phenotype
Source: Nat Metab. 2023 Dec 14;5(12):2111–30. doi: 10.1038/s42255-023-00928-2 (PMC10730403; doi:10.1038/s42255-023-00928-2)

Figure 5b
Replicate 1,2
WB: FTH1

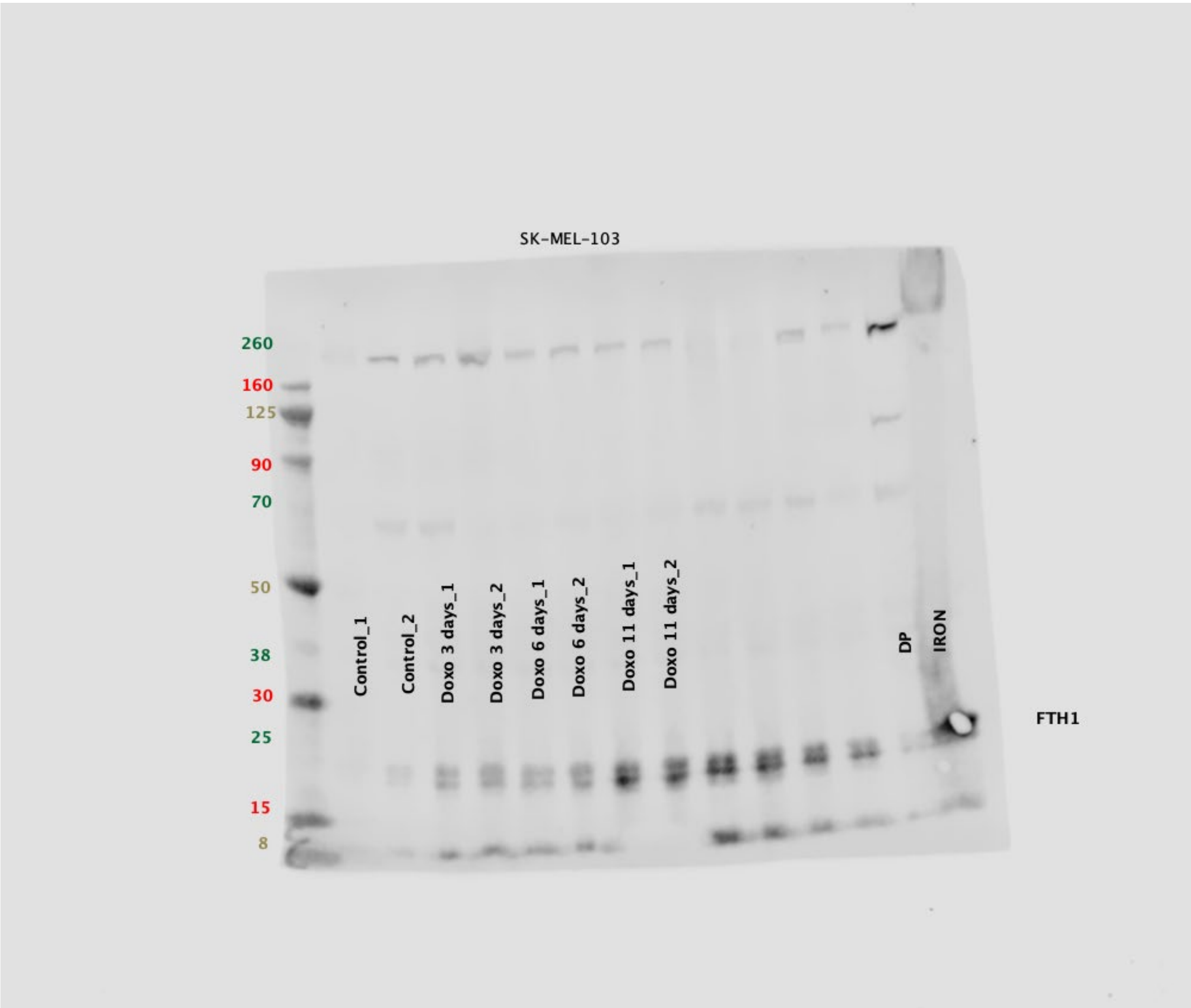

Figure 5b
Replicate 1,2
WB: ACTIN

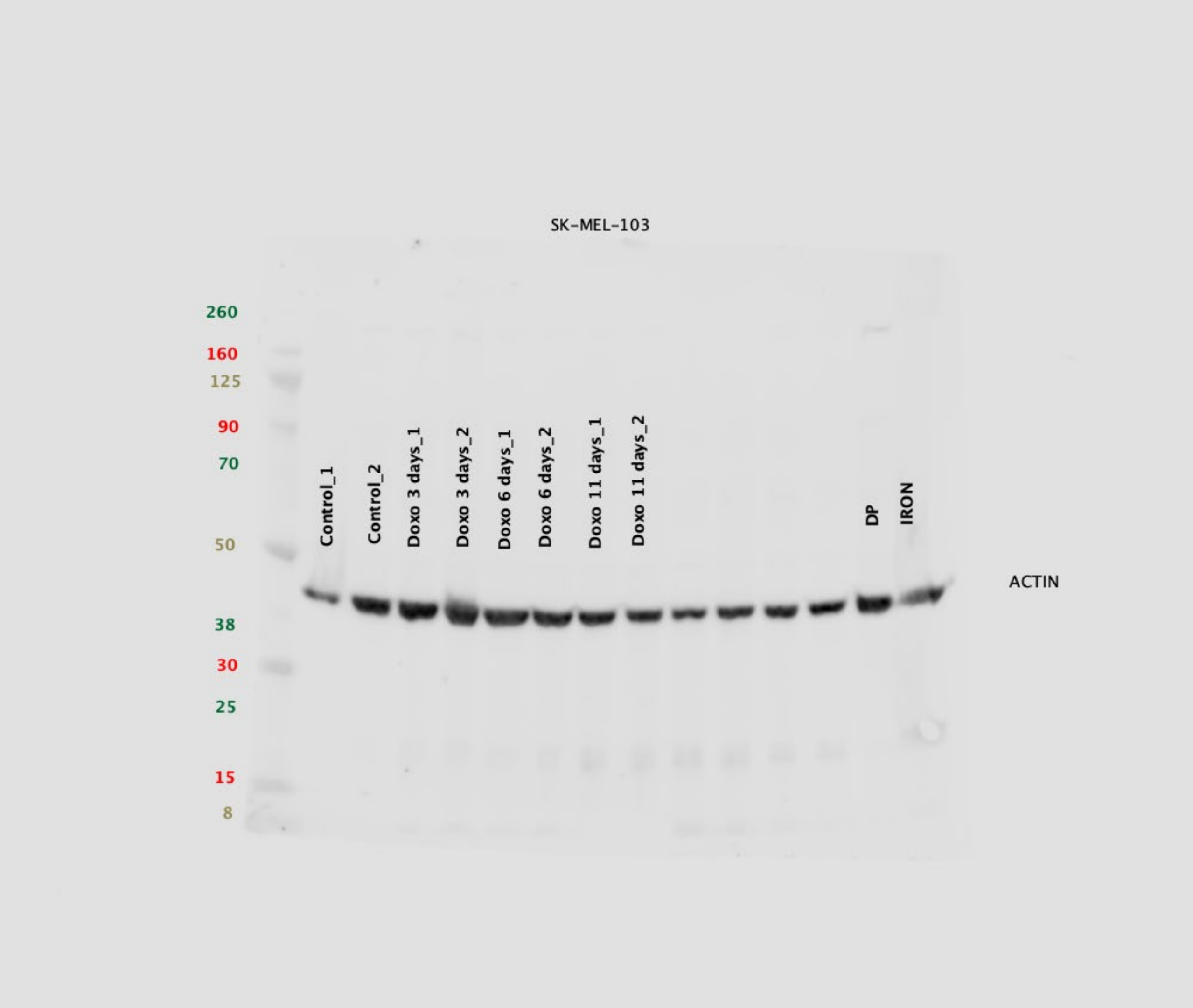

Figure 5c
Replicate 1
WB: FTH1

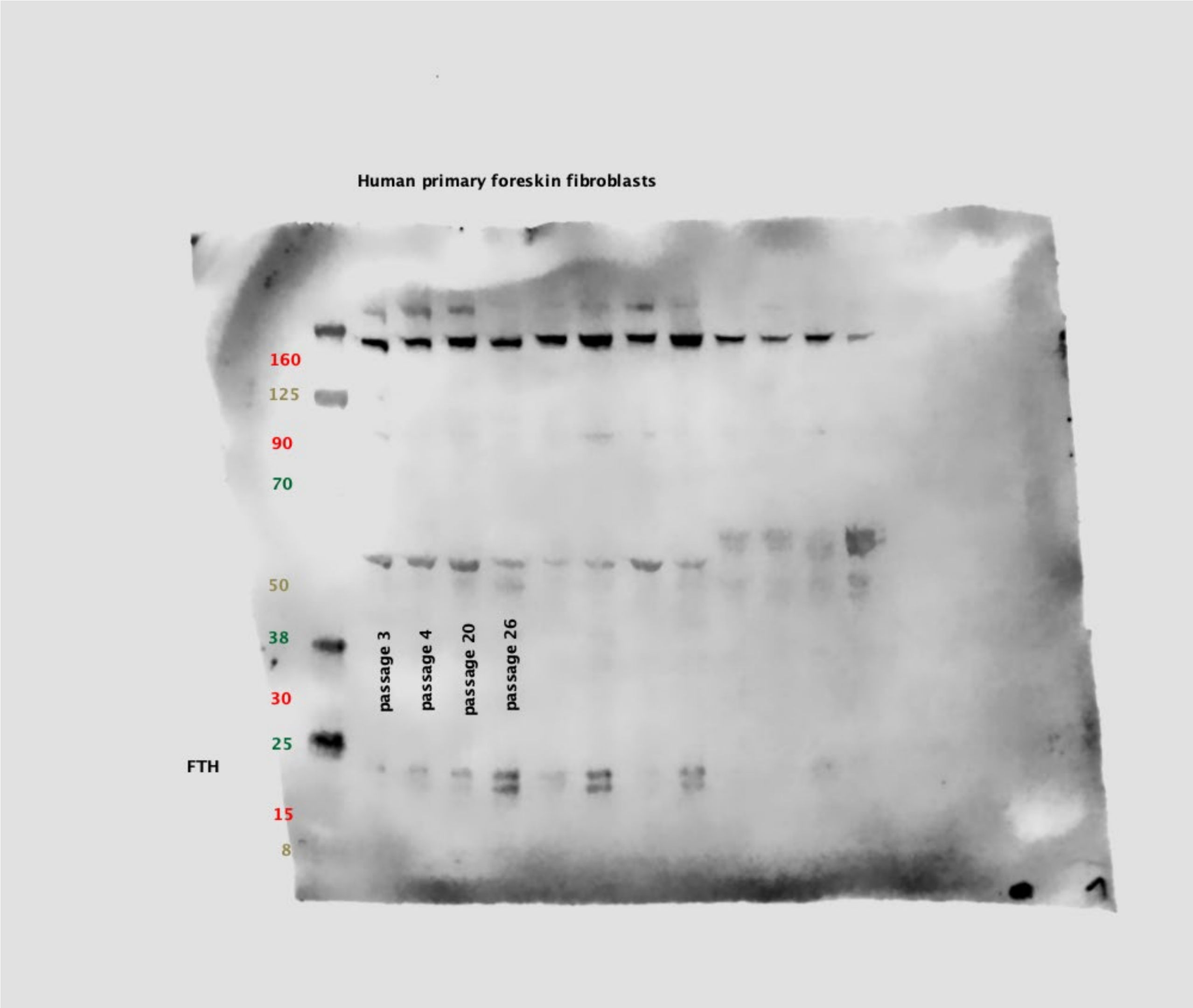

Figure 5c
Replicate 1
WB: ACTIN

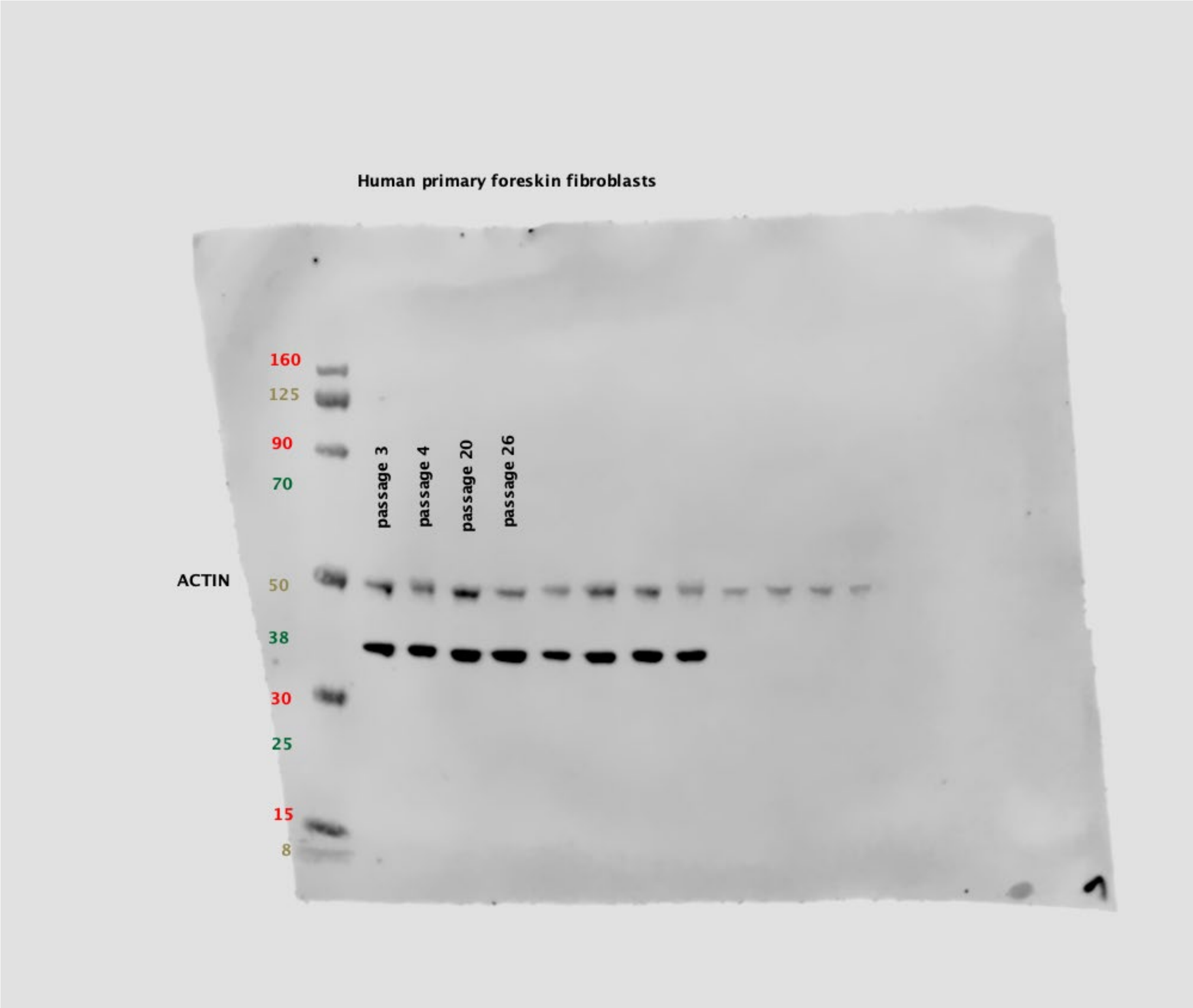

Figure 5e
Replicate 1,2,3
WB: CD71/TfR

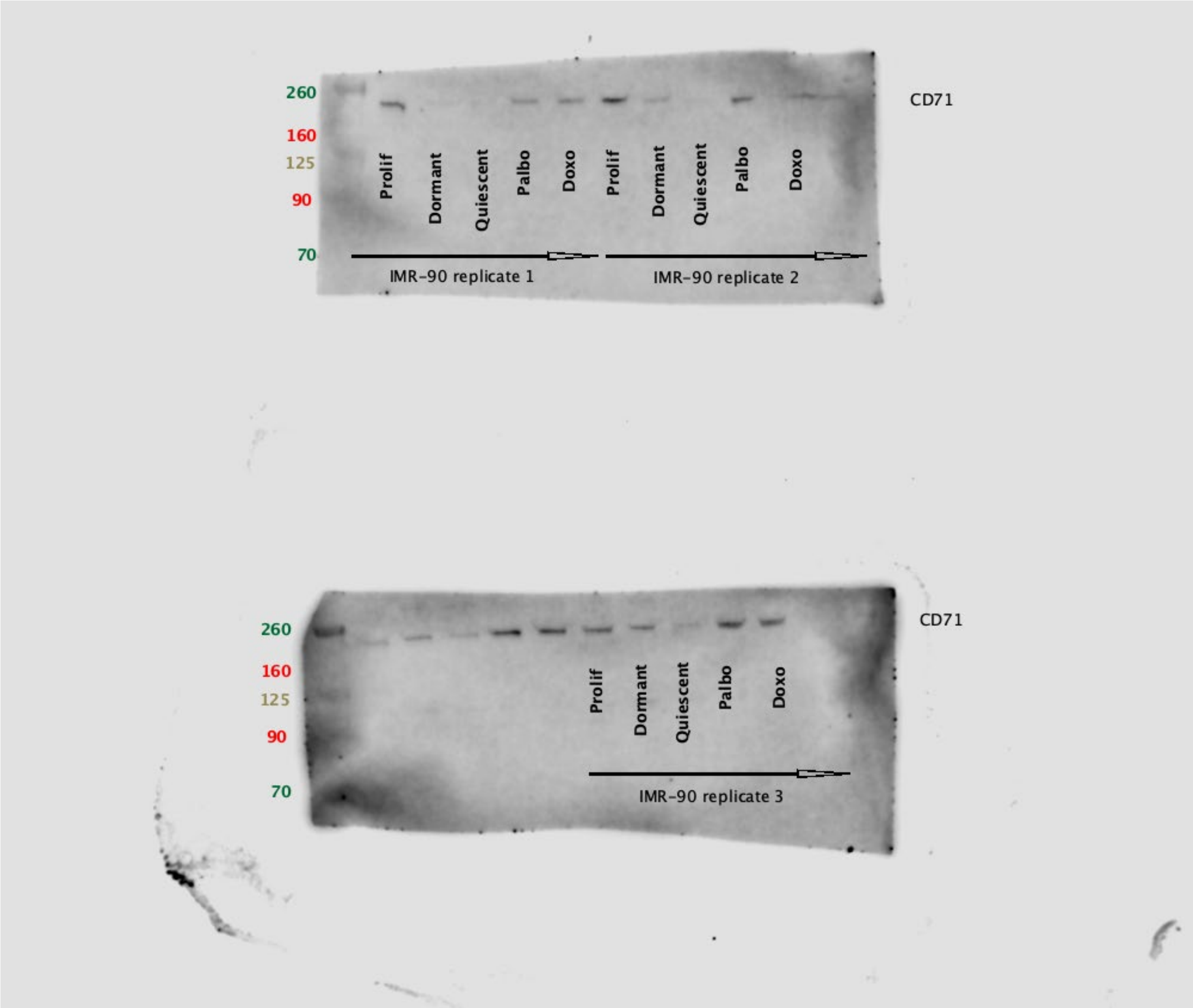

Figure 5e

Replicate 1,2,3

WB: ACTIN

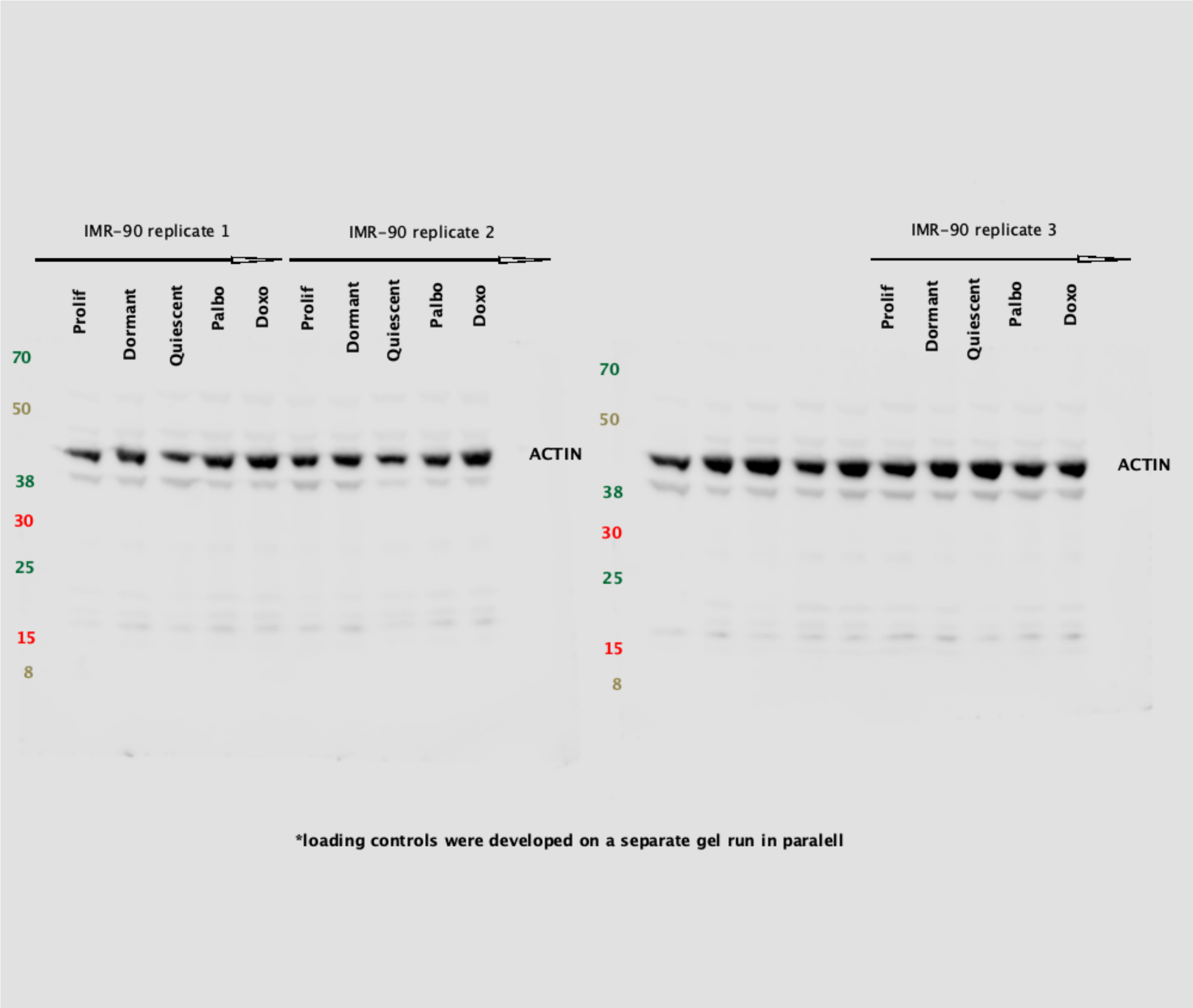

Figure 5f
Replicate 1,2
WB: CD71/TfR

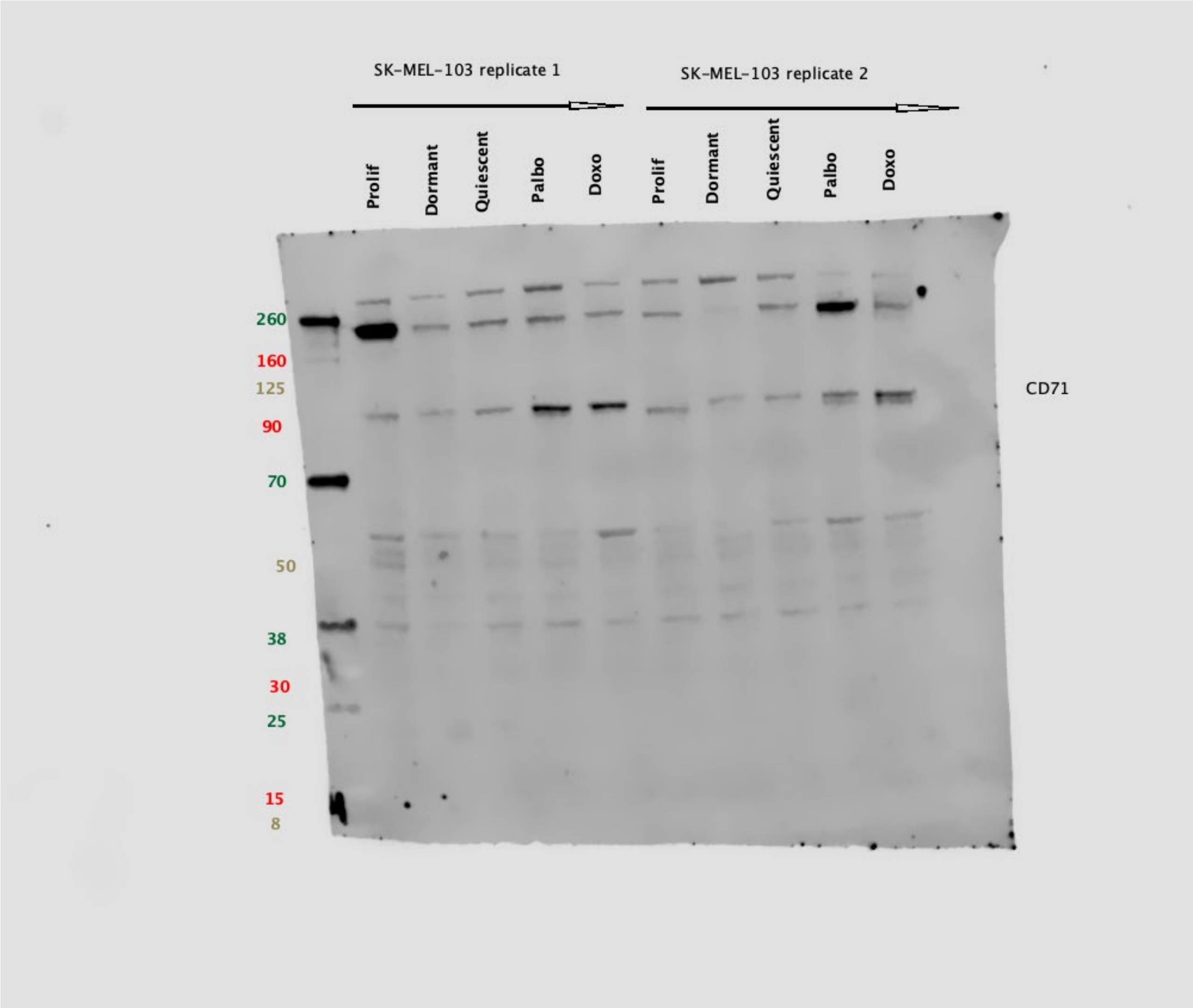

Figure 5f
Replicate 1,2
WB: GAPDH

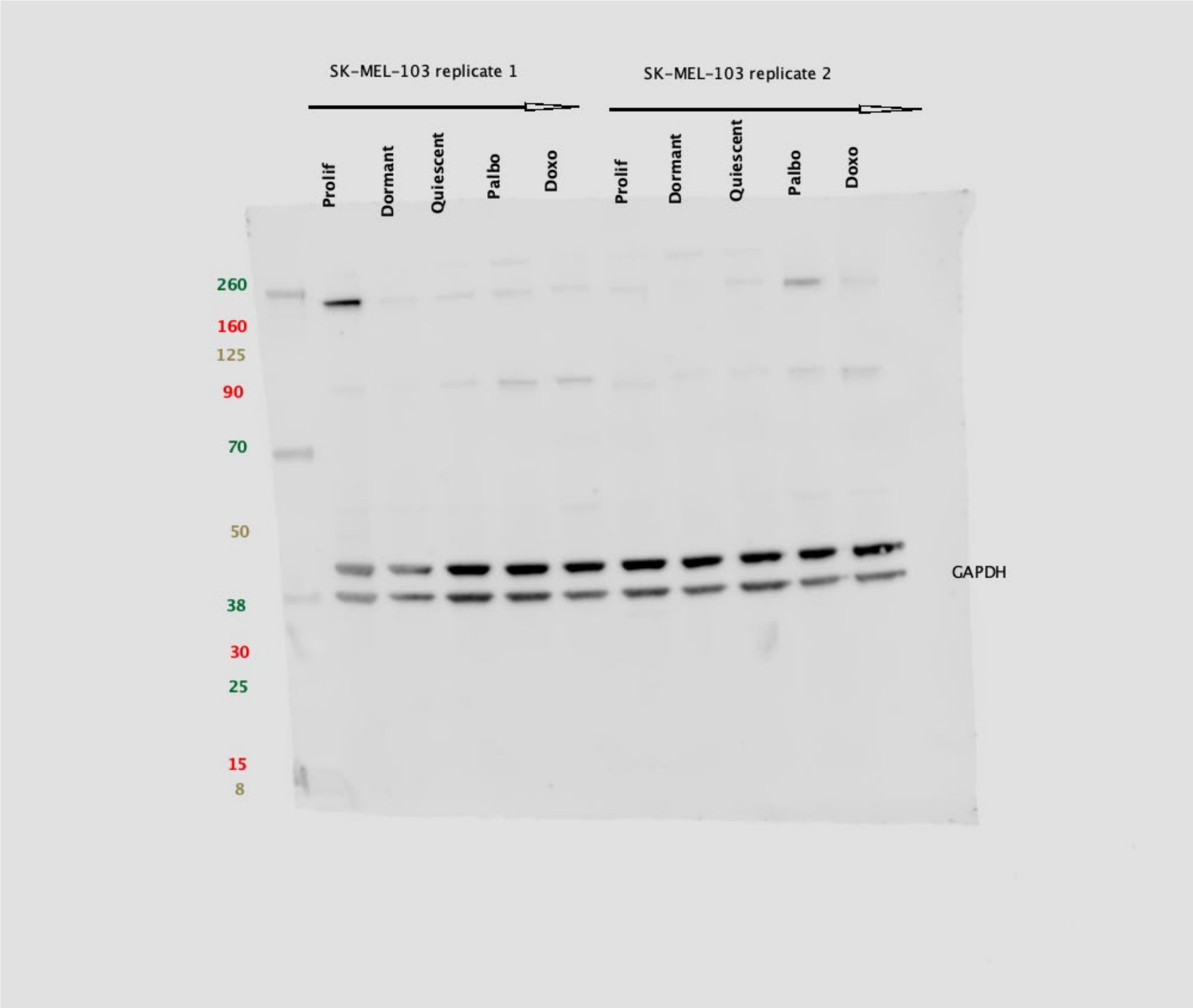

Figure 5f
Replicate 3
WB: CD71/TfR

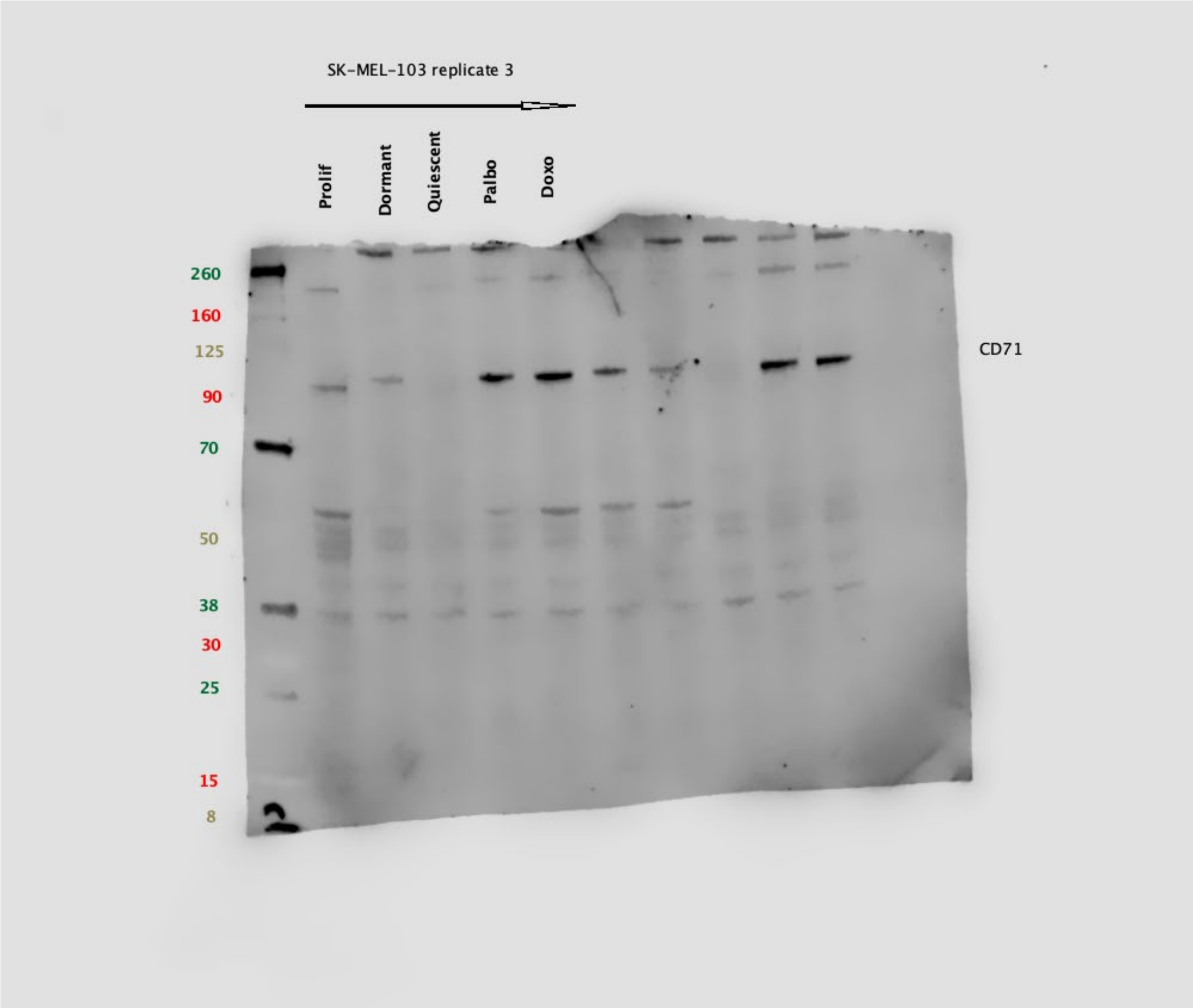

Figure 5f
Replicate 3
WB: GAPDH

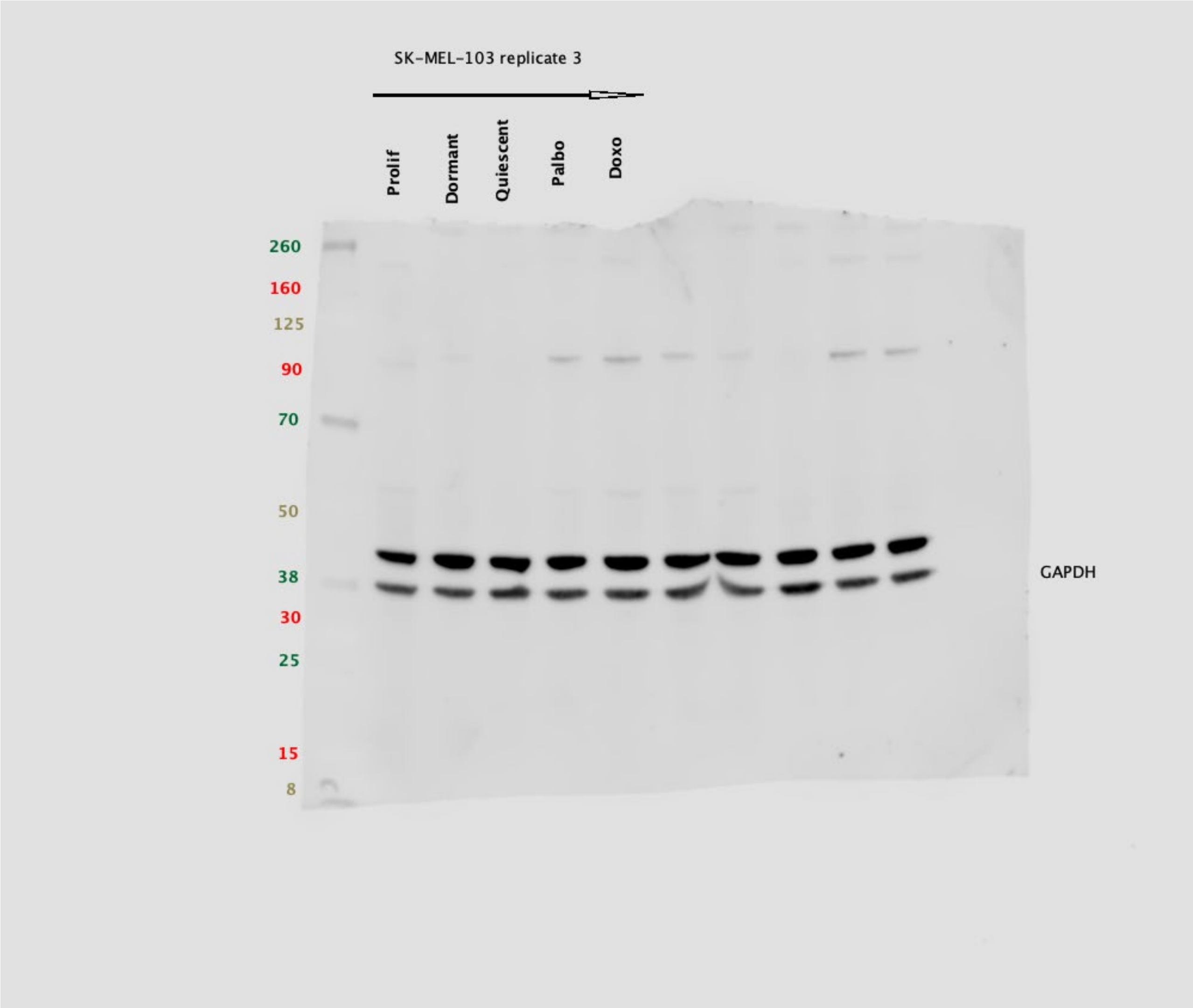

Extended Data Figure 5a
Replicate 1,2
WB: FTH1

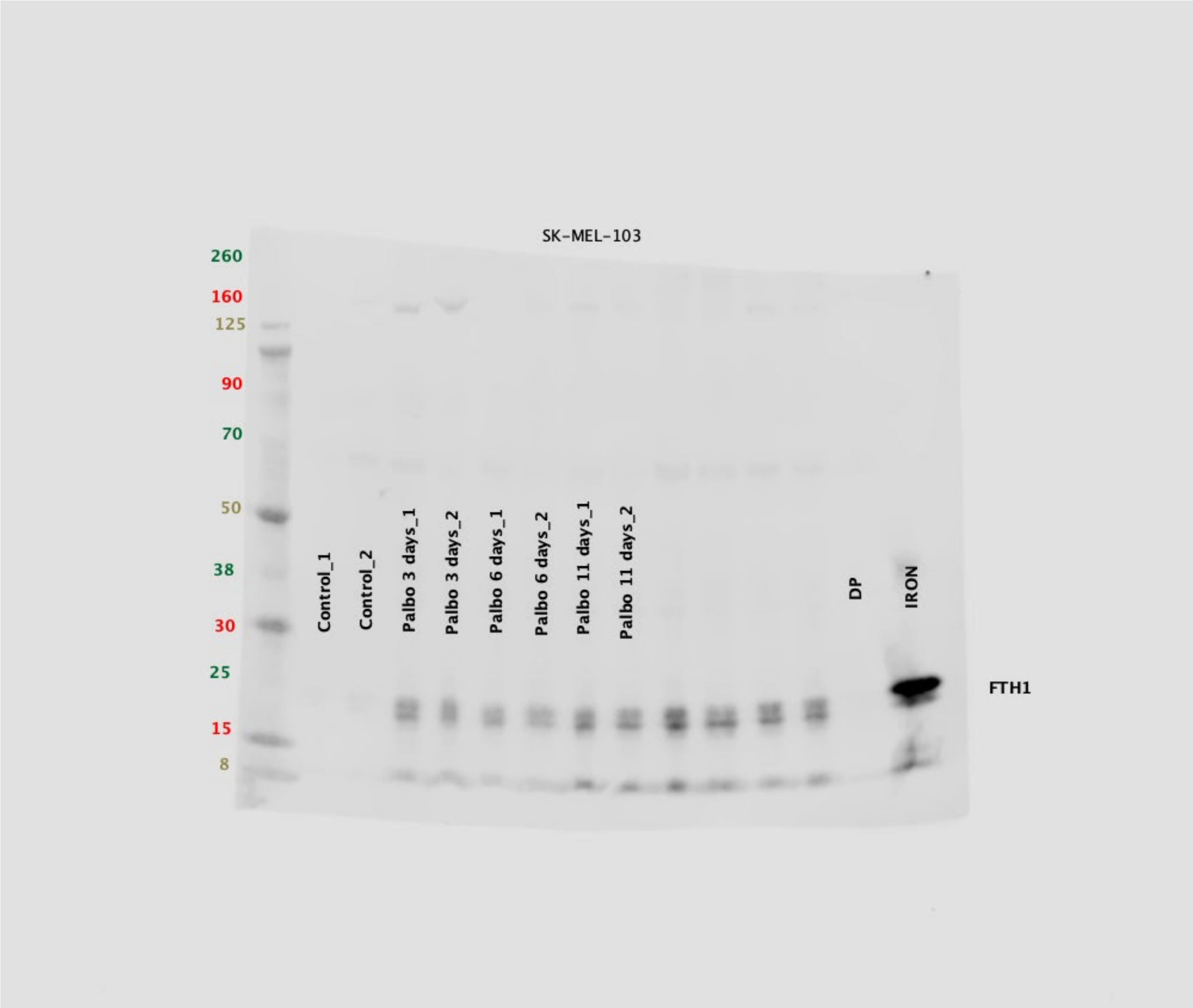

Extended Data Figure 5a

Replicate 1,2

WB: ACTIN

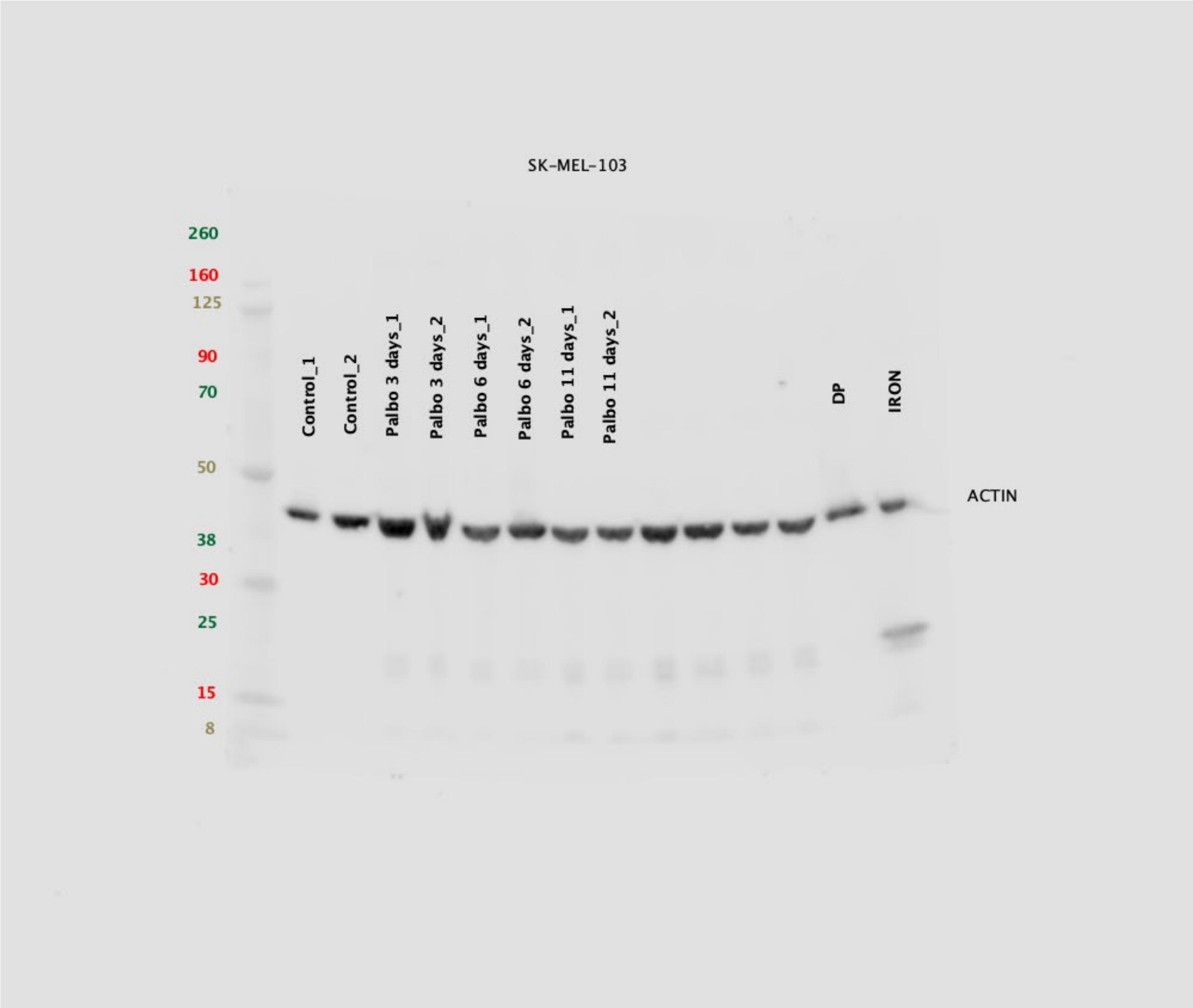

Extended Data Figure 5b
Replicate 1,2
WB: FTH1

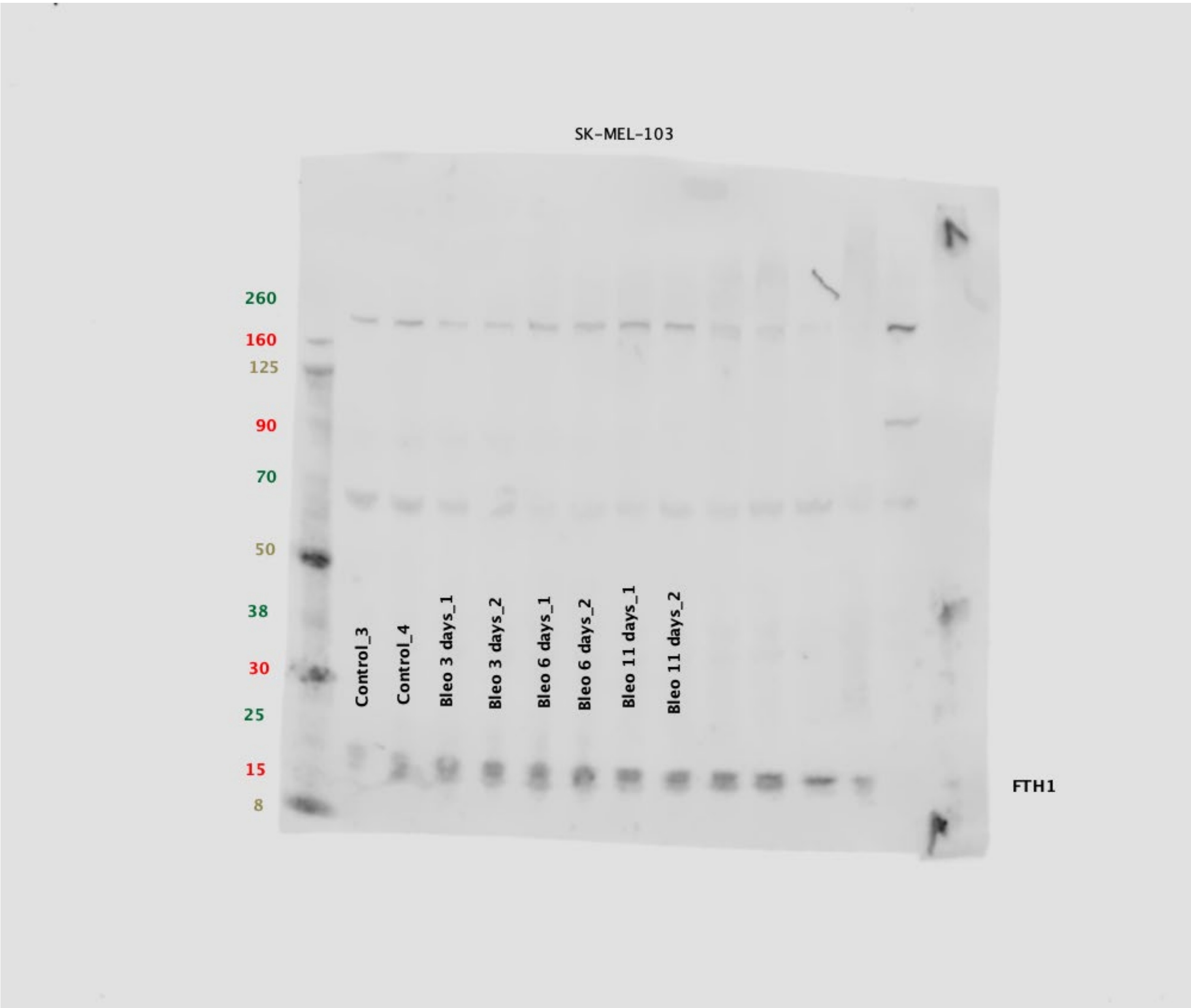

Extended Data Figure 5b

Replicate 1,2

WB: ACTIN

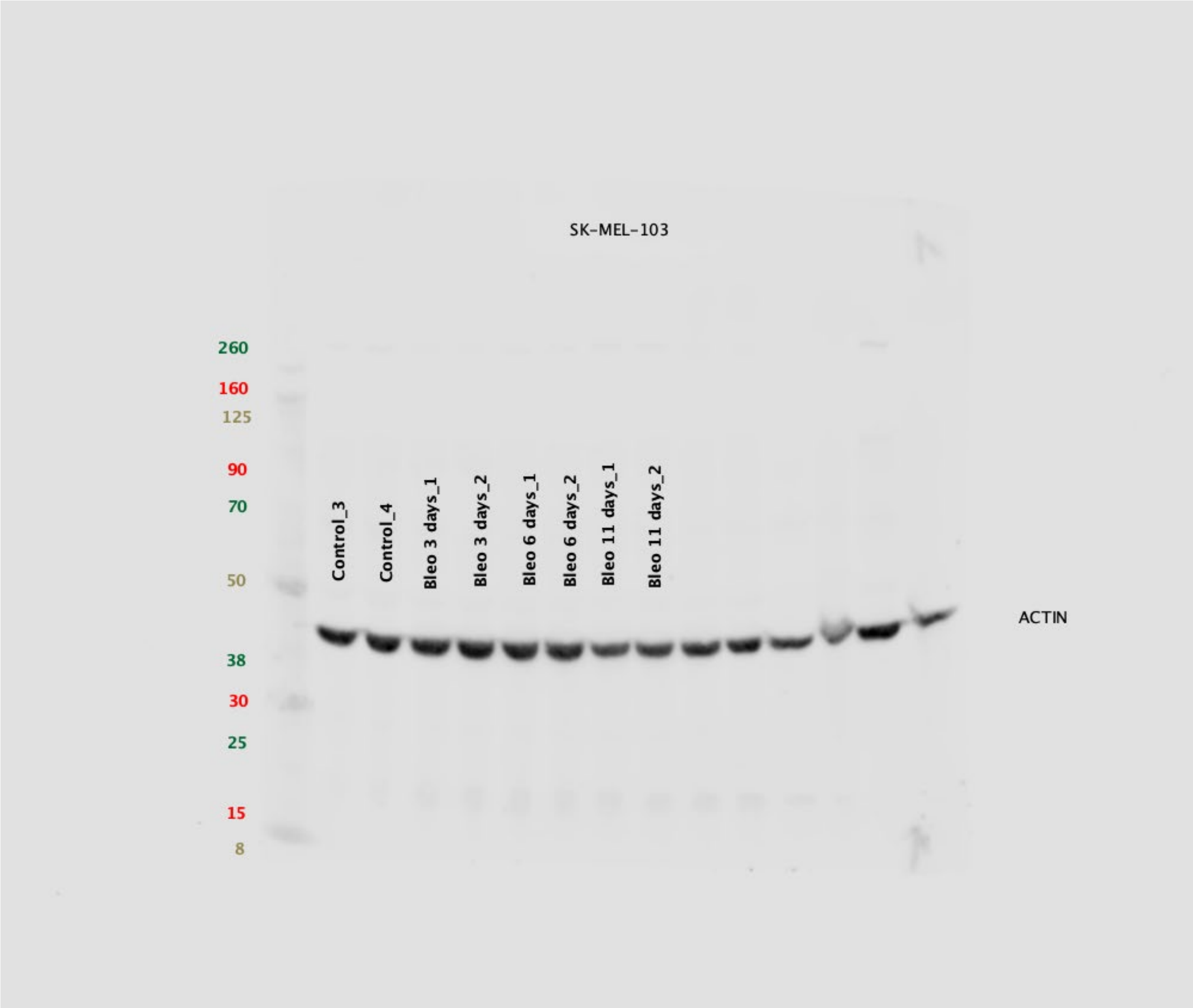

Extended Data Figure 5c
Replicate 1,2
WB: ZIP14

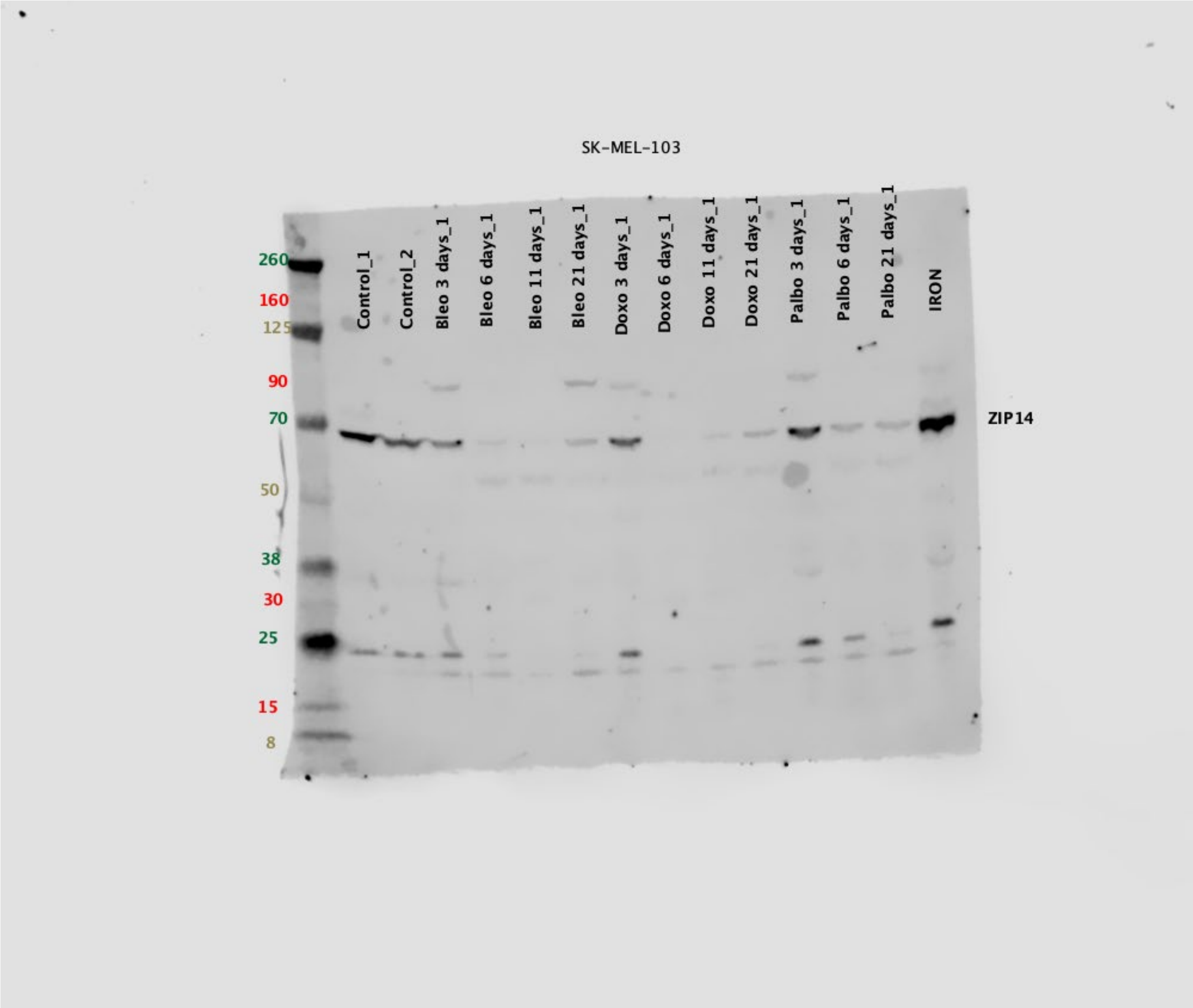

Extended Data Figure 5c
Replicate 1,2
WB: ACTIN

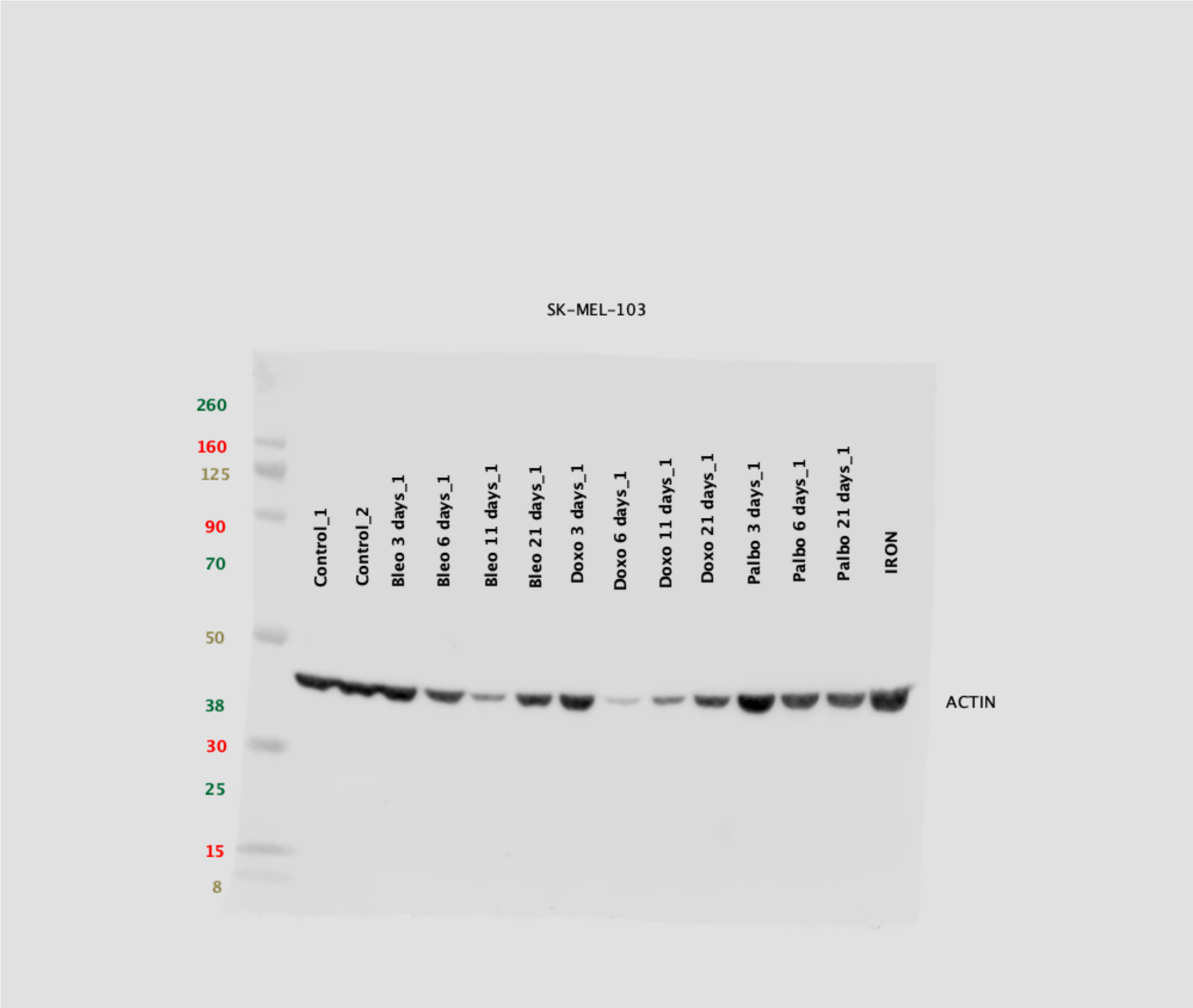

Extended Data Figure 5c
Replicate 2
WB: ZIP14

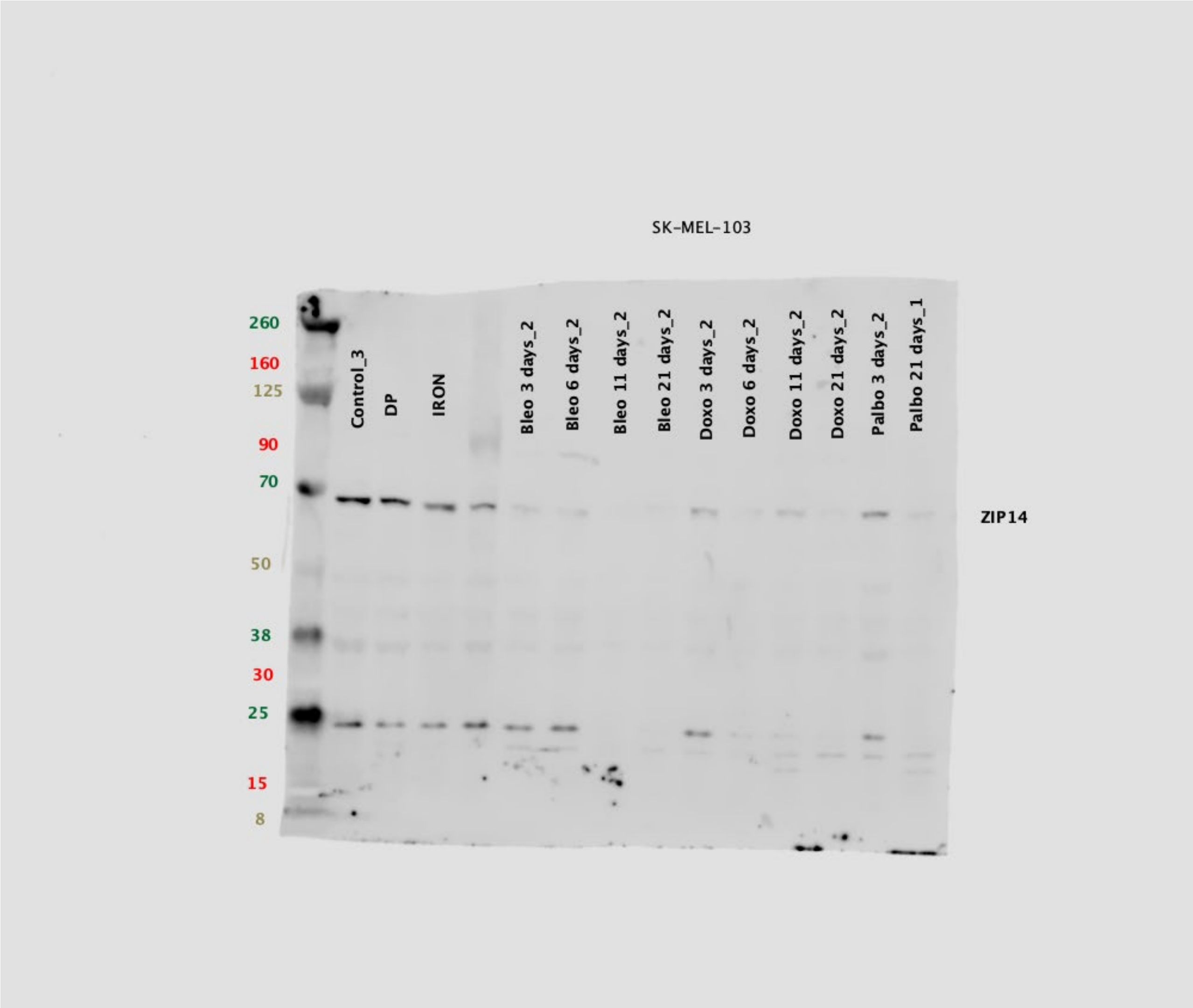

Extended Data Figure 5c
Replicate 2
WB: ACTIN

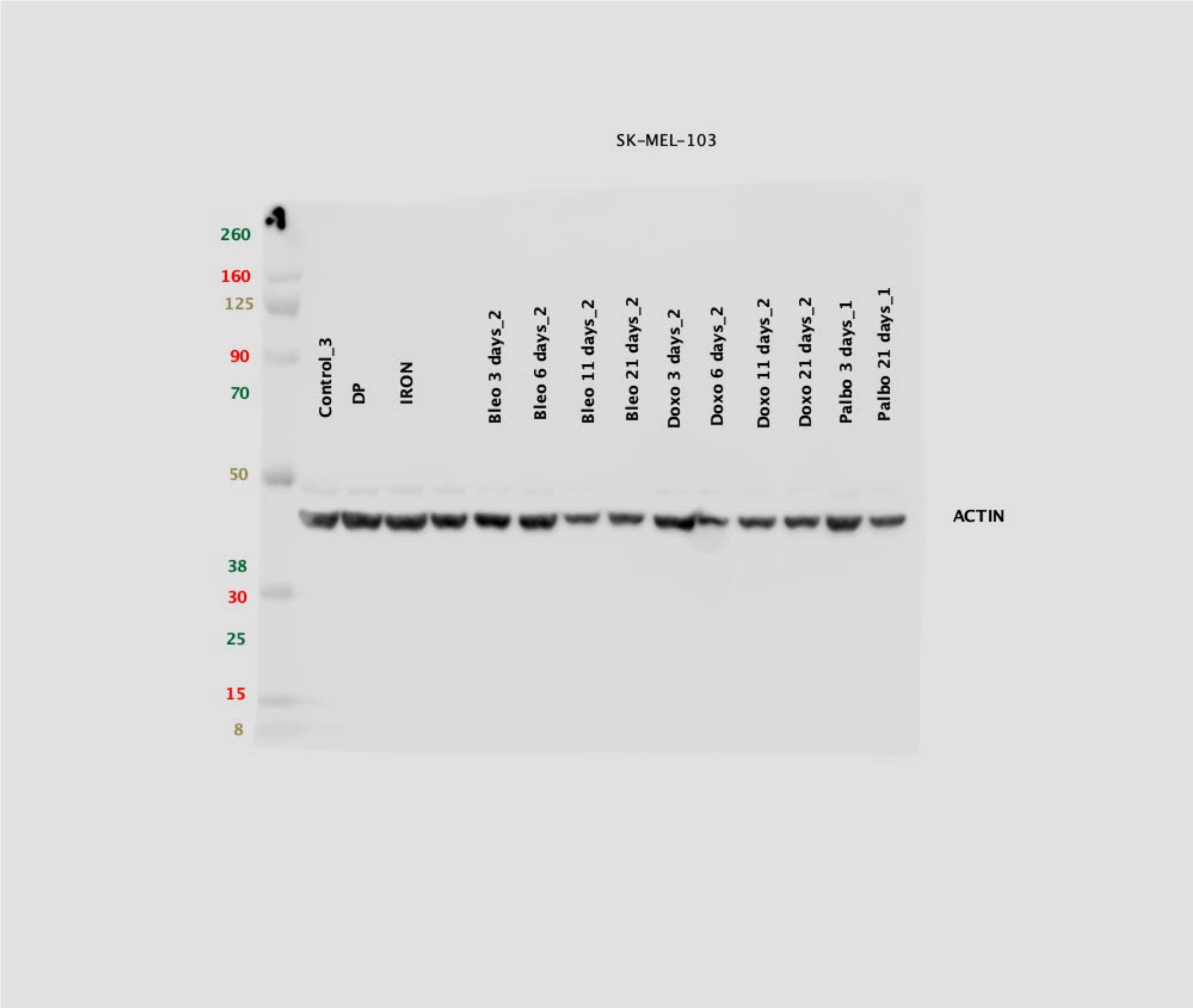

Supplement: Supplementary file 8 — Uncropped labeled western blots that are shown in the manuscript and their replicates that are used for quantification. [file 42255_2023_928_MOESM8_ESM.pdf]
